# Discovery of *N*-(2-Acetamidobenzo[*d*]thiazol-6-yl)-2-phenoxyacetamide Derivatives as Novel Potential BCR-ABL1 Inhibitors Through Structure-Based Virtual Screening

**DOI:** 10.3390/molecules30051065

**Published:** 2025-02-26

**Authors:** Shuaixing Wang, Minyi Wang, Zi Li, Guofeng Xu, Dayan Wang

**Affiliations:** 1 National Institute for Viral Disease Control and Prevention, Chinese Center for Disease Control and Prevention, WHO Collaborating Centre for Reference and Research on Influenza, Key Laboratory for Medical Virology and Viral Diseases, National Health Commission, National Key Laboratory of Intelligent Tracking and Forecasting for Infectious Disease, Beijing 102206, China; wangsx@ivdc.chinacdc.cn (S.W.); miny_wang@163.com (M.W.); lizi@ivdc.chinacdc.cn (Z.L.); 2 State Key Laboratory of Natural and Biomimetic Drugs, School of Pharmaceutical Sciences, Peking University, Beijing 100083, China; xuguofeng951006@outlook.com

**Keywords:** BCR-ABL, structure–activity relationship, combination therapy

## Abstract

BCR-ABL1 kinase is a critical driver of chronic myeloid leukemia (CML) pathophysiology. The approval of allosteric inhibitor asciminib brings new hope for overcoming drug resistance caused by mutations in the ATP-binding site. To expand the chemical diversity of BCR-ABL1 kinase inhibitors with positive anti-tumor effect with asciminib, structure-based virtual screening and molecular dynamics simulations were employed to discover novel scaffolds. This approach led to the identification of a series of *N*-(2-acetamidobenzo[*d*]thiazol-6-yl)-2-phenoxyacetamide derivatives as new BCR-ABL1 inhibitors. The most potent compound, **10m**, demonstrated inhibition of BCR-ABL-dependent signaling and showed an anti-tumor effect against K562 cells, with an IC_50_ value of 0.98 μM. Compound **10m** displayed powerful synergistic anti-proliferation and pro-apoptotic effects when combined with asciminib, highlighting its potential as a promising lead for the development of potential BCR-ABL inhibitors.

## 1. Introduction

Chronic myeloid leukemia (CML) is a hematological malignancy characterized by the presence of the Philadelphia chromosome, resulting from a reciprocal translocation between chromosomes 9 and 22, namely t (9;22) (q34;q11) chromosome translocation. This translocation generates the *Bcr-Abl1* fusion gene, which encodes a constitutively active tyrosine kinase (BCR-ABL1) that drives aberrant cell proliferation and impaired apoptosis [1,2]. CML accounts for approximately 15% of all adult leukemias and has a global incidence rate of 1–2 cases per 100,000 annually [3]. The BCR–ABL1 kinase drives multiple signaling pathways, including cell growth, survival, invasion and angiogenesis and tumor initiation and progression, indicating poor prognosis in CML patients and acute lymphoblastic leukemia (ALL) patients [4].

**Figure 1:**
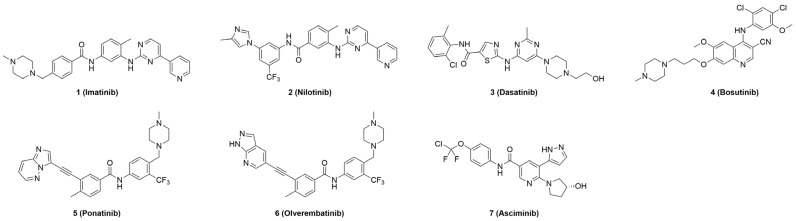
Representative small-molecular BCR-ABL1 inhibitors.

The advent of tyrosine kinase inhibitors (Figure 1), notably imatinib (1) [5], has transformed CML into a manageable chronic disease, with most patients achieving near-normal life expectancy when treated during the chronic phase [6]; however, several therapeutic challenges remain. Resistance to treatment is a major concern, particularly in advanced disease stages, which spurred the discovery of second-generation inhibitors, including nilotinib (2) [7], dasatinib (3) [8], bosutinib (4) [9], etc. The T315I “gatekeeper” mutation is among the most refractory, rendering the majority of ATP-competitive TKIs ineffective. Second-generation inhibitors such as dasatinib and nilotinib have improved potency against several resistant mutations; however, their efficacy against T315I and certain other mutations remains limited [10,11]. Third-generation inhibitors like ponatinib (5) [12] and olverembatinib (6) [13] address this gap but are associated with severe toxicity risks, including cardiovascular complications [14,15]. Therapeutic strategies are being pursued to overcome these limitations. Asciminib (ABL001, 7) [16], the first allosteric BCR-ABL1 inhibitor demonstrates activity against TKI-resistant mutations, including T315I, and offers a potentially improved safety profile by minimizing off-target kinase inhibition. However, rapid development of drug resistance also has been observed [17]. One promising approach is the combination therapy dual-targeting the ATP-binding site and myristoyl pocket of BCR-ABL1, which provides an alternative mechanism of action by restoring the protein’s auto-inhibitory conformation. In vitro studies demonstrated additive effects of asciminib when combined with imatinib, nilotinib, or dasatinib [16,17,18]. Asciminib also exhibited anti-tumor activity against BCR-ABL1 point mutants and suppressed the emergence of resistant point mutations when combined with nilotinib or ponatinib [19]. However, negative cooperativity between imatinib and asciminib has been observed in a structural study [20], underscoring the importance of rational selection of double-drug combinations with positive cooperativity.

In this work, structure-based in silico methods were employed to discover an orthosteric inhibitor bearing synergistic anti-tumor potency with asciminib. Hit compound **A8** exhibited moderate growth inhibition against Ba/F3 (BCR-ABL1) cells, with a GI_50_ value of 6.4 μM. 23 *N*-(benzo[*d*]thiazol-2-yl)acetamide derivatives were designed and synthesized, and their anti-tumor activities against Ba/F3 and K562 cells as well as BCR-ABL1-dependent signaling inhibitory potency were evaluated. The most potent compound, **10m**, displayed powerful anti-tumor potency in vitro, which may serve as a promising lead compound for further optimization.

**Scheme 1:**
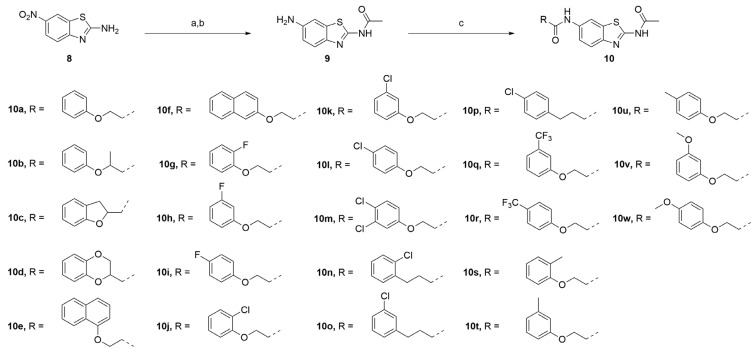
Synthesis of Compounds **10a**–**10w**. Reagents and conditions: (**a**) acetic anhydride, pyridine, 120 °C, 84%; (**b**) sodium dithionite, NH_4_OH, r.t, 78%.; (**c**) acid, BOP, DIPEA, DMF, 40 °C, 36–86%.

The series of derivatives were prepared as shown in Scheme 1. The amine 8 was treated with acetic anhydride in pyridine to afford an intermediate, which was hydrogenated to give compound **9**. Amide condensation of compound **9** with various acid afforded compounds **10a**–**w**.

## 2. Results

### 2.1. Structured-Based Virtual Screening

**Figure 2:**
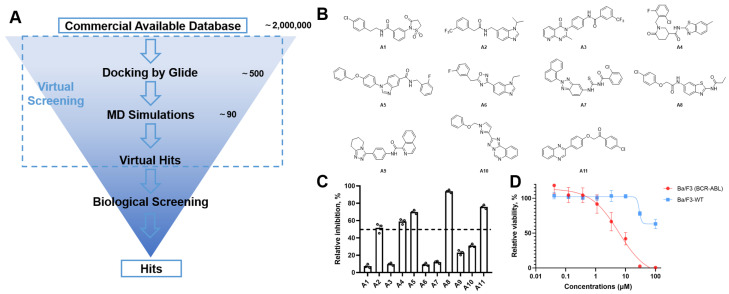
(**A**) Schematic flow of the virtual screening campaign. (**B**) Chemical structure of virtual hits **A1**–**A11**. (**C**) The anti-proliferative effect of hit compounds on Ba/F3 (BCR-ABL1) cells was evaluated by CCK8. Data are represented as mean ± SD (n = 3). (**D**) The dose-dependency curves against parental Ba/F3 cells and Ba/F3 (BCR-ABL1) cells of compound **A8**.

Although imatinib or nilotinib displayed stronger synergic anti-proliferative effects with asciminib in vitro [17], a structural study has revealed that double-drugging with an “open” conformation binder resulted in an energetically frustrated ABL structure [20]. In order to discover new inhibitors with positive cooperativity with asciminib, a fully “closed” conformation of ABL (PDB id: 8SSN) was used. Commercial databases, including the SPECS database and ChemDiv database, approximately 2 million molecules, were docked into the kinase domain. The top-scored hits were combined and the common hits were selected. As drawn in Figure 2A, through a cascade of drug-likeness filtering, standard precision (SP) docking, extra precision (XP) docking, structural clustering, MD simulations and visual inspection, 11 structurally diverse compounds, namely **A1***–***A11** (Figure 2B), were cherry-picked and purchased from the ChemDiv chemical library. These compounds were evaluated by parental Ba/F3 cells and Ba/F3 cells expressing BCR-ABL1 kinases, respectively. Initial inhibitory activity was conducted for each compound at concentrations of 50 μM (Figure 2C). Five out of eleven candidates showed inhibition against Ba/F3 (BCR-ABL1) cells (Figure S1). Among them, compound **A8** showed inhibition potency at a lower concentration of 10 μM. We further measured the dose curves, and compound **A8** displayed moderate antiproliferative activity with a GI_50_ of 6.4 μM (Figure 2D).

### 2.2. Similarity Search

Docking studies revealed that compound **A8** formed crucial hydrogen bonds with Met-337 in the hinge region (Figure S2). To validate the predicted binding mode, a two-dimensional (2D) similarity search was conducted based on the benzothiazole moiety of **A8** from the ChemDiv database, yielding compounds **A12**–**A15**. As shown in Table 1, **A12** showed nearly two-fold decreased antiproliferative activity compared to **A8** (10.7 μM vs. 6.4 μM), suggesting chlorine atom was preferred. The activity of compound **A13** further decreased, suggesting the benzoyl group might clash with the hinge region. Compounds **A14** and **A15** showed a similar pattern. Compound **A14** with methylsulfonyl displayed improved activity with a GI_50_ of 1.2 μM and was also cytotoxic. These findings suggested that the chemical structure of **A8** might serve as a starting point for further exploration of novel BCR-ABL1 inhibitors.

### 2.3. Molecular Design

**Figure 3:**
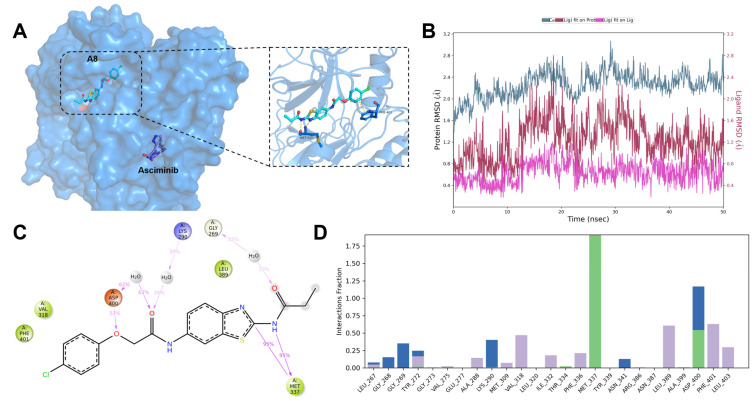
Molecular design of compound **A8** as a novel BCR-ABL1 inhibitor. (**A**) The predicted binding modes of **A8** and asciminib with the BCR-ABL crystal structure (PDB id: 8SSN). Hydrogen bonds were indicated by yellow dashed lines. (**B**) A 50-ns molecular dynamics simulation was conducted using Desmond. The stability of the ligand-receptor complex was evaluated through root mean-square deviation (RMSD) analysis of: (i) heavy atoms of compound **A8** (magenta line), (ii) interacting side chains of the receptor (blue line), and (iii) heavy atoms of compound **A8** relative to the kinase backbone atoms (red line). (**C**,**D**) Protein–ligand contact histogram of compound **A8** and the corresponding 2D diagram predicted through MD simulations, including red ball: Charged (negative), blue ball: Charged (positive), gray ball: Glycine and green ball: Hydrophobic. The percentage value suggests that for X% of the simulation time, the specific interaction is maintained.

The docking study showed that compound **A8** was embedded effectively well in the ATP-binding site, making polar contacts with Met-337 in the hinge region. The chlorobenzene moiety occupied a hydrophobic cavity (Figure 3A). To validate the predicted binding mode (Figure 3C,D), a 50-ns molecular dynamics (MD) simulation was performed. The simulation results demonstrated remarkable stability of compound **A8** within the ATP-binding site, as evidenced by consistently low RMSD values. Notably, the benzo-thiazole scaffold maintained two characteristic hydrogen bonds with Met-337 throughout 99% of the simulation trajectory, further confirming the stability of the binding conformation. The ether oxygen of compound **A8** formed a hydrogen bond with Asp-400, which was maintained for 53% of the simulation time. It was noticeable that a π-π interaction between the chlorobenzene moiety and Phe-401 was formed. We hypothesized that the strength of the π-π interaction is crucial for maintaining the protein’s “closed” conformation. Thus, the following two methods to study the structure–activity relationship (SAR) have been adopted: (1) small substituents were introduced to the benzene ring or (2) a conformation–restriction strategy was explored.

### 2.4. SAR Study

Guided by the predicted binding mode, we systematically explored the structure–activity relationship of this compound series (Table 2). First, we employed a conformation–restriction strategy, resulting in the loss of activity against Ba/F3 (BCR-ABL1) cells (compounds **10b–d**). Derivative **10f**, rather than **10e**, which incorporated a naphthyl group, showed a slight increase in inhibitory activity. The decline of **10e** may be attributed to increased polarity and steric hindrance. As shown in Figure S3A, compound **10f** formed an edge-to-face π-π stacking interaction with Phe-401. These findings indicated that the angle and distance of the π-π interaction were important.

Next, small substituents were introduced into the benzene ring. The introduction of electron-withdrawing groups significantly enhanced BCR-ABL1 inhibitory activity (**10g**–**m**), whereas derivatives with an electron-donating methyl group showed a slight reduction in cellular potency (**10s**–**w**). Additionally, a directional “sigma-hole” formed by the chlorobenzene moiety in compound **10m** led to increased binding affinity (Figures S3B and S4). Our analysis revealed that the presence of an oxygen atom was essential for inhibitory activity, as evidenced by the comparison between **10j**–**l** and **10 n**–**p**, which showed improved potency compared to **10a**. Compounds **10n**–**p** exhibited significantly reduced activity, likely due to the loss of the hydrogen bond with Asp-400. Notably, compounds **10q** and **10r** bearing the electron-withdrawing trifluoromethyl group were not very tolerant, which may be attributed to their relatively large volume. Building on these findings, dose-dependency curves of compounds **10f** and **10m** against multiple leukemia cell lines were determined. As shown in Table 3, compounds **10f** and **10m** showed an anti-tumor effect against Ba/F3 (BCR-ABL1) cells, with IC_50_ values of 2.2 and 0.63 μM, respectively. Compound **10m** also showed limited activity against Ba/F3 (BCR-ABL^T315I^) cells.

**Figure 4:**
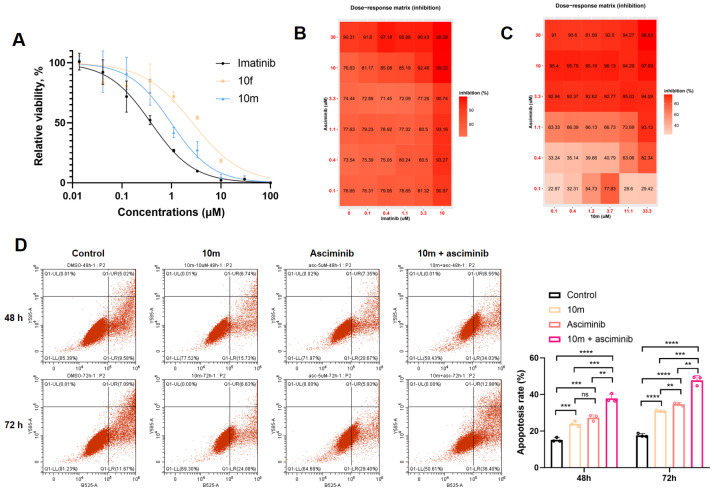
Synergy studies were performed using compound **10m** in combination with asciminib. (**A**) K562 cells were incubated with the compound combinations across a dose range for 72 h, and the level of cell growth relative to DMSO-treated cells was determined. (**B**,**C**) Synergy studies were performed using asciminib in combination with imatinib (**B**) or **10m** (**C**). K562 cells were incubated with the compound combinations across a dose range for 48 h. The level of cell growth relative to DMSO-treated cells was determined. (**D**) The apoptotic effects on K562 cells were assessed using flow cytometry following treatment with compound **10m** (10 μM) or asciminib (5 μM) for 48 or 72 h. Experimental results are presented as mean ± SEM, with statistical significance determined by Student’s *t*-test (** *p* < 0.01, *** *p* < 0.001, **** *p* < 0.0001).

With the aim of confirming the cooperative potency, K562 cells were treated with compound **10m** and asciminib. As shown in Figure 4A, compounds **10m** and **10f** inhibited K562 cells with GI_50_ values of 0.98 and 2.7 μM, respectively. The synergistic effect of the drug combination was evaluated using the ZIP synergy score and calculated using SynergyFinder 3.0 [21]. As shown in Figure 4B,C, compound **10m** displayed powerful synergistic activity with asciminib at 33.3 and 11.1 μM, with synergy score ranging from 10 to 58 (Table S3). However, when the concentration of imatinib was below 3.33 μM, the synergy scores were consistently below −10 (Table S2). Imatinib showed antagonistic effect in combination with asciminib, which was consistent with imatinib’s allosteric activator effect [22]. Furthermore, treatment with either compound **10m** or asciminib alone significantly induced apoptosis in K562 cells, as quantitatively assessed through Annexin V/propidium iodide (PI) dual staining assays. Notably, the combination of **10m** and asciminib demonstrated a synergistic effect, resulting in substantially enhanced apoptosis induction compared to either single agent treatment. These results strongly suggest the therapeutic potential of combination therapy utilizing both compounds.

**Figure 5:**
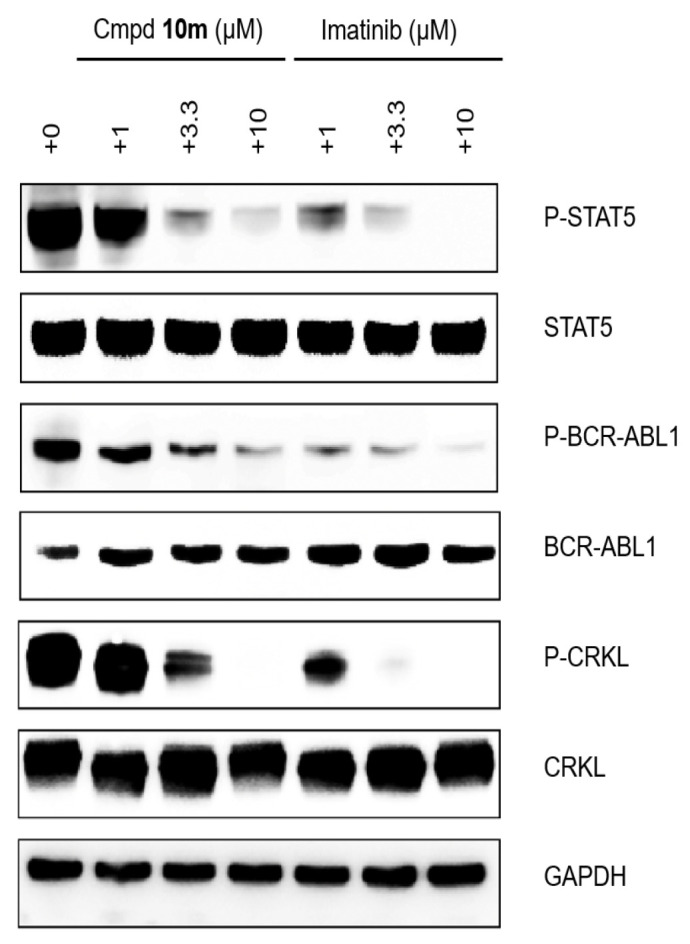
K562 cells were incubated with a range of compound concentrations for 1 h and immunoblots were run to detect total BCR–ABL1 and pBCR–ABL1 (Tyr245), total STAT5 and pSTAT5 (Tyr694), total CRKL and pCRKL (Tyr207), and GAPDH as a loading control.

To validate the inhibitory effects on BCR-ABL-mediated signaling pathways, we performed cellular assays using K562 cells treated with increasing concentrations of compound **10m** or imatinib. The phosphorylation status of key downstream signaling molecules, including ABL1, STAT5, and CRKL was quantitatively analyzed through Western blot analysis (Figure 5 and Figure S3). A dose-dependent inhibition of p-ABL1, p-STAT5 and p-CRKL were observed. Compound **10m** potently inhibited the BCR-ABL-dependent signaling pathway at 3.3 and 10 μM.

## 3. Discussion

The development of orthosteric BCR-ABL1 kinase inhibitors, such as imatinib, has significantly improved the prognosis of patients with CML or ALL [23,24]. The advancement of second- and third-generation BCR-ABL1 inhibitors has led to faster, deeper responses that predict superior outcomes [14,25]. With the breakthrough of the allosteric inhibitor asciminib [17], treatment is increasingly focused on identifying effective combination regimens. Although the simultaneous therapeutic blockade of orthosteric and allosteric inhibitors has shown anti-tumor activity against BCR-ABL1 point mutants and suppressed the emergence of resistant point mutations in vivo [18,19], structural studies have revealed that dual treatment with imatinib results in an energetically frustrated ABL structure. To address this, a fully inactive Abl structure [20] was applied to screen orthosteric BCR-ABL1 kinase inhibitors with synergistic effects in combination with asciminib. This approach led to the discovery of compound **A8**, which features a novel 2-acetamidobenzo[*d*]thiazol scaffold. Based on the **A8** scaffold, 23 derivatives were synthesized and evaluated on Ba/F3 (BCR-ABL1) cells. The most potent compound, **10m**, demonstrated anti-proliferative activity against Ba/F3 (BCR-ABL1) and K562 cells, with GI_50_ values of 0.63 and 0.98 μM, respectively. Compound **10m** also exhibited potent inhibition of BCR-ABL signaling and showed synergistic effects on cell growth and apoptosis when combined with asciminib. These findings suggest that compound **10m** may serve as a promising starting point for the development of dual-targeting therapeutic drugs for BCR-ABL1.

## 4. Experimental Section

### 4.1. Chemistry

General. All starting materials were obtained from commercial sources and used without additional purification. Unless otherwise specified, all reactions were performed without purification. Reaction progress was monitored by thin-layer chromatography (TLC) using silica gel plates (Yantai Jiangyou silica gel development Co. Ltd., HSGF254, Yantai, China), with visualization under UV light (254 nm or 365 nm) or by iodine vapor staining. Solvents were removed by rotary evaporation under reduced pressure at 40–45 °C. Nuclear magnetic resonance spectra (^1^H NMR and ^13^C NMR) were recorded on a Bruker 400 MHz spectrometer. Purification was carried out by flash column chromatography using silica gel (Qingdao Haiyang Chemical Co. Ltd., ZCX-II, 200–300 mesh, Qingdao, China) on a SepaBean^®^ machine U100 preparative liquid chromatography system. Compound purity was determined by HPLC analysis using an Agilent (Santa Clara, CA, USA) 1260 system (G7111A Quat pump and G7114A VWD detector) equipped with a Poroshell 120 EC-C18 reversed-phase column (4 μm) at 254 nM.

#### 4.1.1. HPLC Method

A linear gradient program using water (solvent A) and acetonitrile (solvent B); t = 0–2 min, 10% B, t = 18 min, 90% B was employed. The flow rate was 1.2 mL/min and UV detection was set to 254 nm.

#### 4.1.2. General Procedure for Synthesis of Compounds **10a**–**w**

To a solution of acid (1.2 eq) in DMF (3 mL) was added BOP (1.5 eq) and DIPEA (3 eq). The reaction was stirred at 40 °C for 30 min, and then compound **9** (1.0 eq) was added. After the reaction was finished by TLC, the mixture was poured into water and extracted with ethyl acetate. The organic layer was dried over Na_2_SO_4_, filtered, and concentrated. The residue was purified by silica gel column chromatography (DCM/MeOH, 30:1) followed by recrystallization in acetonitrile to provide the desired product.

#### 4.1.3. *N*-(6-Aminobenzo[*d*]thiazol-2-yl)acetamide (**9**)

To a solution of 2-amino-6-nitrobenzothiazole (5.0 g, 25.61 mol) in pyridine (18 mL), acetic anhydride (15 mL, 135.17 mmol) was added. The reaction mixture was heated to 90 °C overnight. The reaction mixture was poured into 2N HCl (200 mL). The solid product that was formed was collected by filtration and washed with water and tert-butyl methyl ether and finally dried to give intermediate *N*-(6-nitrobenzo[*d*]thiazol-2-yl)acetamide as a pale yellow solid (5.1 g, yield 84%). ^1^H NMR (400 MHz, DMSO- *d*_6_ δ 12.76 (s, 1H), 9.02 (d, *J* = 2.4 Hz, 1H), 8.26 (dd, *J* = 8.8, 2.4 Hz, 1H), 7.87 (d, *J* = 8.8 Hz, 1H), 2.25 (s, 3H). ^13^C NMR (101 MHz, DMSO-*d*_6_) δ 170.64, 163.92, 153.92, 143.35, 132.60, 122.19, 120.98, 119.47, 23.30.

To a solution of compound *N*-(6-nitrobenzo[*d*]thiazol-2-yl)acetamide (2.5 g, 10.54 mmol) in NH_4_OH (12 mL), a solution of sodium dithionite (8.8 g, 50.54 mmol) in water (40 mL) was quickly added; the reaction mixture was held at reflux overnight. After cooling, the crude product was filtered off, washed and dried under vacuum to afford compound **9** as a yellowish solid (1.7 g, yield 78%). ^1^H NMR (400 MHz, DMSO-*d*_6_) δ 11.88 (s, 1H), 7.39 (d, *J* = 8.8 Hz, 1H), 6.99 (s, 1H), 6.69 (d, *J* = 8.8 Hz, 1H), 5.14 (s, 2H), 2.14 (s, 3H). ^13^C NMR (101 MHz, DMSO-*d*_6_) δ 169.10, 153.53, 146.16, 140.07, 133.36, 121.27, 114.84, 104.55, 23.13.

#### 4.1.4. *N*-(2-Acetamidobenzo[*d*]thiazol-6-yl)-2-phenoxyacetamide (**10a**)

Following general procedure A, compound **10a** (82 mg, yield 76%) was obtained as a white solid. ^1^H NMR (400 MHz, DMSO- *d*_6_) δ 12.29 (s, 1H), 10.22 (s, 1H), 8.31 (d, *J* = 2.0 Hz, 1H), 7.69 (d, *J* = 8.8 Hz, 1H), 7.61 (dd, *J* = 8.8, 2.0 Hz, 1H), 7.32 (t, *J* = 7.8 Hz, 2H), 7.08–6.93 (m, 3H), 4.72 (s, 2H), 2.19 (s, 3H). ^13^C NMR (101 MHz, DMSO- *d*_6_) δ 169.74, 167.00, 158.29, 157.65, 145.37, 134.73, 132.32, 129.99, 121.67, 120.89, 119.61, 115.16, 112.76, 67.63, 23.19. R_T_ = 10.396 min, purity = 97.11%. Melting point: 156–158 °C.

#### 4.1.5. *N*-(2-Acetamidobenzo[*d*]thiazol-6-yl)-2-phenoxypropanamide (**10b**)

Following general procedure A, compound **10b** (88 mg, yield 72%) was obtained as a white solid. ^1^H NMR (400 MHz, DMSO- *d*_6_) δ 12.28 (s, 1H), 10.26 (s, 1H), 8.29 (d, *J* = 2.0 Hz, 1H), 7.66 (d, *J* = 8.8 Hz, 1H), 7.59 (dd, *J* = 8.8, 2.0 Hz, 1H), 7.29 (tt, *J* = 7.6, 2.0 Hz, 2H), 7.02–6.91 (m, 3H), 4.95–4.85 (m, 1H), 2.19 (s, 3H), 1.57 (dd, *J* = 6.8, 2.0 Hz, 3H). ^13^C NMR (101 MHz, DMSO- *d*_6_) δ 170.49, 169.75, 157.75, 157.66, 145.34, 134.82, 132.29, 130.05, 121.65, 120.86, 119.55, 115.60, 112.69, 74.23, 23.19, 19.16. HRMS (ESI) [M + H]^+^ calcd for C18H18N3O3S: 356.10634; found: 356.10503. R_T_ = 10.722 min, purity = 97.47%. Melting point: 162–164 °C.

#### 4.1.6. *N*-(2-Acetamidobenzo[*d*]thiazol-6-yl)-2,3-dihydrobenzofuran-2-carboxamide (**10c**)

Following general procedure A, compound **10c** (62 mg, yield 60%) was obtained as a white solid. ^1^H NMR (400 MHz, DMSO- *d*_6_) δ 12.29 (s, 1H), 10.29 (s, 1H), 8.31 (d, *J* = 2.0 Hz, 1H), 7.68 (d, *J* = 8.8 Hz, 1H), 7.63 (dd, *J* = 8.8, 2.0 Hz, 1H), 7.25 (d, *J* = 7.6 Hz, 1H), 7.15 (t, *J* = 7.6 Hz, 1H), 6.92–6.81 (m, 2H), 5.34 (dd, *J* = 10.4, 6.8 Hz, 1H), 3.62–3.35 (m, 2H), 2.19 (s, 3H). ^13^C NMR (101 MHz, DMSO- *d*_6_) δ 169.75, 169.48, 159.15, 157.72, 145.47, 134.66, 132.24, 128.41, 126.35, 125.45, 121.42, 120.84, 119.85, 113.05, 109.89, 80.84, 33.47, 23.19. HRMS (ESI) [M + H]^+^ calcd for C18H16N3O3S: 354.09069; found: 354.08949. R_T_ = 11.486 min, purity = 93.3%. Melting point: 163–165 °C.

#### 4.1.7. *N*-(2-Acetamidobenzo[*d*]thiazol-6-yl)-2,3-dihydrobenzo[*b*][1,4]dioxine-2-carboxamide (**10d**)

Following general procedure A, compound **10d** (55 mg, yield 71%) was obtained as a yellowish solid. ^1^H NMR (400 MHz, DMSO- *d*_6_) δ 12.30 (s, 1H), 10.26 (s, 1H), 8.29 (s, 1H), 7.69 (d, *J* = 8.8 Hz, 1H), 7.60 (dd, *J* = 8.8, 2.0 Hz, 1H), 7.09–7.00 (m, 1H), 6.95–6.83 (m, 3H), 5.00 (dd, *J* = 6.0, 2.4 Hz, 1H), 4.54–4.32 (m, 2H), 2.19 (s, 3H). ^13^C NMR (101 MHz, DMSO- *d*_6_) δ 169.77, 165.98, 157.82, 145.56, 143.49, 142.87, 134.42, 132.28, 122.13, 121.99, 120.87, 119.81, 117.78, 117.51, 113.07, 73.14, 65.28, 23.19. HRMS (ESI) [M + H]^+^ calcd for C18H16N3O4S: 370.08560; found: 370.08429. R_T_ = 10.828 min, purity = 97.21%. Melting point: 166–168 °C.

#### 4.1.8. *N*-(2-Acetamidobenzo[*d*]thiazol-6-yl)-2-(naphthalen-1-yloxy)acetamide (**10e**)

Following general procedure A, compound **10e** (83 mg, yield 75%) was obtained as a white solid. ^1^H NMR (400 MHz, DMSO- *d*_6_) δ 12.31 (s, 1H), 10.37 (s, 1H), 8.43–8.29 (m, 2H), 7.88 (dd, *J* = 6.4, 3.6 Hz, 1H), 7.71 (d, *J* = 8.8 Hz, 1H), 7.64 (dd, *J* = 8.8, 2.4 Hz, 1H), 7.59–7.49 (m, 3H), 7.42 (t, *J* = 8.0 Hz, 1H), 6.97 (d, *J* = 7.6 Hz, 1H), 4.96 (s, 2H), 2.21 (s, 3H). ^13^C NMR (101 MHz, DMSO- *d*_6_) δ 169.76, 166.85, 157.72, 153.96, 145.43, 134.79, 134.58, 132.39, 127.88, 127.03, 126.52, 125.83, 125.39, 122.48, 121.15, 120.93, 119.68, 112.88, 106.08, 68.17, 23.20. HRMS (ESI) [M + H]^+^ calcd for C21H18N3O3S: 392.10634; found: 392.10484. R_T_ = 12.024 min, purity = 94.14%. Melting point: 174–175 °C.

#### 4.1.9. *N*-(2-Acetamidobenzo[*d*]thiazol-6-yl)-2-(naphthalen-2-yloxy)acetamide (**10f**)

Following general procedure A, compound **10f** (66 mg, yield 68%) was obtained as a white solid. ^1^H NMR (400 MHz, DMSO- *d*_6_) δ 12.29 (s, 1H), 10.29 (s, 1H), 8.33 (s, 1H), 7.92–7.78 (m, 3H), 7.70 (d, *J* = 8.8 Hz, 1H), 7.64 (d, *J* = 9.6 Hz, 1H), 7.46 (t, *J* = 7.6 Hz, 1H), 7.40–7.31 (m, 3H), 4.86 (s, 2H), 2.19 (s, 3H). ^13^C NMR (101 MHz, DMSO- *d*_6_) δ 169.76, 166.85, 157.68, 156.19, 145.40, 134.74, 134.51, 132.33, 129.87, 129.25, 128.01, 127.24, 126.97, 124.36, 120.90, 119.69, 119.15, 112.85, 107.75, 67.77, 23.20. HRMS (ESI) [M + H]^+^ calcd for C21H18N3O3S: 392.10634; found: 392.10489. R_T_ = 11.883 min, purity = 99.89%. Melting point: 174–176 °C.

#### 4.1.10. *N*-(2-Acetamidobenzo[*d*]thiazol-6-yl)-2-(2-fluorophenoxy)acetamide (**10g**)

Following general procedure A, compound **10g** (52 mg, yield 60%) was obtained as a yellowish solid. ^1^H NMR (400 MHz, DMSO- *d*_6_) δ 12.28 (s, 1H), 10.30 (s, 1H), 8.29 (s, 1H), 7.68 (d, *J* = 8.8 Hz, 1H), 7.58 (d, *J* = 8.8 Hz, 1H), 7.24 (dd, *J* = 12.0, 8.0 Hz, 1H), 7.19–7.07 (m, 2H), 7.05–6.94 (m, 1H), 4.82 (s, 2H), 2.19 (s, 3H). ^13^C NMR (101 MHz, DMSO- *d*_6_) δ 168.14 (d, *J* = 320.7 Hz), 157.67, 153.41, 150.98, 146.39 (d, *J* = 10.3 Hz), 145.34, 134.74, 132.38, 125.21 (d, *J* = 3.6 Hz), 122.20 (d, *J* = 6.8 Hz), 120.94, 119.38, 116.67 (d, *J* = 17.8 Hz), 115.73, 112.56, 68.33, 23.18. ^19^F NMR (376 MHz, DMSO- *d*_6_) δ -134.19. HRMS (ESI) [M + H]^+^ calcd for C17H15FN3O3S: 360.08127; found: 360.07992. R_T_ = 10.465 min, purity = 98.42%. Melting point: 164–166 °C.

#### 4.1.11. *N*-(2-Acetamidobenzo[*d*]thiazol-6-yl)-2-(3-fluorophenoxy)acetamide (**10h**)

Following general procedure A, compound **10h** (84 mg, yield 78%) was obtained as a white solid. ^1^H NMR (400 MHz, DMSO- *d*_6_) δ 12.29 (s, 1H), 10.23 (s, 1H), 8.31 (s, 1H), 7.69 (d, *J* = 8.8 Hz, 1H), 7.61 (d, *J* = 8.8 Hz, 1H), 7.34 (q, *J* = 8.0 Hz, 1H), 6.94–6.85 (m, 2H), 6.85–6.76 (m, 1H), 4.76 (s, 2H), 2.19 (s, 3H). ^13^C NMR (101 MHz, DMSO- *d*_6_) 168.15 (d, *J* = 318.6 Hz), 164.55, 162.14, 159.75 (d, *J* = 11.1 Hz), 157.71, 145.41, 134.65, 132.35, 131.19 (d, *J* =10.1 Hz), 120.89, 119.60, 112.79, 111.41 (d, *J* = 2.7 Hz), 108.31 (d, *J* = 21.0 Hz), 102.94 (d, *J* = 25.0 Hz). ^19^F NMR (376 MHz, DMSO- *d*_6_) δ -111.61. HRMS (ESI) [M + H]^+^ calcd for C17H15FN3O3S: 360.08127; found: 360.07997. R_T_ = 10.691 min, purity = 94.44%. Melting point: 163–165 °C.

#### 4.1.12. *N*-(2-Acetamidobenzo[*d*]thiazol-6-yl)-2-(4-fluorophenoxy)acetamide (**10i**)

Following general procedure A, compound **10i** (82 mg, yield 80%) was obtained as a white solid. ^1^H NMR (400 MHz, DMSO- *d*_6_) δ 12.29 (s, 1H), 10.21 (s, 1H), 8.29 (d, *J* = 2.0 Hz, 1H), 7.68 (d, *J* = 8.8 Hz, 1H), 7.60 (dd, *J* = 8.8, 2.0 Hz, 1H), 7.22–7.12 (m, 2H), 7.10–6.99 (m, 2H), 4.70 (s, 2H), 2.19 (s, 3H). ^13^C NMR (101 MHz, DMSO- *d*_6_) δ 169.75, 166.88, 156.15 (d, *J* = 302.8 Hz), 145.37, 134.69, 132.30, 120.89, 119.62, 116.61, 116.52, 116.46, 116.23, 112.78, 68.20, 23.19. ^19^F NMR (376 MHz, DMSO- *d*_6_) δ -123.25. HRMS (ESI) [M + H]^+^ calcd for C17H15FN3O3S: 360.08127; found: 360.07998. R_T_ = 10.342 min, purity = 99.41%. Melting point: 164–166 °C.

#### 4.1.13. *N*-(2-Acetamidobenzo[*d*]thiazol-6-yl)-2-(2-chlorophenoxy)acetamide (**10j**)

Following general procedure A, compound **10j** (98 mg, yield 86%) was obtained as a yellowish solid. ^1^H NMR (400 MHz, DMSO- *d*_6_) δ 12.29 (s, 1H), 10.28 (s, 1H), 8.29 (d, *J* = 2.0 Hz, 1H), 7.68 (d, *J* = 8.8 Hz, 1H), 7.56 (dd, *J* = 8.8, 2.0 Hz, 1H), 7.46 (dd, *J* = 8.0, 1.6 Hz, 1H), 7.30 (td, *J* = 8.0, 1.6 Hz, 1H), 7.11 (d, *J* = 8.4 Hz, 1H), 6.99 (td, *J* = 8.0, 1.6 Hz, 1H), 4.86 (s, 2H), 2.19 (s, 3H). ^13^C NMR (101 MHz, DMSO- *d*_6_) δ 169.75, 166.35, 157.67, 153.93, 145.33, 134.73, 132.41, 130.56, 128.74, 122.59, 121.92, 120.99, 119.24, 114.55, 112.42, 68.16, 23.19. HRMS (ESI) [M + H]^+^ calcd for C17H15ClN3O3S: 376.05172; found: 376.05056. R_T_ = 10.768 min, purity = 99.98%. Melting point: 162–163 °C.

#### 4.1.14. *N*-(2-Acetamidobenzo[*d*]thiazol-6-yl)-2-(3-chlorophenoxy)acetamide (**10k**)

Following general procedure A, compound **10k** (66 mg, yield 58%) was obtained as a white solid. ^1^H NMR (400 MHz, DMSO- *d*_6_) δ 12.29 (s, 1H), 10.22 (s, 1H), 8.30 (d, *J* = 2.0 Hz, 1H), 7.69 (d, *J* = 8.8 Hz, 1H), 7.60 (dd, *J* = 8.8, 2.0 Hz, 1H), 7.33 (t, *J* = 8.2 Hz, 1H), 7.13 (t, *J* = 2.2 Hz, 1H), 7.02 (ddd, *J* = 10.9, 8.0, 2.2 Hz, 2H), 4.78 (s, 2H), 2.19 (s, 3H). ^13^C NMR (101 MHz, DMSO- *d*_6_) δ 169.74, 166.55, 159.23, 157.71, 145.41, 134.64, 134.14, 132.35, 131.35, 121.63, 120.90, 119.60, 115.51, 114.12, 112.79, 67.73, 23.19. HRMS (ESI) [M + H]^+^ calcd for C17H15ClN3O3S: 376.05172; found: 376.05059. R_T_ = 11.407 min, purity = 97.07%. Melting point: 168–169 °C.

#### 4.1.15. *N*-(2-Acetamidobenzo[*d*]thiazol-6-yl)-2-(4-chlorophenoxy)acetamide (**10l**)

Following general procedure A, compound **10l** (70 mg, yield 64%) was obtained as a white solid. ^1^H NMR (400 MHz, DMSO- *d*_6_) δ 12.29 (s, 1H), 10.23 (s, 1H), 8.29 (d, *J* = 2.0 Hz, 1H), 7.68 (d, *J* = 8.8 Hz, 1H), 7.59 (dd, *J* = 8.8, 2.0 Hz, 1H), 7.44–7.32 (m, 2H), 7.12–6.98 (m, 2H), 4.74 (s, 2H), 2.19 (s, 3H). ^13^C NMR (101 MHz, DMSO- *d*_6_) δ 169.75, 166.67, 157.68, 157.18, 145.38, 134.67, 132.32, 129.73, 125.38, 120.90, 119.59, 116.98, 112.76, 67.82, 23.19. HRMS (ESI) [M + H]^+^ calcd for C17H15ClN3O3S: 376.05172; found: 376.05050. R_T_ = 11.327 min, purity = 98.07%. Melting point: 169–171 °C.

#### 4.1.16. *N*-(2-Acetamidobenzo[*d*]thiazol-6-yl)-2-(3,4-dichlorophenoxy)acetamide (**10m**)

Following general procedure A, compound **10m** (72 mg, yield 68%) was obtained as a white solid. ^1^H NMR (400 MHz, DMSO- *d*_6_) δ 12.29 (s, 1H), 10.23 (s, 1H), 8.28 (d, *J* = 2.0 Hz, 1H), 7.69 (d, *J* = 8.8 Hz, 1H), 7.62–7.54 (m, 2H), 7.34 (d, *J* = 2.8 Hz, 1H), 7.06 (dd, *J* = 8.8, 2.8 Hz, 1H), 4.79 (s, 2H), 2.19 (s, 3H). ^13^C NMR (101 MHz, DMSO- *d*_6_) δ 169.76, 166.33, 157.80, 157.72, 145.42, 134.59, 132.34, 131.99, 131.45, 123.52, 120.92, 119.59, 117.41, 116.07, 112.79, 67.91, 23.20. HRMS (ESI) [M + H]^+^ calcd for C17H14Cl2N3O3S: 410.01274; found: 410.01160. R_T_ = 8.964 min, purity = 99.98%. Melting point: 172–174 °C.

#### 4.1.17. *N*-(2-Acetamidobenzo[*d*]thiazol-6-yl)-3-(2-chlorophenyl)propanamide (**10n**)

Following general procedure A, compound **10n** (52 mg, yield 60%) was obtained as a white solid. ^1^H NMR (400 MHz, DMSO- *d*_6_) δ 12.26 (s, 1H), 10.09 (s, 1H), 8.29 (d, *J* = 2.0 Hz, 1H), 7.65 (d, *J* = 8.8 Hz, 1H), 7.50 (dd, *J* = 8.8, 2.0 Hz, 1H), 7.40 (ddd, *J* = 15.6, 7.6, 2.0 Hz, 2H), 7.32–7.20 (m, 2H), 3.04 (t, *J* = 7.6 Hz, 2H), 2.67 (t, *J* = 7.6 Hz, 2H), 2.19 (s, 3H). ^13^C NMR (101 MHz, DMSO- *d*_6_) δ 170.34, 169.68, 157.34, 144.90, 138.90, 135.60, 133.40, 132.36, 131.02, 129.70, 128.49, 127.75, 120.88, 118.97, 111.92, 36.34, 29.03, 23.18. R_T_ = 11.398 min, purity = 99.65%. Melting point: 165–166 °C.

#### 4.1.18. *N*-(2-Acetamidobenzo[*d*]thiazol-6-yl)-3-(3-chlorophenyl)propanamide (**10o**)

Following general procedure A, compound **10o** (90 mg, yield 74%) was obtained as a white solid. ^1^H NMR (400 MHz, DMSO- *d*_6_) δ 12.27 (s, 1H), 10.05 (s, 1H), 8.26 (d, *J* = 2.0 Hz, 1H), 7.64 (d, *J* = 8.8 Hz, 1H), 7.48 (dd, *J* = 8.8, 2.0 Hz, 1H), 7.36–7.20 (m, 4H), 2.94 (t, *J* = 7.6 Hz, 2H), 2.66 (t, *J* = 7.6 Hz, 2H), 2.19 (s, 3H). ^13^C NMR (101 MHz, DMSO- *d*_6_) δ 170.51, 169.69, 157.34, 144.89, 144.28, 135.59, 133.37, 132.36, 130.61, 128.67, 127.53, 126.43, 120.90, 118.94, 111.88, 37.94, 30.82, 23.19. HRMS (ESI) [M + H]^+^ calcd for C18H17ClN3O2S: 374.07245; found: 374.07135. R_T_ = 11.411 min, purity = 95.23%. Melting point: 162–164 °C.

#### 4.1.19. *N*-(2-Acetamidobenzo[*d*]thiazol-6-yl)-3-(4-chlorophenyl)propanamide (**10p**)

Following general procedure A, compound **10p** (71 mg, yield 63%) was obtained as a white solid. ^1^H NMR (400 MHz, DMSO- *d*_6_) δ 12.26 (s, 1H), 10.05 (s, 1H), 8.27 (d, *J* = 2.0 Hz, 1H), 7.64 (d, *J* = 8.8 Hz, 1H), 7.48 (dd, *J* = 8.8, 2.0 Hz, 1H), 7.38–7.24 (m, 4H), 2.92 (t, *J* = 7.6 Hz, 2H), 2.64 (t, *J* = 7.6 Hz, 2H), 2.19 (s, 3H). ^13^C NMR (101 MHz, DMSO- *d*_6_) δ 170.55, 169.68, 157.33, 144.88, 140.68, 135.61, 132.35, 131.03, 130.64, 128.69, 120.89, 118.94, 111.87, 38.10, 30.55, 23.18. HRMS (ESI) [M + H]^+^ calcd for C18H17ClN3O2S: 374.07245; found: 374.07117. R_T_ = 11.329 min, purity = 99.1%. Melting point: 164–166 °C.

#### 4.1.20. *N*-(2-Acetamidobenzo[*d*]thiazol-6-yl)-2-(3-(trifluoromethyl)phenoxy) acetamide (**10q**)

Following general procedure A, compound **10q** (42 mg, yield 36%) was obtained as a white solid. ^1^H NMR (400 MHz, DMSO- *d*_6_) δ 12.30 (s, 1H), 10.26 (s, 1H), 8.30 (d, *J* = 2.0 Hz, 1H), 7.69 (d, *J* = 8.8 Hz, 1H), 7.61 (dd, *J* = 8.8, 2.0 Hz, 1H), 7.55 (t, *J* = 8.0 Hz, 1H), 7.40–7.29 (m, 3H), 4.85 (s, 2H), 2.19 (s, 3H). ^13^C NMR (101 MHz, DMSO- *d*_6_) δ 169.75, 166.50, 158.58, 157.72, 145.44, 134.61, 132.35, 131.20, 130.72 (d, *J* = 31.6 Hz), 124.44 (d, *J* = 270.7 Hz), 120.90, 119.63, 119.20, 118.15 (q, *J* = 3.8 Hz), 112.84, 112.19 (q, *J* = 3.8 Hz), 67.71, 23.17. ^19^F NMR (376 MHz, DMSO- *d*_6_) δ -61.15. HRMS (ESI) [M + H]^+^ calcd for C18H15F3N3O3S: 410.07807; found: 410.07646. R_T_ = 10.481 min, purity = 98.96%. Melting point: 170–172 °C.

#### 4.1.21. *N*-(2-Acetamidobenzo[*d*]thiazol-6-yl)-2-(4-(trifluoromethyl)phenoxy) acetamide (**10r**)

Following general procedure A, compound **10r** (35 mg, yield 33%) was obtained as a white solid. ^1^H NMR (400 MHz, DMSO- *d*_6_) δ 12.29 (s, 1H), 10.30 (s, 1H), 8.29 (d, *J* = 2.0 Hz, 1H), 7.69 (d, *J* = 8.8 Hz, 3H), 7.59 (dd, *J* = 8.8, 2.0 Hz, 1H), 7.20 (d, *J* = 8.8 Hz, 2H), 4.85 (s, 2H), 2.19 (s, 3H). ^13^C NMR (101 MHz, DMSO- *d*_6_) δ 169.75, 166.39, 161.19, 157.70, 145.39, 134.65, 132.34, 127.43 (q, *J* = 360.0 Hz), 124.98 (d, *J* = 269.4 Hz), 122.16 (d, *J* = 31.9 Hz), 120.92, 119.55, 115.70, 112.75, 67.54, 23.19. ^19^F NMR (376 MHz, DMSO- *d*_6_) δ -59.86. HRMS (ESI) [M + H]^+^ calcd for C18H15F3N3O3S: 410.07807; found: 410.07662. R_T_ = 11.814 min, purity = 96.70%. Melting point: 168–170 °C.

#### 4.1.22. *N*-(2-Acetamidobenzo[*d*]thiazol-6-yl)-2-(o-tolyloxy)acetamide (**10s**)

Following general procedure A, compound **10s** (73 mg, yield 55%) was obtained as a white solid. ^1^H NMR (400 MHz, DMSO- *d*_6_) δ 12.29 (s, 1H), 10.18 (s, 1H), 8.31 (d, *J* = 2.0 Hz, 1H), 7.69 (d, *J* = 8.8 Hz, 1H), 7.59 (dd, *J* = 8.8, 2.0 Hz, 1H), 7.21–7.11 (m, 2H), 6.93–6.83 (m, 2H), 4.74 (s, 2H), 2.26 (s, 3H), 2.19 (s, 3H). ^13^C NMR (101 MHz, DMSO- *d*_6_) δ 169.74, 167.12, 157.66, 156.53, 145.34, 134.78, 132.37, 131.09, 127.38, 126.65, 121.38, 120.92, 119.48, 112.63, 111.97, 67.94, 23.19, 16.63. HRMS (ESI) [M + H]^+^ calcd for C18H18N3O3S: 356.10634; found: 356.10494. R_T_ = 11.336 min, purity = 98.76%. Melting point: 164–165 °C.

#### 4.1.23. *N*-(2-Acetamidobenzo[*d*]thiazol-6-yl)-2-(m-tolyloxy)acetamide (**10t**)

Following general procedure A, compound **10t** (59 mg, yield 60%) was obtained as a yellowish solid. ^1^H NMR (400 MHz, DMSO- *d*_6_) δ 12.30 (s, 1H), 10.20 (s, 1H), 8.32 (d, *J* = 2.0 Hz, 1H), 7.69 (d, *J* = 8.8 Hz, 1H), 7.63 (dd, *J* = 8.8, 2.0 Hz, 1H), 7.19 (t, *J* = 8.0 Hz, 1H), 6.89–6.76 (m, 3H), 4.70 (s, 2H), 2.29 (s, 3H), 2.20 (s, 3H). ^13^C NMR (101 MHz, DMSO- *d*_6_) δ 169.75, 167.06, 158.31, 157.66, 145.37, 139.48, 134.74, 132.32, 129.71, 122.44, 120.89, 119.62, 115.93, 112.77, 112.06, 67.62, 23.19, 21.58. HRMS (ESI) [M + H]^+^ calcd for C18H18N3O3S: 356.10634; found: 356.10510. R_T_ = 9.658 min, purity = 99.98%. Melting point: 166–168 °C.

#### 4.1.24. *N*-(2-Acetamidobenzo[*d*]thiazol-6-yl)-2-(p-tolyloxy)acetamide (**10u**)

Following general procedure A, compound **10u** (62 mg, yield 48%) was obtained as a white solid. ^1^H NMR (400 MHz, DMSO- *d*_6_) δ 12.29 (s, 1H), 10.19 (s, 1H), 8.29 (d, *J* = 2.4 Hz, 1H), 7.68 (d, *J* = 8.8 Hz, 1H), 7.60 (dd, *J* = 8.8, 2.4 Hz, 1H), 7.11 (d, *J* = 8.4 Hz, 2H), 6.96–6.84 (m, 2H), 4.67 (s, 2H), 2.23 (s, 3H), 2.19 (s, 3H). ^13^C NMR (101 MHz, DMSO- *d*_6_) δ 169.75, 167.13, 157.64, 156.21, 145.34, 134.74, 132.29, 130.41, 130.31, 120.88, 119.61, 115.03, 112.74, 67.83, 23.20, 20.55. HRMS (ESI) [M + H]^+^ calcd for C18H18N3O3S: 356.10634; found: 356.10507. R_T_ = 11.162 min, purity = 99.98%. Melting point: 163–164 °C.

#### 4.1.25. *N*-(2-Acetamidobenzo[*d*]thiazol-6-yl)-2-(3-methoxyphenoxy)acetamide (**10v**)

Following general procedure A, compound **10v** (62 mg, yield 48%) was obtained as a white solid. ^1^H NMR (400 MHz, DMSO- *d*_6_) δ 12.29 (s, 1H), 10.21 (s, 1H), 8.30 (d, *J* = 2.0 Hz, 1H), 7.68 (d, *J* = 8.8 Hz, 1H), 7.61 (dd, *J* = 8.8, 2.0 Hz, 1H), 7.25–7.17 (m, 1H), 6.68–6.49 (m, 3H), 4.70 (s, 2H), 3.74 (s, 3H), 2.19 (s, 3H). ^13^C NMR (101 MHz, DMSO- *d*_6_) δ 169.75, 166.92, 160.92, 159.48, 157.67, 145.37, 134.70, 132.31, 130.49, 120.89, 119.62, 112.78, 107.36, 107.24, 101.63, 67.73, 55.60, 23.19. HRMS (ESI) [M + H]^+^ calcd for C18H18N3O4S: 372.10125; found: 372.09975. R_T_ = 11.370 min, purity = 99.98%. Melting point: 165–167 °C.

#### 4.1.26. *N*-(2-Acetamidobenzo[*d*]thiazol-6-yl)-2-(4-methoxyphenoxy)acetamide (**10w**)

Following general procedure A, compound **10w** (90 mg, yield 76%) was obtained as a white solid. ^1^H NMR (400 MHz, DMSO- *d*_6_) δ 12.29 (s, 1H), 10.17 (s, 1H), 8.31 (d, *J* = 2.0 Hz, 1H), 7.68 (d, *J* = 8.8 Hz, 1H), 7.62 (dd, *J* = 8.8, 2.0 Hz, 1H), 6.97 (d, *J* = 9.2 Hz, 2H), 6.88 (d, *J* = 9.2 Hz, 2H), 4.64 (s, 2H), 3.69 (s, 3H), 2.19 (s, 3H). ^13^C NMR (101 MHz, DMSO- *d*_6_) δ 169.75, 167.24, 157.64, 154.32, 152.28, 145.35, 134.73, 132.29, 120.87, 119.65, 116.20, 115.07, 112.77, 68.42, 55.82, 23.19. HRMS (ESI) [M + H]^+^ calcd for C18H18N3O4S: 372.10125; found: 372.09988. R_T_ = 10.215 min, purity = 94.20%. Melting point: 165–167 °C.

### 4.2. Viability Assay

Cell viability was assessed using the Cell Counting Kit-8 (CCK-8) assay. Cells were seeded in 96-well plates at a density of 10,000 cells per well in RPMI 1640 medium. Test compounds were prepared by serially diluting stock solutions 3-fold in DMSO to generate an eight-point concentration gradient (100 μM to 0.045 μM), followed by addition to the cultured cells. After 72 h of incubation, 10 μL of CCK-8 reagent was added to each well, and the plates were further incubated for 2–4 h. Absorbance was measured at 450 nm using a microplate reader. Dose-response curves were generated and analyzed using GraphPad Prism 8 (version 8.4.3.686).

### 4.3. Apoptosis Assays

Cell apoptosis was evaluated through Annexin V and propidium iodide (PI) dual staining using an Annexin V-FITC Apoptosis Detection Kit (Vazyme, #A211-02, Nanjing, China) following the manufacturer’s protocol. Cells were seeded at a density of 500,000 cells per well in 12-well plates containing RPMI 1640 medium. K562 cells were treated with either compound **10m** or asciminib for 48 or 72 h. Apoptotic signals were quantified using a FACS Calibur flow cytometer (Cy-toFLEX).

### 4.4. Western Blot Analysis

Western blot analysis was conducted following established protocols. K562 cells were treated with varying concentrations of the test compound for 60 min, followed by protein extraction using RIPA buffer. Protein quantification was performed using bicinchoninic acid assay, and 30–60 μg of protein samples were resolved by 10% sodium dodecyl sulfate-polyacrylamide gel electrophoresis before being transferred onto nitrocellulose membranes. Membranes were probed with primary antibodies overnight at 4 °C. Following incubation with horseradish peroxidase (HRP)-conjugated secondary antibodies (Thermo Scientific, Waltham, MA, USA), protein bands were detected using a chemiluminescent HRP substrate (Millipore Corporation, #WBKLS0500, Billerica, MA, USA) and imaged with a Syngene GeneGnome-XRQ-NPC imager (Synoptics, Britain). Quantitative analysis was performed by measuring band intensity using ImageJ (version 1.43) software. The following primary antibodies were used: c-Abl (ABclonal, #A0282, Woburn, MA, USA), phosphor-c-Abl (ABclonal, #AP0001), STAT5 (ABclonal, #A5029), phosphor-STAT5 (ABclonal, #AP0887), CRKL (ABclonal, #A11735), and phospho-CRKL (ABclonal, #AP0824).

### 4.5. Molecular Docking and MD Simulations

The protein structure was retrieved from the Protein Data Bank (http://www.rcsb.org/pdb, accessed on 10 December 2024). The X-ray crystal structure of BCR-ABL (PDB id: 8SSN) was selected for molecular docking studies using Maestro (Schrödinger, LLC, New York, NY, USA, 2019). Ligand docking was performed in the orthosteric binding pocket using Glide. Based on the docking results, separate 50 ns molecular dynamics simulations were conducted with Desmond. To maintain system neutrality, Na^+^ and Cl^−^ ions were added at physiological concentration (0.15 mol·L^−1^). Simulations utilized the OPLS3 force field and TIP3P explicit solvent model, resulting in a solvated system containing approximately ~37,000 atoms. Data were recorded at 20 ps intervals under NPT ensemble conditions, with temperature maintained at 300 K and pressure at 1.01 bar. Ligand-protein interactions were analyzed using the Simulation Interactions Diagram tool. For visual inspection, MD simulations were employed as the final step in the virtual screening pipeline. After the ChemDiv and Specs commercial compound libraries were processed through Glide SP and XP docking, a 20 ns MD simulation was used to filter and identify the final candidate compounds, with the evaluation criteria including: stable RMSD values of the heavy atoms of the small molecules relative to the backbone atoms of the kinase throughout the simulation (<2 Å), sustained hydrogen bond interactions with Met-337, and prolonged π-π interactions with Phe-401.

### 4.6. Synergy Study

SynergyFinder 3.0 (https://synergyfinder.fimm.fi, accessed on 10 December 2024) is a web application for interactive analysis and visualization of multi-drug combination response data. Cells were plated at a density of 10,000 cells per well in 96-well plates containing RPMI 1640 medium. Imatinib or compound **10m** was administered in combination with asciminib, with the concentrations of test compounds added as specified in Figure 4B,C. Following a 72 h incubation period, 10 μL of CCK-8 dye was added to each well, and the cells were further incubated for 2–4 h. Fluorescence intensity was subsequently measured at 450 nm. The drug combination effects were quantified as percentage inhibition of cell viability. The experimental data were analyzed using SynergyFinder 3.0, with a four-parameter logistic regression (LL4) model selected for curve fitting and data analysis. The Synergy Score based on the ZIP (Zero Interaction Potency) model is used to evaluate the synergistic effects of drug combinations.

### 4.7. Reagents and Compounds

Compounds used for BCR-ABL1 inhibitors screening (**A1**–**A15**, see Table S1) were purchased from the ChemDiv (https://www.chemdiv.com, accessed on 10 December 2024) with a purity of more than 95%. Imatinib, ponatinib, and asciminib were purchased from Bidepharm Co., Ltd. (Shanghai, China).

## 5. Conclusions

In conclusion, the combination of orthosteric and allosteric BCR-ABL1 inhibitors holds great promise for overcoming resistance in CML and ALL treatment. Structure-based virtual screening and SAR studies led to the identification of compound **A8**, featuring a novel 2-acetamidobenzo[*d*]thiazol scaffold. Derivatives of **A8**, particularly compound **10m**, demonstrated potent anti-proliferative activity, inhibited BCR-ABL signaling, and exhibited synergistic effects with asciminib in cell growth and apoptosis. These findings highlight compound **10m** as a potential lead for developing dual-targeting BCR-ABL1 therapeutic agents.

## Figures and Tables

**Table 1:** Antiproliferative activity of compounds **A8**, **A12**–**A15** ^a^.

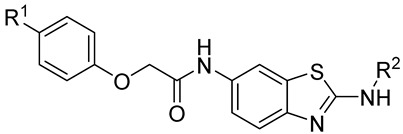				
Compound No.	R^1^	R^2^	Ba/F3 GI_50_ (μM)	
			BCR-ABL1	Parental Cells
**A8**	Cl	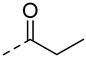	6.4 ± 1.6	>100
**A12**	H	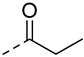	10.7 ± 2.6	>100
**A13**	H	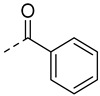	19.5 ± 4.7	>100
**A14**	H	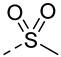	1.2 ± 0.2	37.6 ± 5.7
**A15**	H	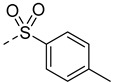	3.9 ± 0.5	42.9 ± 10.4
Imatinib	/	/	0.133 ± 0.005	38.8 ± 4.5
Asciminib	/	/	0.134 ± 0.001	>100

^a^ Cellular assay was determined using CCK8 kit. The data were shown as mean ± SD from at least three independent experiments.

**Table 2:** Antiproliferative activity of compounds **10a**–**w**.

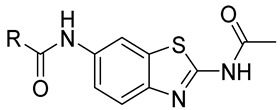		
Compound No.	R	Ba/F3 (BCR-ABL1) Inhibition (%) ^a^
**10a**	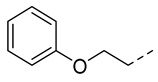	52 ± 5
**10b**	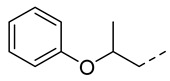	NA ^b^
**10c**	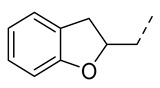	NA
**10d**	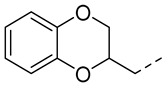	NA
**10e**	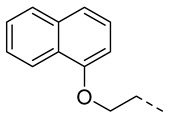	NA
**10f**	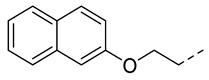	88 ± 2
**10g**	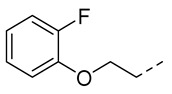	65 ± 3
**10h**	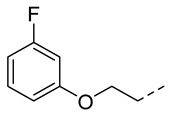	61 ± 5
**10i**	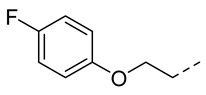	58 ± 2
**10j**	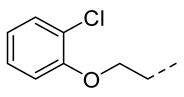	68 ± 2
**10k**	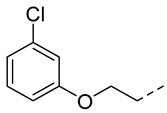	60 ± 6
**10l**	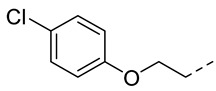	71 ± 4
**10m**	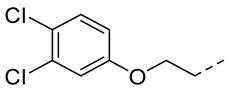	93 ± 5
**10n**	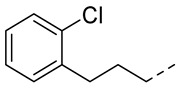	NA
**10o**	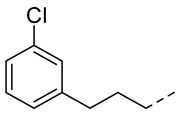	NA
**10p**	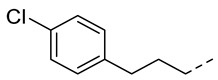	NA
**10q**	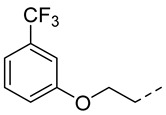	75 ± 6
**10r**	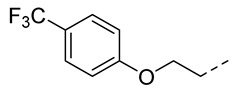	36 ± 3
**10s**	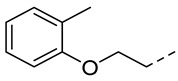	22 ± 2
**10t**	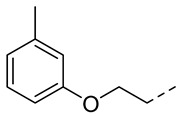	28 ± 5
**10u**	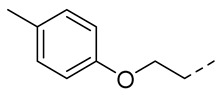	NA
**10v**	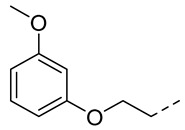	NA
**10w**	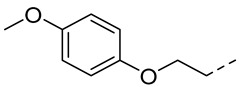	NA

^a^ The inhibition of Ba/F3 (BCR-ABL1) cells of each compound was measured at 10 μM. The data were shown as mean ± SD from at least three independent experiments. ^b^ NA: no activity.

**Table 3:** Antiproliferative activity of selected compounds in leukemia cell lines ^a^.

Compound	Ba/F3			MV411	HL60
	BCR-ABL1	BCR-ABL^T315I^	Parental		
**10f**	2.2 ± 0.5	>100	>100	>100	>100
**10m**	0.63 ± 0.1	25.2 ± 1.1	>100	>100	54.2 ± 5.5
Imatinib	0.133 ± 0.005	>100	38.8 ± 4.5	65.7 ± 6.5	29.1 ± 3.3
Asciminib	0.134 ± 0.001	0.21	>100	>100	>100

^a^ IC_50_ on cells, compound concentration required to inhibit tumor cell proliferation by 50%. The data were shown as mean ± SD from at least three independent experiments.

## Data Availability

The original contributions presented in this study are included in the article/supplementary material. Further inquiries can be directed to the corresponding author.

## Supplementary Materials

The following supporting information can be downloaded at: https://www.mdpi.com/article/10.3390/molecules30051065/s1, Figure S1: Structure and Ba/F3 (BCR-ABL1) inhibitory activity of compounds **A1**–**A11**. Figure S2: The predicted binding modes of **10f** (left) and **10m** (right) with the BCR-ABL crystal structure. Figure S3: Unprocessed gels and Western blot for Figure 5. Figure S4: MD simulations of compound **10m**. Table S1: The information for compounds **A1**–**A15**. Table S2: ZIP synergy scores of imatinib in combination with asciminib. Table S3: ZIP synergy scores of **10m** in combination with asciminib. ^1^H and ^13^C NMR spectra of new compounds. HPLC purity of new compounds.

## Abbreviations

The following abbreviations are used in this manuscript:

MD	Molecular dynamics
RMSD	Root mean square deviations
SAR	Structure activity relationship
SP	Standard precision
XP	Extra precision
2D	Two-dimensional
TKIs	Tyrosine kinase inhibitors
