# Supplementary material for: Discovery of N-(2-Acetamidobenzo[d]thiazol-6-yl)-2-phenoxyacetamide Derivatives as Novel Potential BCR-ABL1 Inhibitors Through Structure-Based Virtual Screening
Source: Molecules. 2025 Feb 26;30(5):1065. doi: 10.3390/molecules30051065 (PMC11901765; doi:10.3390/molecules30051065)
Supplement: Supplementary file 1 [file molecules-30-01065-s001.zip › molecules-3443092-supplementary.pdf]

Discovery of *N*-(2-acetamidobenzo[*d*]thiazol-6-yl)-2-phenoxyacetamide derivatives as novel potential BCR-ABL inhibitors through structure-based virtual screening.

Shuaixing Wang [1], Minyi Wang [1], Zi Li [1], Guofeng Xu [2] and Dayan Wang [1,*]

[1] National Institute for Viral Disease Control and Prevention, Chinese Center for Disease Control and Prevention; WHO Collaborating Centre for Reference and Research on Influenza; Key Laboratory for Medical Virology and Viral Diseases, National Health Commission; National Key Laboratory of Intelligent Tracking and Forecasting for Infectious Disease, Beijing, People's Republic of China.

[2] State Key Laboratory of Natural and Biomimetic Drugs, School of Pharmaceutical Sciences, Peking University, Beijing, China.

[*] Correspondence: E-mail: wangdayan@ivdc.chinacdc.cn; Tel.: +860158900850; Address for correspondence: National Institute for Viral Disease Control and Prevention, China CDC. 155 Changbai Rd, Changping District, Beijing, 102206, China.

1. **Figure S1** Structure and Ba/F3 (BCR-ABL) inhibitory activity of compounds **A1** – **A11**.
2. **Figure** S2 The predicted binding mode of compound **A8**
3. **Figure S3** The predicted binding modes of **10f** (left) and **10m** (right) with the BCR-ABL crystal structure.
4. **Figure S4** MD simulations of compound **10m**.
5. **Figure** S5 Unprocessed gels and western blots for **Figure 5.**
6. **Table S1.** The information for compounds **A1** – **A15.**
7. **Table S2** ZIP synergy scores of imatinib in combination with asciminib.
8. **Table S2** ZIP synergy scores of **10m** in combination with asciminib.
9. ^{1}H and ^{13}C NMR spectra of new compounds.
10. HRMS data of new compounds
11. HPLC purity of new compounds.

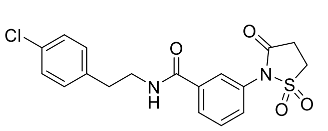

A1 (D473-1567)
BaF3 (BCR-ABL) GI_{50} > 50 μM

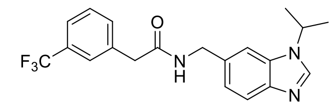

A2 (M414-0425)
BaF3 (BCR-ABL) GI_{50} = 46.5 μM

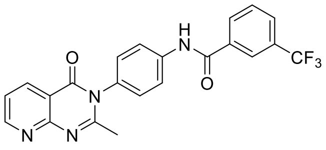

A3 (F306-0023)
BaF3 (BCR-ABL) GI_{50} > 50 μM

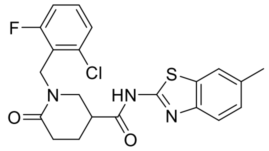

A4 (G843-0521)
BaF3 (BCR-ABL) GI_{50} = 33.1 μM

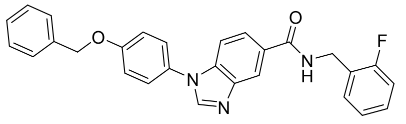

A5 (K783-0026)
BaF3 (BCR-ABL) GI_{50} = 20.3 μM

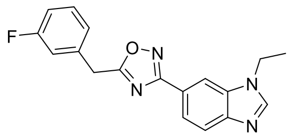

A6 (P597-0250)
BaF3 (BCR-ABL) GI_{50} > 50 μM

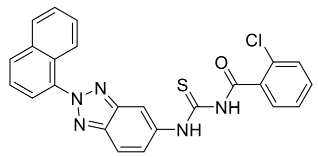

A7 (3149-0193)
BaF3 (BCR-ABL) GI_{50} > 50 μM

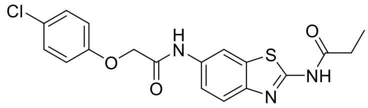

A8 (F259-0208)
BaF3 (BCR-ABL) GI_{50} = 5.9 μM

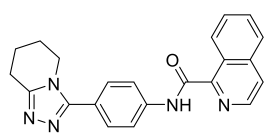

A9 (S346-0051)
BaF3 (BCR-ABL) GI_{50} > 50 μM

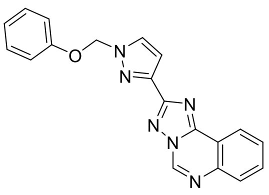

A10 (Y502-8023)
BaF3 (BCR-ABL) GI_{50} > 50 μM

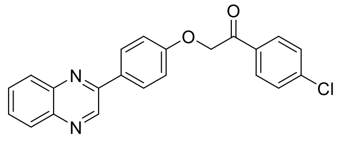

A11 (6420-3611)
BaF3 (BCR-ABL) GI_{50} = 15.9 μM

Figure S1 Structure and Ba/F3 (BCR-ABL) inhibitory activity of compounds **A1** – **A11**.

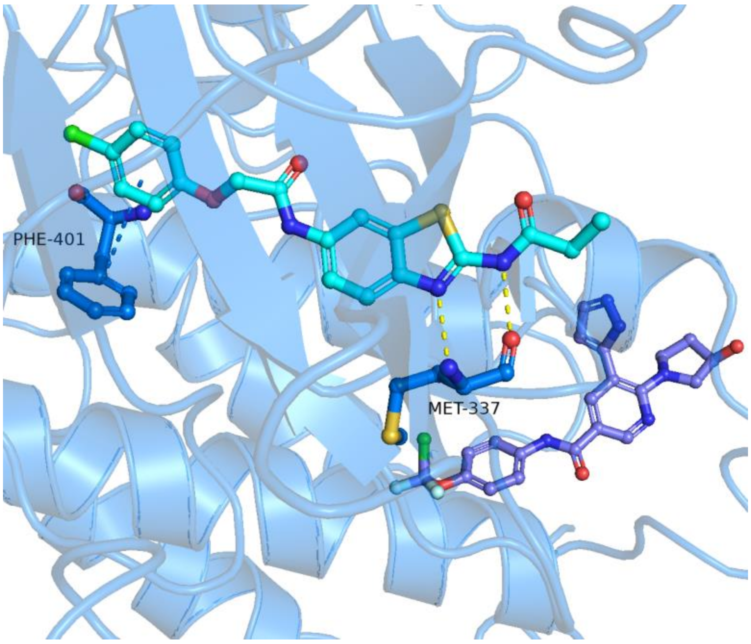

Figure S2 The predicted binding modes of hit compound **A8** (cyan) with the BCR-ABL crystal structure (PDB id: 8SSN). Asciminib was indicated by lilac lines. Hydrogen bonds were indicated by yellow dash lines. π-π interaction was indicated by blue dash lines.

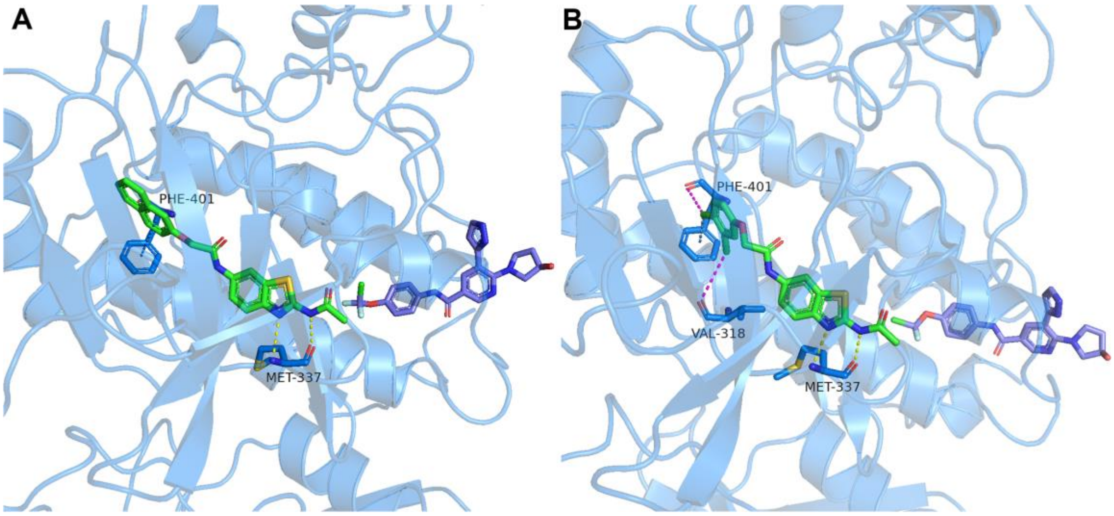

Figure S3 The predicted binding modes of **10f** (left) and **10m** (right) with the BCR-ABL crystal structure (PDB id: 8SSN). Compounds **10f**, **10m** were indicated by green lines, and asciminib was indicated by lilac lines. Hydrogen bonds were indicated by yellow dash lines. π-π interaction was indicated by blue dash lines. Halogen bonds were indicated by magenta dash lines.

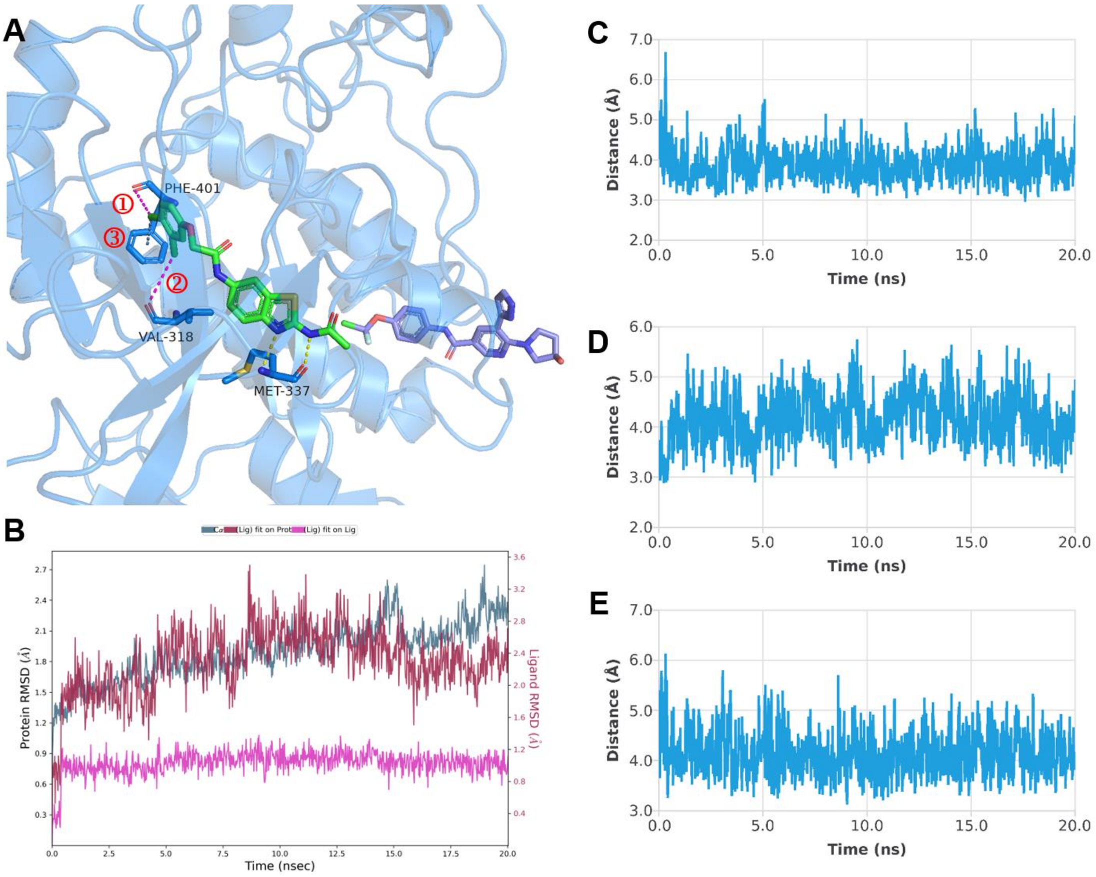

Figure S4 MD simulations of compound **10m**. (A) The predicted binding mode of compound 10m. The distance between the chlorine atom at the 4-position of **10m**'s benzene ring and the carbonyl oxygen atom of Phe-401 is indicated by label ①, with the corresponding distance variation plotted in Panel C. The distance between the chlorine atom at the 4-position of **10m**'s benzene ring and the plane of the Phe-401 benzene ring is indicated by label ②, with the corresponding distance variation shown in Panel D. The distance between the chlorine atom at the 3-position of **10m**'s benzene ring and the carbonyl oxygen atom of Val-318 is indicated by label ③, with the corresponding distance variation presented in Panel E. (B) RMSD of protein and ligand **10m** through MD simulations. (C-E) The distance variations are plotted against the simulation time (0-20 ns).

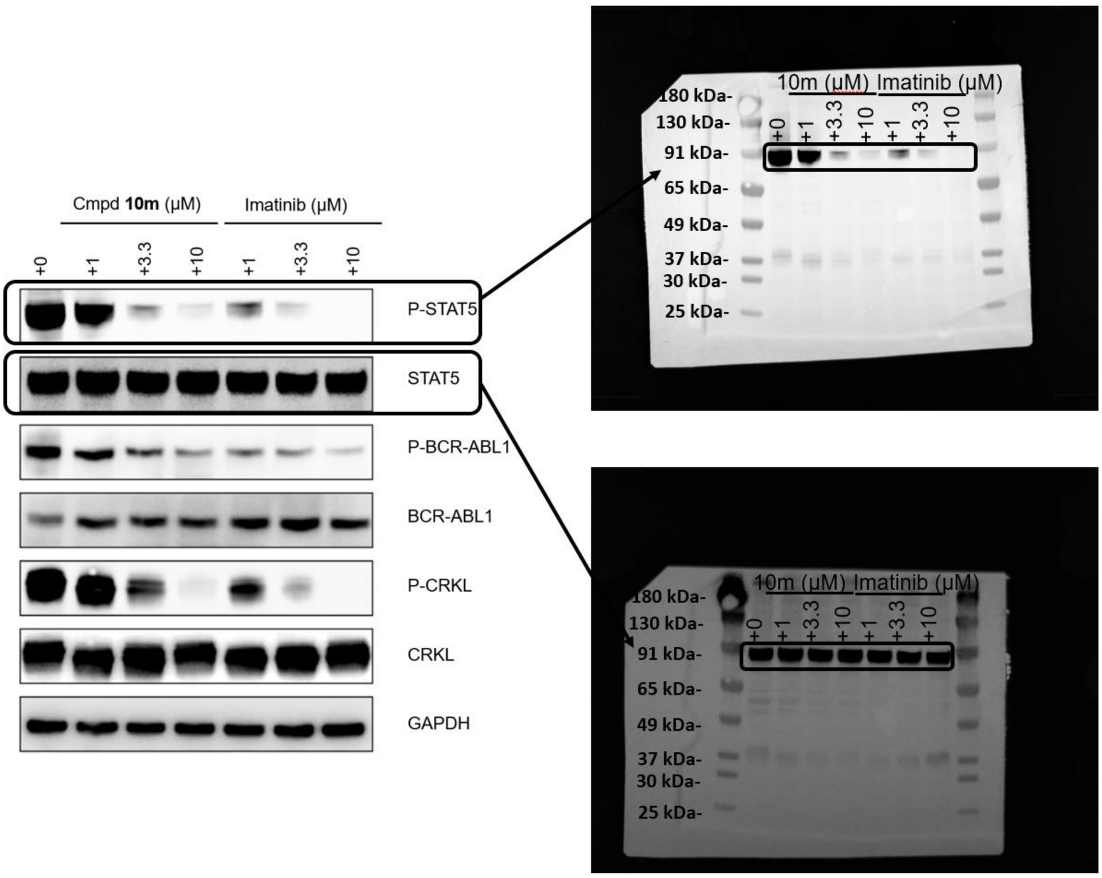
Cmpd 10m (μM)
Imatinib (μM)
+0
+1
+3.3
+10
+1
+3.3
+10
P-STAT5
STAT5
P-BCR-ABL1
BCR-ABL1
P-CRKL
CRKL
GAPDH
10m (μM)
Imatinib (μM)
180 kDa-
130 kDa-
91 kDa-
65 kDa-
49 kDa-
37 kDa-
30 kDa-
25 kDa-

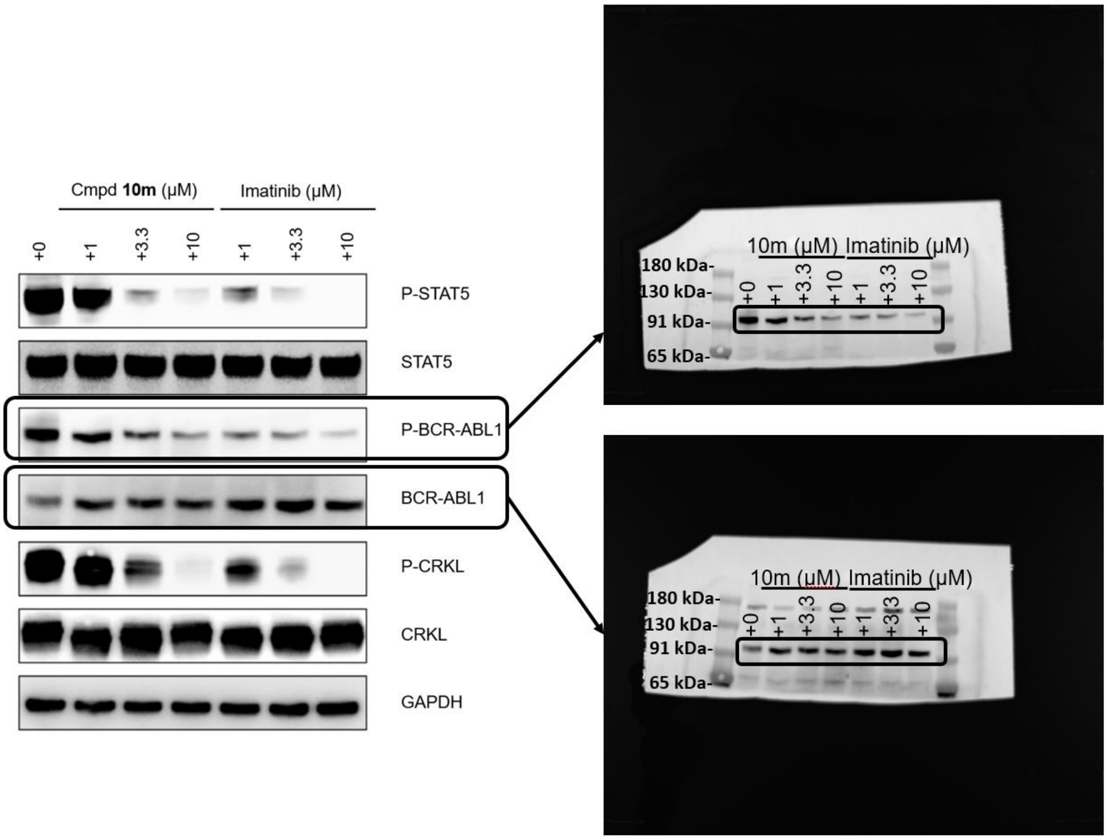
Cmpd 10m (μM)
Imatinib (μM)
+0
+1
+3.3
+10
+1
+3.3
+10
P-STAT5
STAT5
P-BCR-ABL1
BCR-ABL1
P-CRKL
CRKL
GAPDH
10m (μM)
Imatinib (μM)
180 kDa-
130 kDa-
91 kDa-
65 kDa-

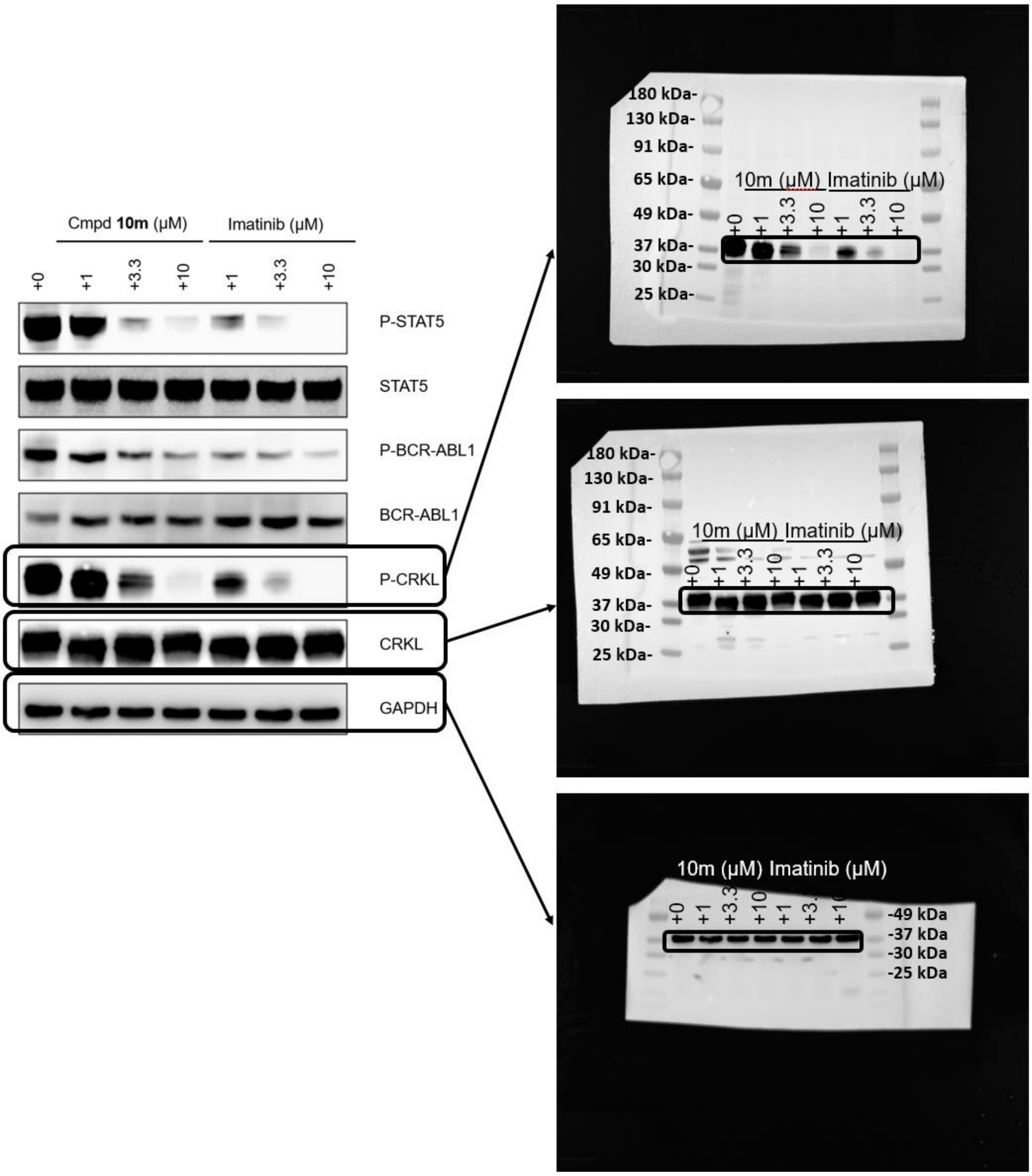

Figure S5 Unprocessed gels and western blots for **Figure 5**.

Table S1. The information for compounds A1 – A15.

Compound No.	ChemDiv ID	Docking score	Smiles
**A1**	D473-1567	-12.075	C1CS(N(C1=O)c1cccc(c1)C(NCCc1ccc(cc1)[Cl])=O)(=O)=O
**A2**	M414-0425	-13.35	CC(C)n1cnc2cc(CNC(Cc3cccc(c3)C(F)(F)F)=O)ccc12
**A3**	F306-0023	-12.231	CC1=Nc2c(cccn2)C(N1c1ccc(cc1)NC(c1cccc(c1)C(F)(F)F)=O)=O
**A4**	G843-0521	-12.194	Cc1ccc2c(c1)sc(NC(C1C=CC(N(Cc3c(cccc3[Cl])F)C=1)=O)=O)n2
**A5**	K783-0026	-11.869	C(c1ccccc1[Cl])NC(c1ccc2c(c1)ncn2c1ccc(cc1)OCc1ccccc1)=O
**A6**	P597-0250	-11.813	CCn1cnc2ccc(cc12)c1nc(Cc2cccc(c2)F)on1
**A7**	3149-0193	-12.599	c1ccc(c(c1)C(NC(Nc1ccc2c(c1)nn(c1cccc3ccccc13)n2)=S)=O)[Cl]
**A8**	F259-0208	-12.098	CCC(Nc1nc2ccc(cc2s1)NC(COc1ccc(cc1)[Cl])=O)=O
**A9**	S346-0051	-11.664	C1CCn2c(C1)nnc2c1ccc(cc1)NC(c1c2ccccc2ccn1)=O
**A10**	Y502-8023	-11.961	C(n1ccc(c2nc3c4ccccc4ncn3n2)n1)Oc1ccccc1
**A11**	6420-3611	-11.824	C(C(c1ccc(cc1)[Cl])=O)Oc1ccc(cc1)c1cnc2ccccc2n1
**A12**	F259-0336	/	CCC(Nc1nc2ccc(cc2s1)NC(COc1ccccc1)=O)=O
**A13**	F259-0092	/	C(C(Nc1ccc2c(c1)sc(NC(c1ccccc1)=O)n2)=O)Oc1ccccc1
**A14**	F260-0283	/	CS(Nc1nc2ccc(cc2s1)NC(COc1ccccc1)=O)(=O)=O
**A15**	F260-0069	/	Cc1ccc(cc1)S(Nc1nc2ccc(cc2s1)NC(COc1ccccc1)=O)(=O)=O

Table S2 ZIP synergy scores of imatinib in combination with asciminib.

PairIndex	Drug1	Drug2	Conc1	Conc2	Relative inhibition	Synergy	Conc
1	Asciminib	Imatinib	0.12	0.04	76.65	0	uM
1	Asciminib	Imatinib	0.37	0.04	73.54	0	uM
1	Asciminib	Imatinib	1.11	0.04	77.63	0	uM
1	Asciminib	Imatinib	3.33	0.04	74.44	0	uM
1	Asciminib	Imatinib	10	0.04	76.63	0	uM
1	Asciminib	Imatinib	30	0.04	90.31	0	uM
1	Asciminib	Imatinib	0.12	0.12	76.31	0	uM
1	Asciminib	Imatinib	0.37	0.12	75.39	-23.098	uM
1	Asciminib	Imatinib	1.11	0.12	79.23	-21.6955	uM
1	Asciminib	Imatinib	3.33	0.12	72.89	-24.3152	uM
1	Asciminib	Imatinib	10	0.12	81.17	-15.8493	uM
1	Asciminib	Imatinib	30	0.12	91.6	-5.68646	uM
1	Asciminib	Imatinib	0.12	0.37	79.95	0	uM
1	Asciminib	Imatinib	0.37	0.37	75.05	-22.9222	uM
1	Asciminib	Imatinib	1.11	0.37	78.92	-21.5528	uM
1	Asciminib	Imatinib	3.33	0.37	71.45	-24.2971	uM
1	Asciminib	Imatinib	10	0.37	85.08	-11.9345	uM
1	Asciminib	Imatinib	30	0.37	97.18	-0.62277	uM
1	Asciminib	Imatinib	0.12	1.11	78.65	0	uM
1	Asciminib	Imatinib	0.37	1.11	80.24	-22.2664	uM
1	Asciminib	Imatinib	1.11	1.11	77.32	-21.074	uM
1	Asciminib	Imatinib	3.33	1.11	72.09	-24.2219	uM
1	Asciminib	Imatinib	10	1.11	85.19	-10.4134	uM
1	Asciminib	Imatinib	30	1.11	95.88	-1.41975	uM
1	Asciminib	Imatinib	0.12	3.33	81.32	0	uM
1	Asciminib	Imatinib	0.37	3.33	80.5	-18.8994	uM
1	Asciminib	Imatinib	1.11	3.33	80.5	-19.1775	uM
1	Asciminib	Imatinib	3.33	3.33	77.26	-22.1068	uM
1	Asciminib	Imatinib	10	3.33	92.46	0.193185	uM
1	Asciminib	Imatinib	30	3.33	96.43	-0.2349	uM
1	Asciminib	Imatinib	0.12	10	90.97	0	uM
1	Asciminib	Imatinib	0.37	10	93.27	-6.63041	uM
1	Asciminib	Imatinib	1.11	10	93.16	-6.5946	uM
1	Asciminib	Imatinib	3.33	10	90.74	-7.84591	uM
1	Asciminib	Imatinib	10	10	99.22	4.677652	uM
1	Asciminib	Imatinib	30	10	99.28	1.751669	uM

Table S3 ZIP synergy scores of **10m** in combination with asciminib.

PairIndex	Drug1	Drug2	Conc1	Conc2	Relative inhibition	Synergy	Conc
2	Asciminib	10m	0.12	0.14	22.87	0	uM
2	Asciminib	10m	0.37	0.14	33.24	0	uM
2	Asciminib	10m	1.11	0.14	63.33	0	uM
2	Asciminib	10m	3.33	0.14	92.94	0	uM
2	Asciminib	10m	10	0.14	95.4	0	uM
2	Asciminib	10m	30	0.14	91	0	uM
2	Asciminib	10m	0.12	0.41	32.31	0	uM
2	Asciminib	10m	0.37	0.41	35.14	-19.2741	uM
2	Asciminib	10m	1.11	0.41	66.39	-16.0181	uM
2	Asciminib	10m	3.33	0.41	92.37	-3.81115	uM
2	Asciminib	10m	10	0.41	95.75	-2.54178	uM
2	Asciminib	10m	30	0.41	90.6	-3.84222	uM
2	Asciminib	10m	0.12	1.23	54.73	0	uM
2	Asciminib	10m	0.37	1.23	39.66	-18.7295	uM
2	Asciminib	10m	1.11	1.23	66.13	-15.9966	uM
2	Asciminib	10m	3.33	1.23	92.62	-3.77162	uM
2	Asciminib	10m	10	1.23	95.19	-2.47692	uM
2	Asciminib	10m	30	1.23	91.09	-3.8029	uM
2	Asciminib	10m	0.12	3.7	77.83	0	uM
2	Asciminib	10m	0.37	3.7	40.79	-16.3473	uM
2	Asciminib	10m	1.11	3.7	66.73	-14.9019	uM
2	Asciminib	10m	3.33	3.7	92.77	-3.21498	uM
2	Asciminib	10m	10	3.7	96.13	-1.85086	uM
2	Asciminib	10m	30	3.7	92.5	-3.25664	uM
2	Asciminib	10m	0.12	11.11	28.6	0	uM
2	Asciminib	10m	0.37	11.11	63.06	32.19233	uM
2	Asciminib	10m	1.11	11.11	72.69	10.01547	uM
2	Asciminib	10m	3.33	11.11	95.03	0.137512	uM
2	Asciminib	10m	10	11.11	94.29	2.330703	uM
2	Asciminib	10m	30	11.11	94.27	1.359448	uM
2	Asciminib	10m	0.12	33.33	29.42	0	uM
2	Asciminib	10m	0.37	33.33	82.34	58.38169	uM
2	Asciminib	10m	1.11	33.33	93.13	35.66925	uM
2	Asciminib	10m	3.33	33.33	94.09	3.004526	uM
2	Asciminib	10m	10	33.33	97.69	4.619397	uM
2	Asciminib	10m	30	33.33	98.83	5.517655	uM

^{1}H NMR of compound **7** (400 MHz, DMSO-d_6)

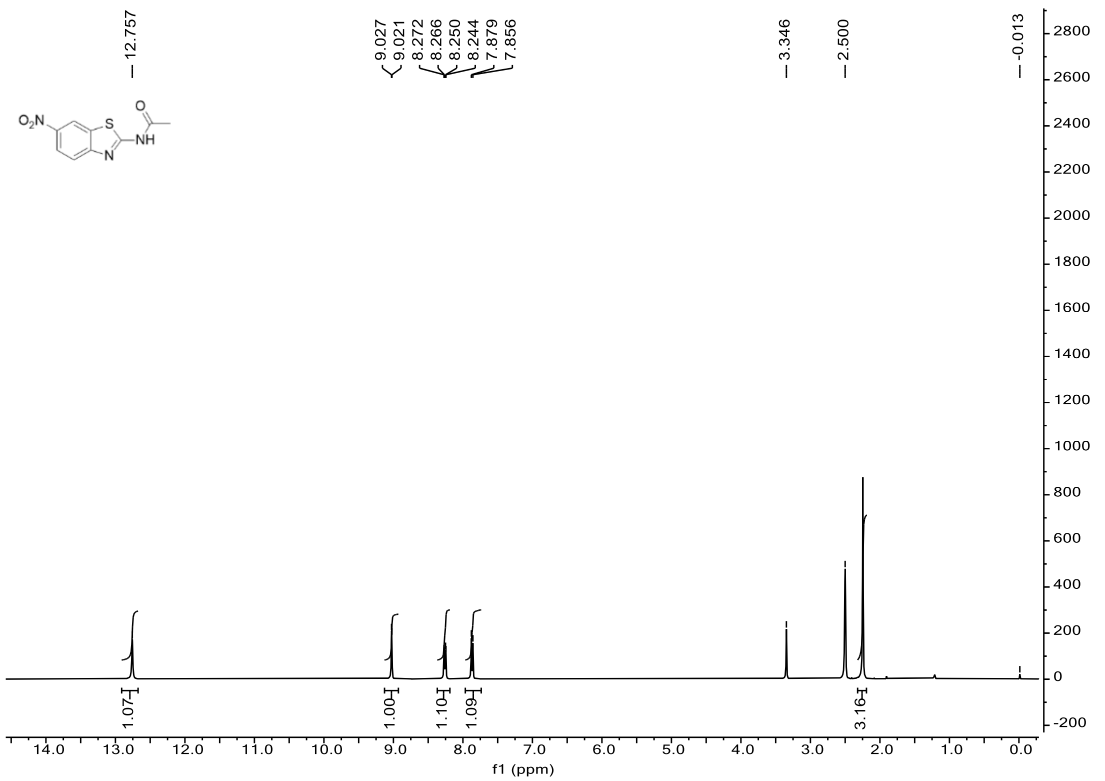

^{13}C NMR of compound **7** (100 MHz, DMSO-d_6)

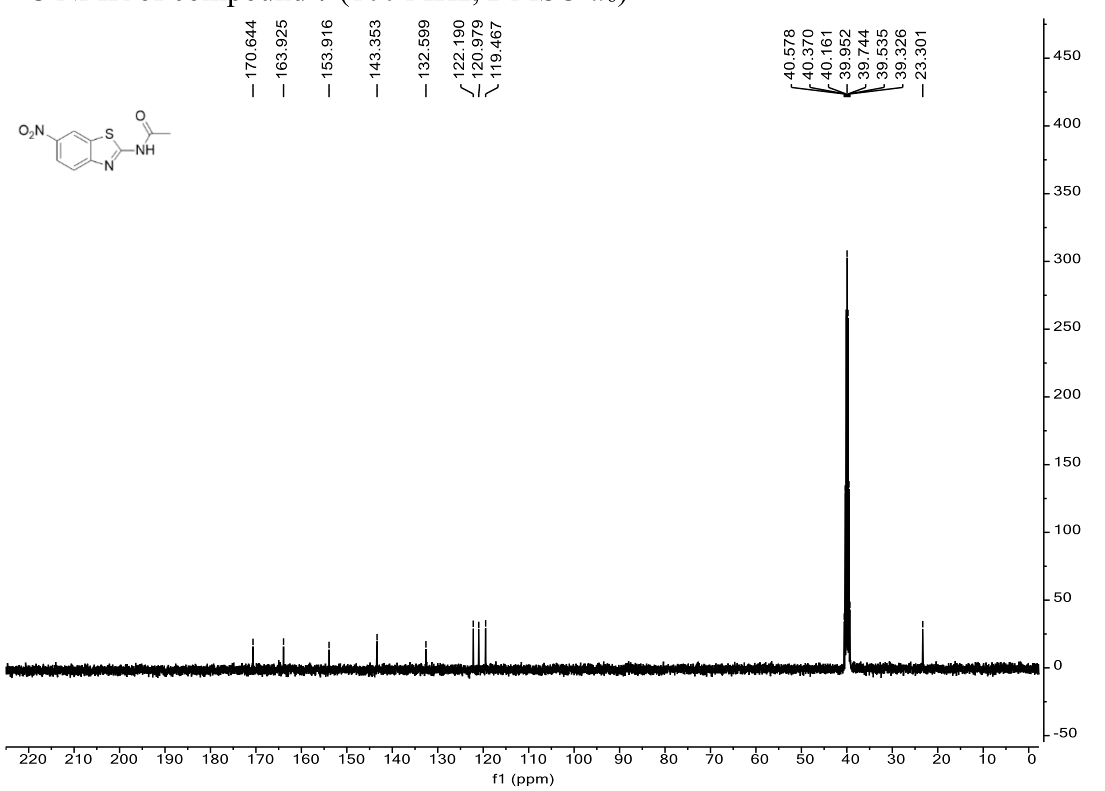

^{1}H NMR of compound **9** (400 MHz, DMSO-d_6)

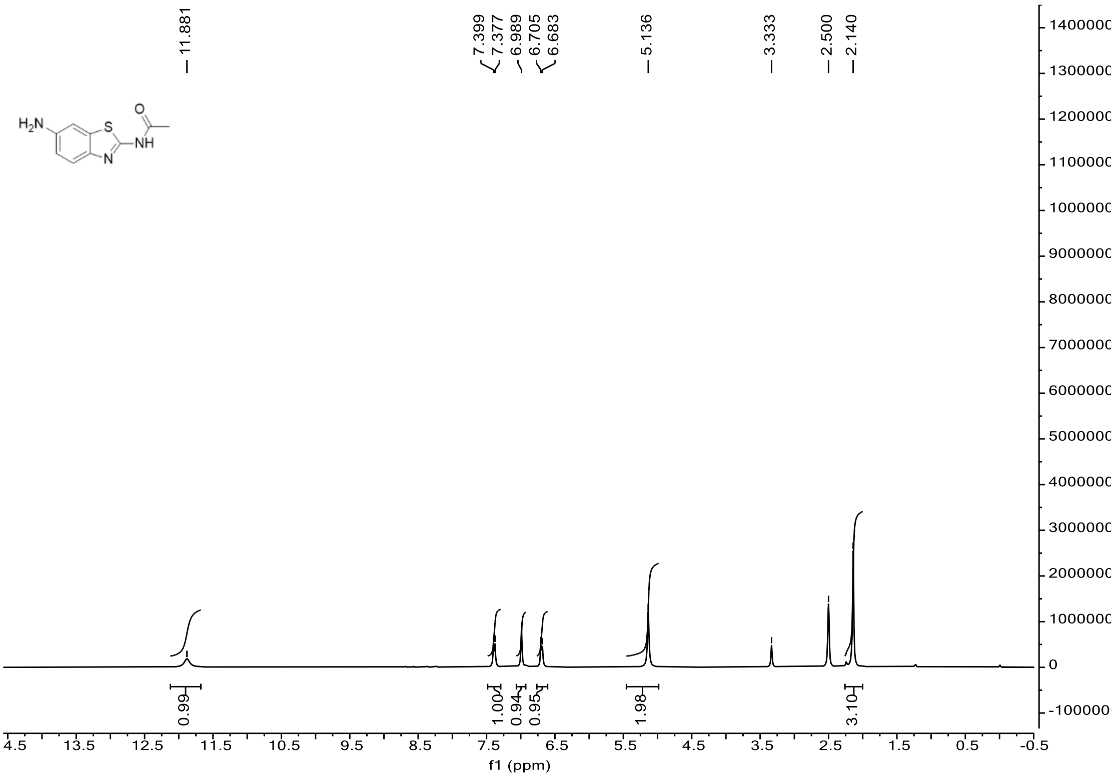

^{13}C NMR of compound **9** (100 MHz, DMSO-d_6)

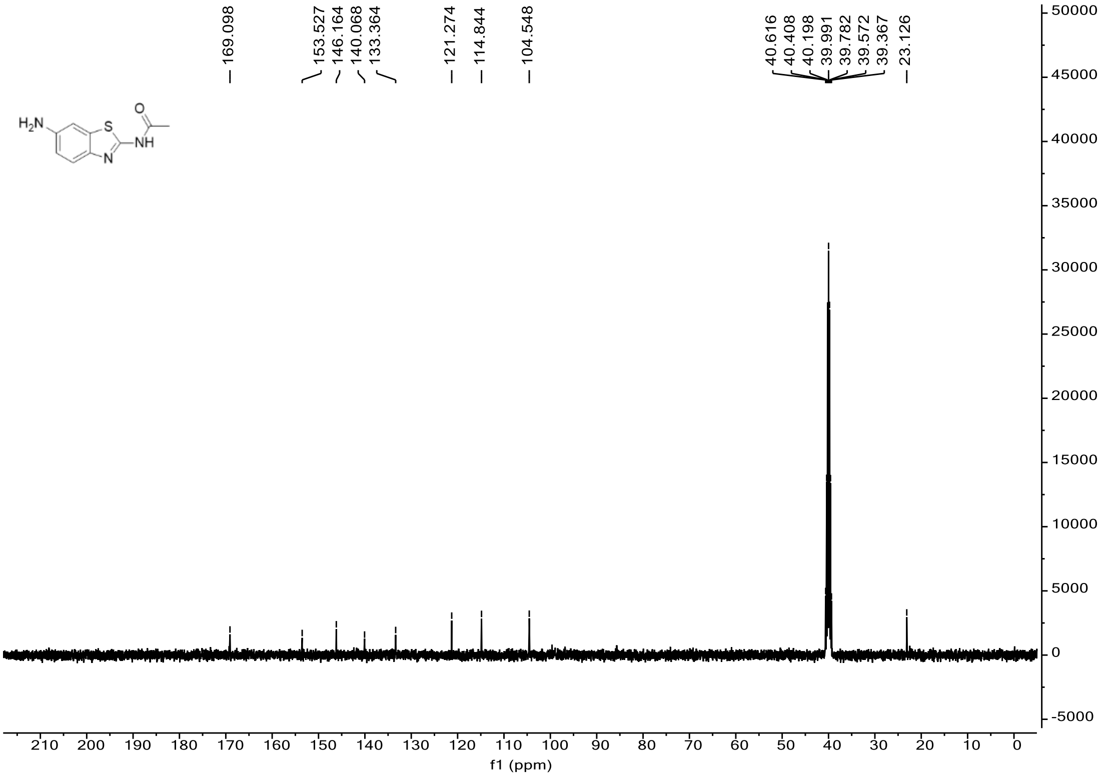

^{1}H NMR of compound **10a** (400 MHz, DMSO-d_6)

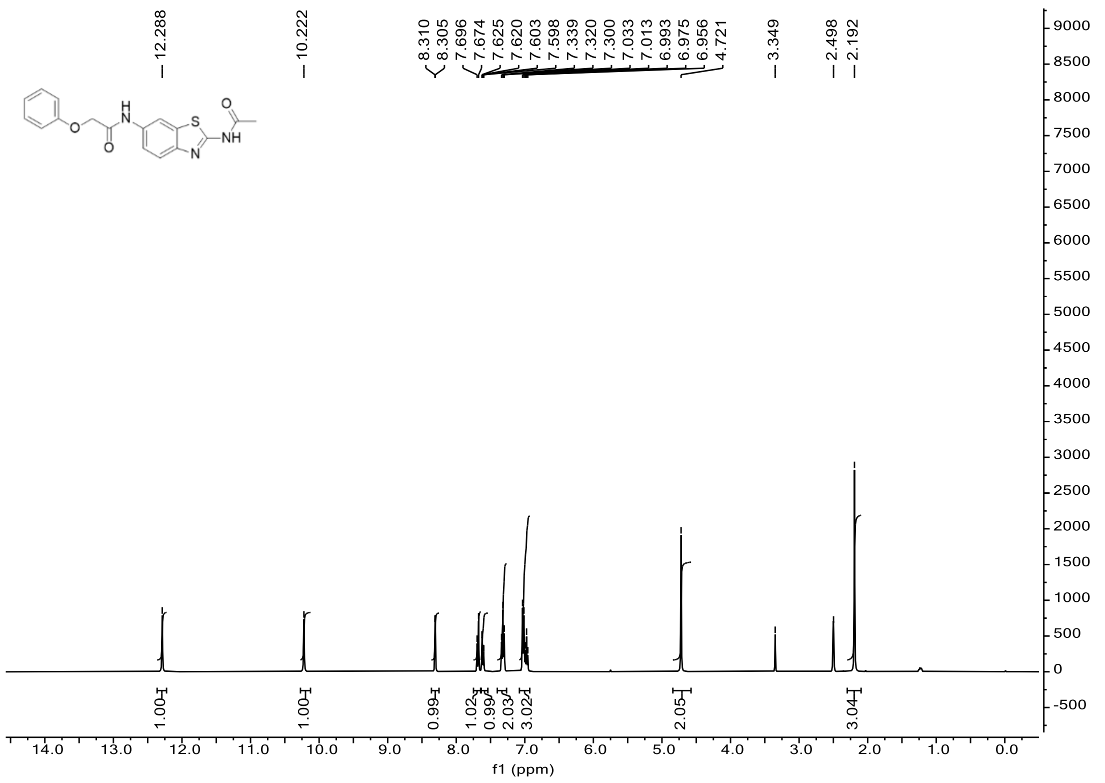

^{13}C NMR of compound **10a** (100 MHz, DMSO-d_6)

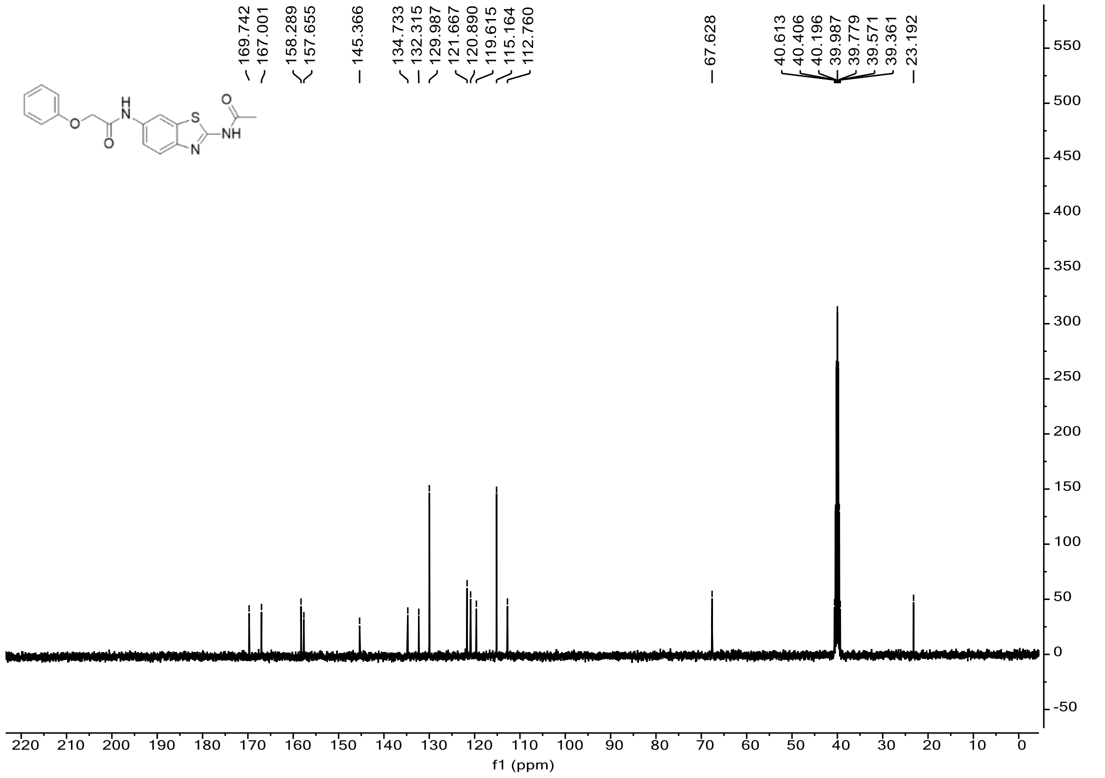

^{1}H NMR of compound **10b** (400 MHz, DMSO-d_6)

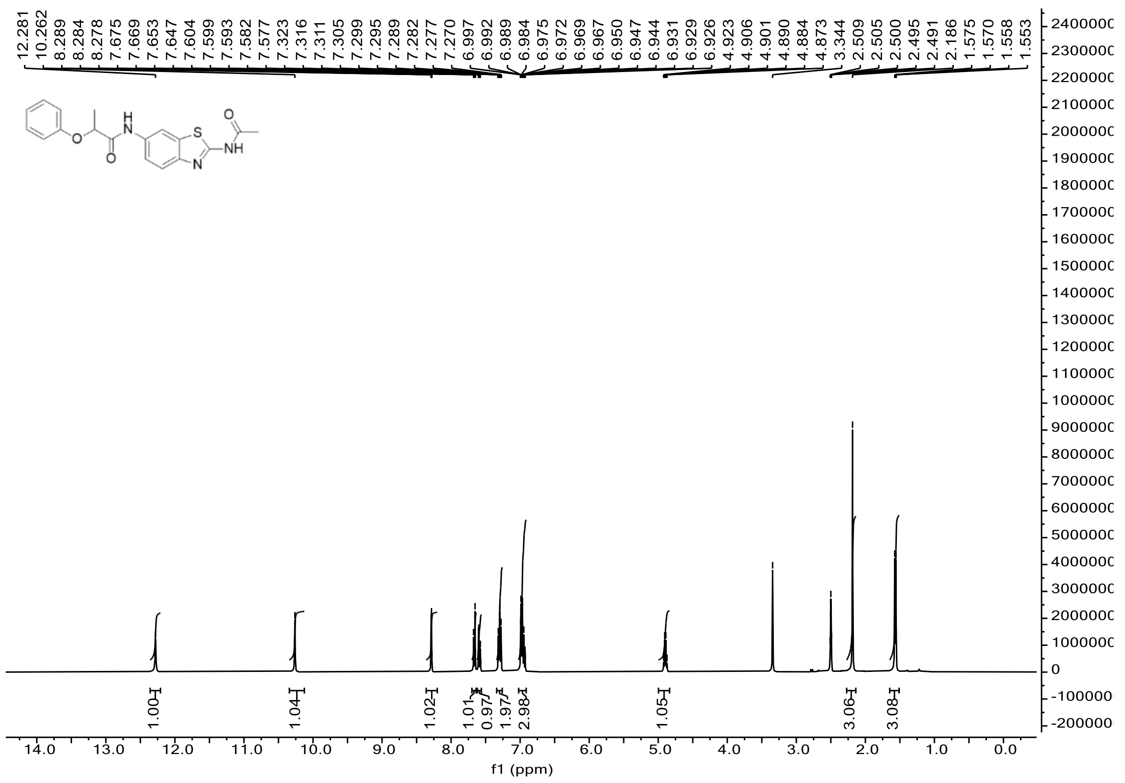

^{13}C NMR of compound **10b** (100 MHz, DMSO-d_6)

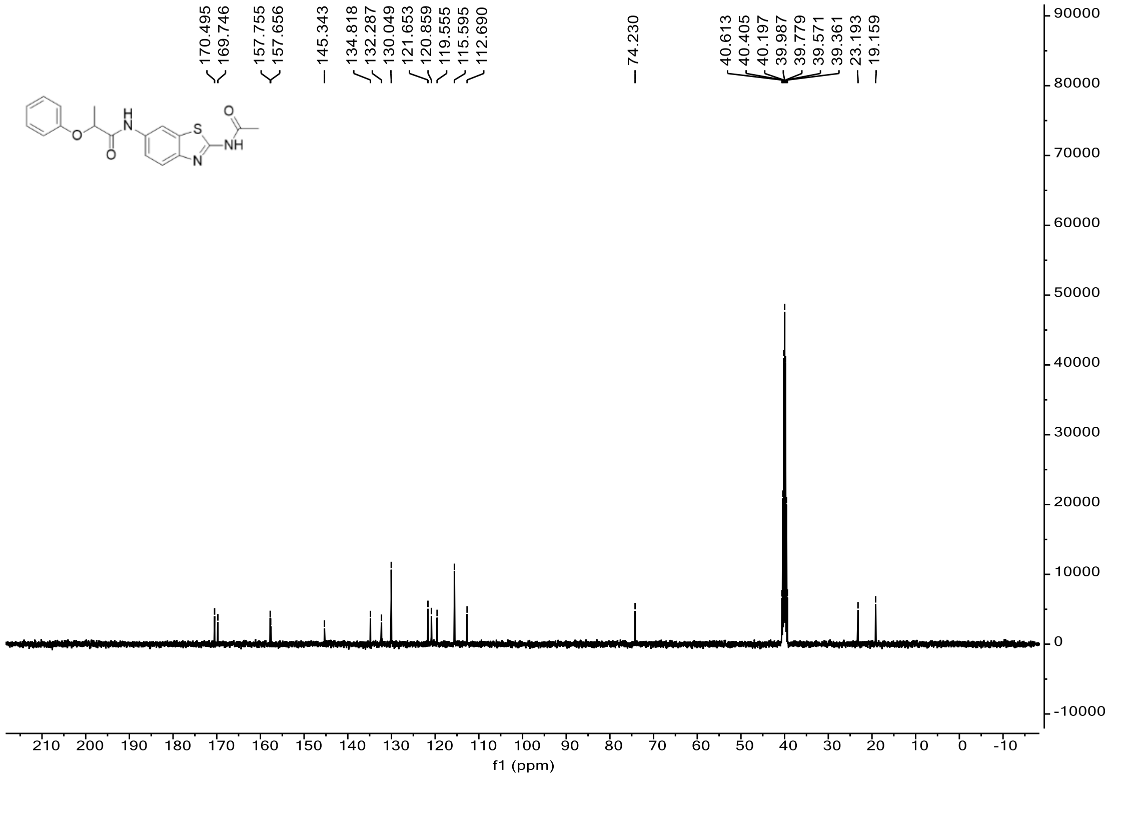

^{1}H NMR of compound **10c** (400 MHz, DMSO-*d*6)

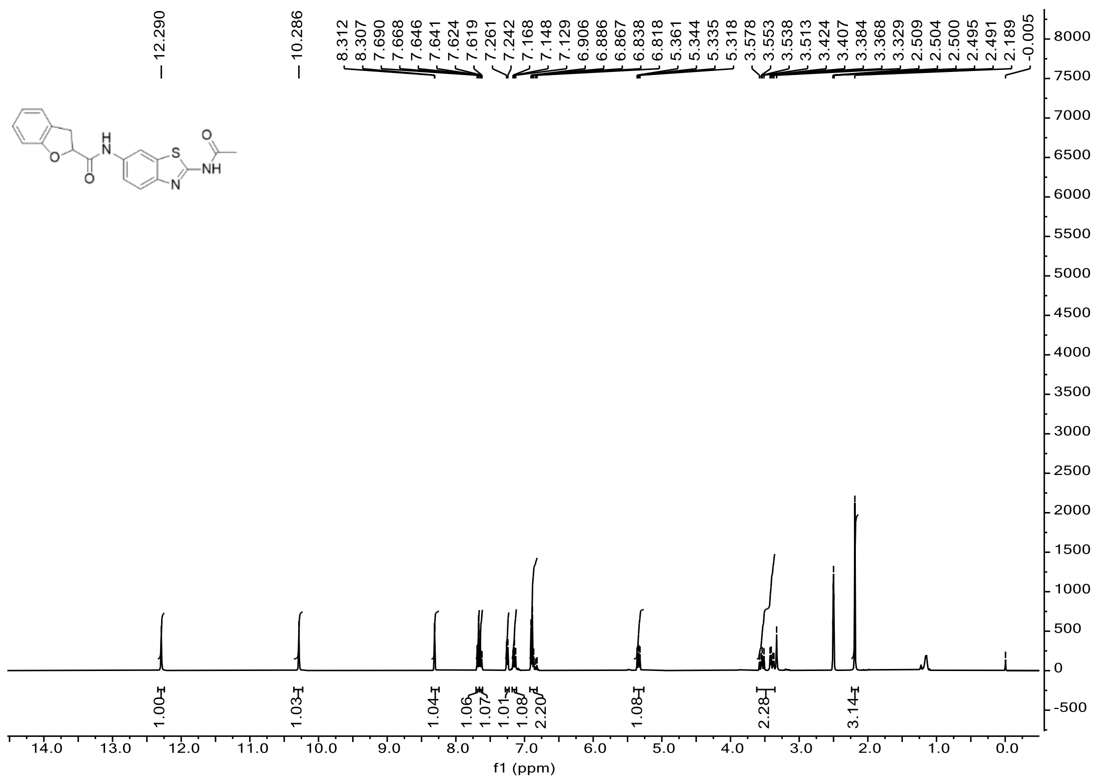

^{13}C NMR of compound **10c** (100 MHz, DMSO-*d*6)

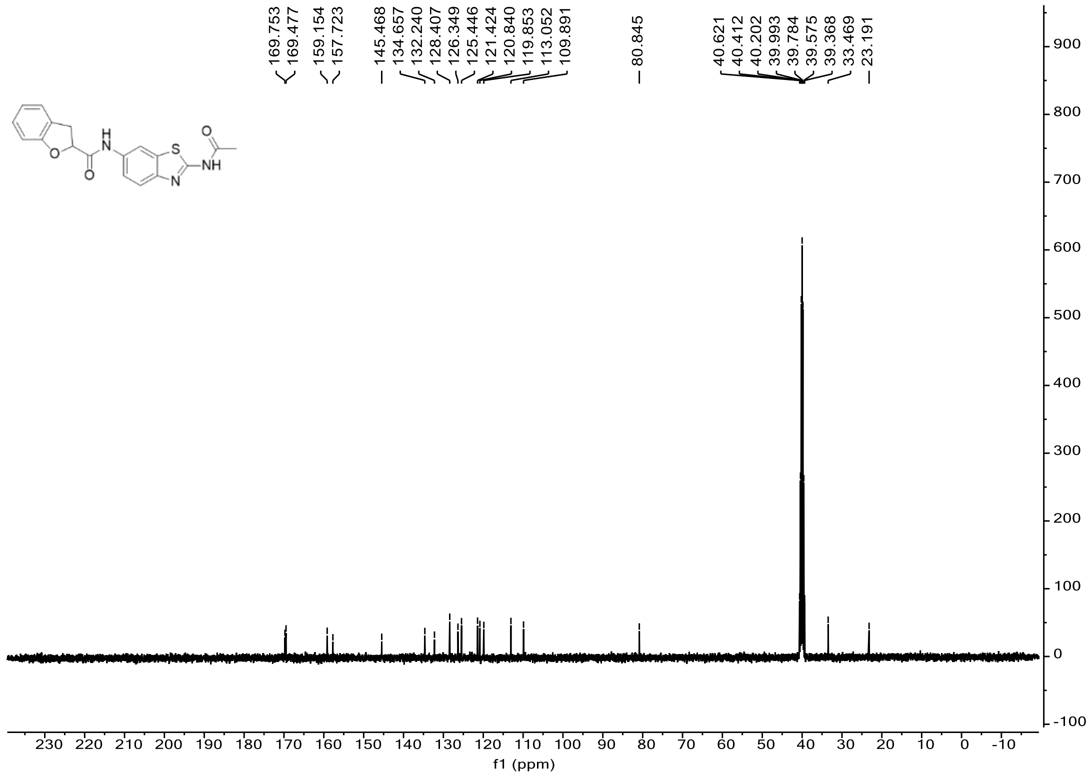

^{1}H NMR of compound **10d** (400 MHz, DMSO-d_6)

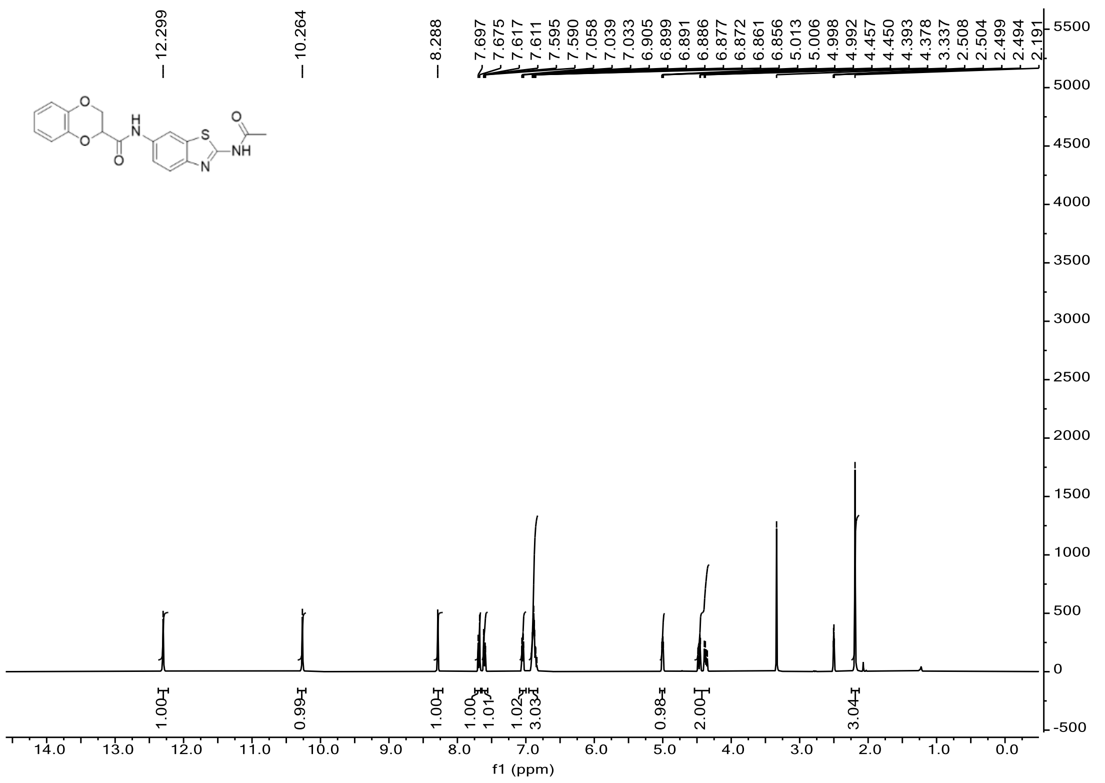

^{13}C NMR of compound **10d** (100 MHz, DMSO-d_6)

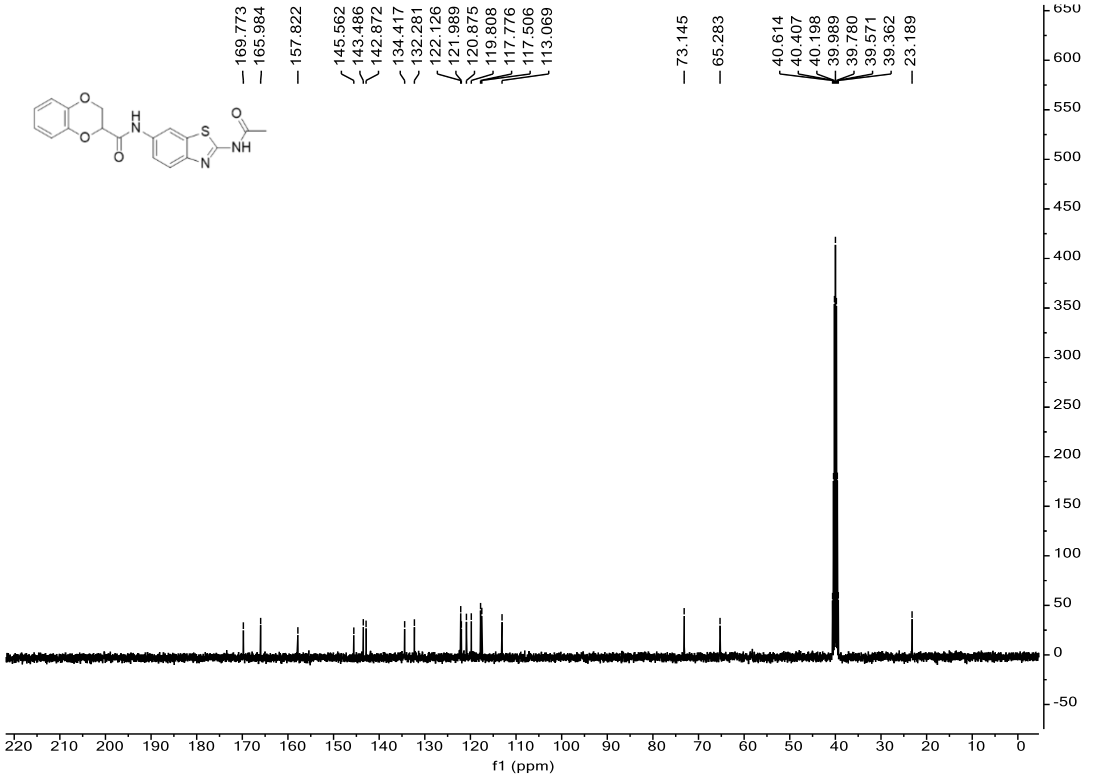

^{1}H NMR of compound **10e** (400 MHz, DMSO-d_6)

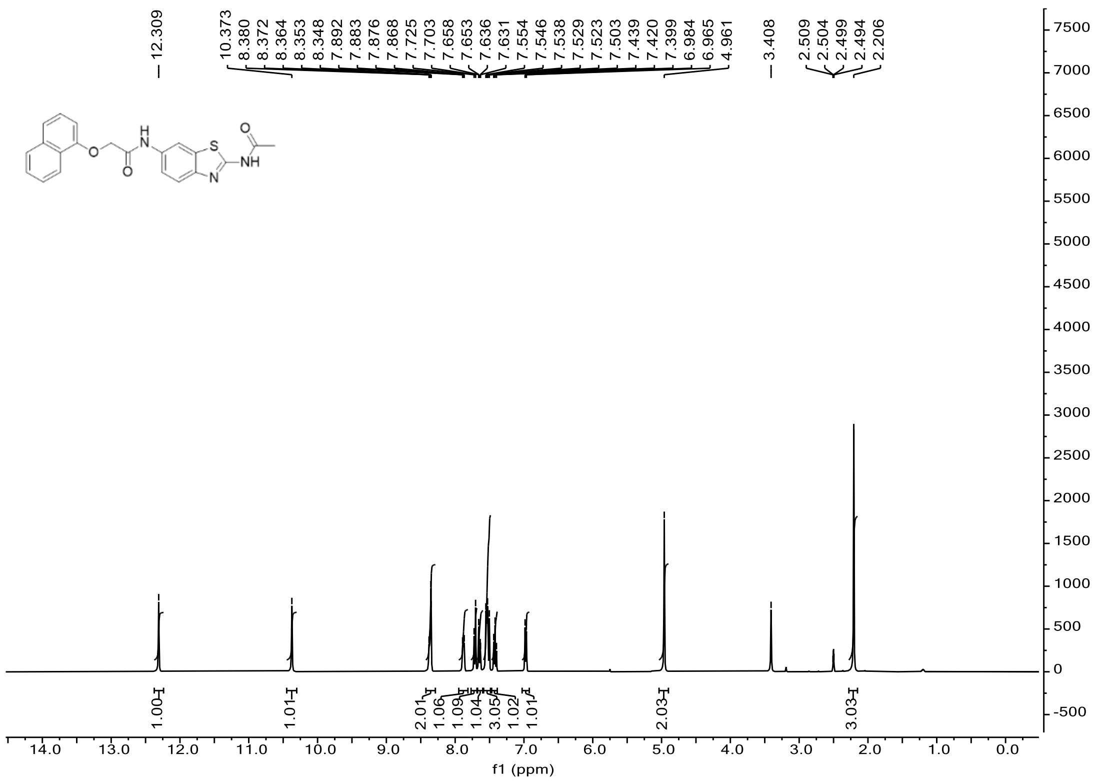

^{13}C NMR of compound **10e** (100 MHz, DMSO-d_6)

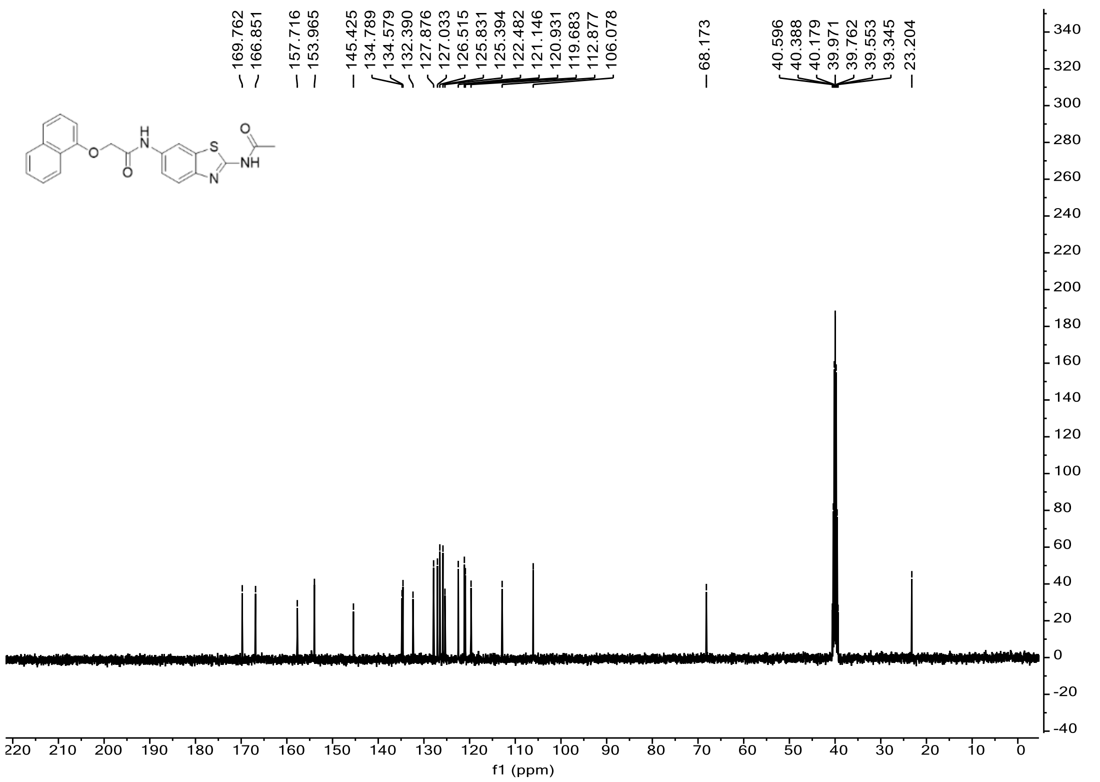

^{1}H NMR of compound **10f** (400 MHz, DMSO-d_6)

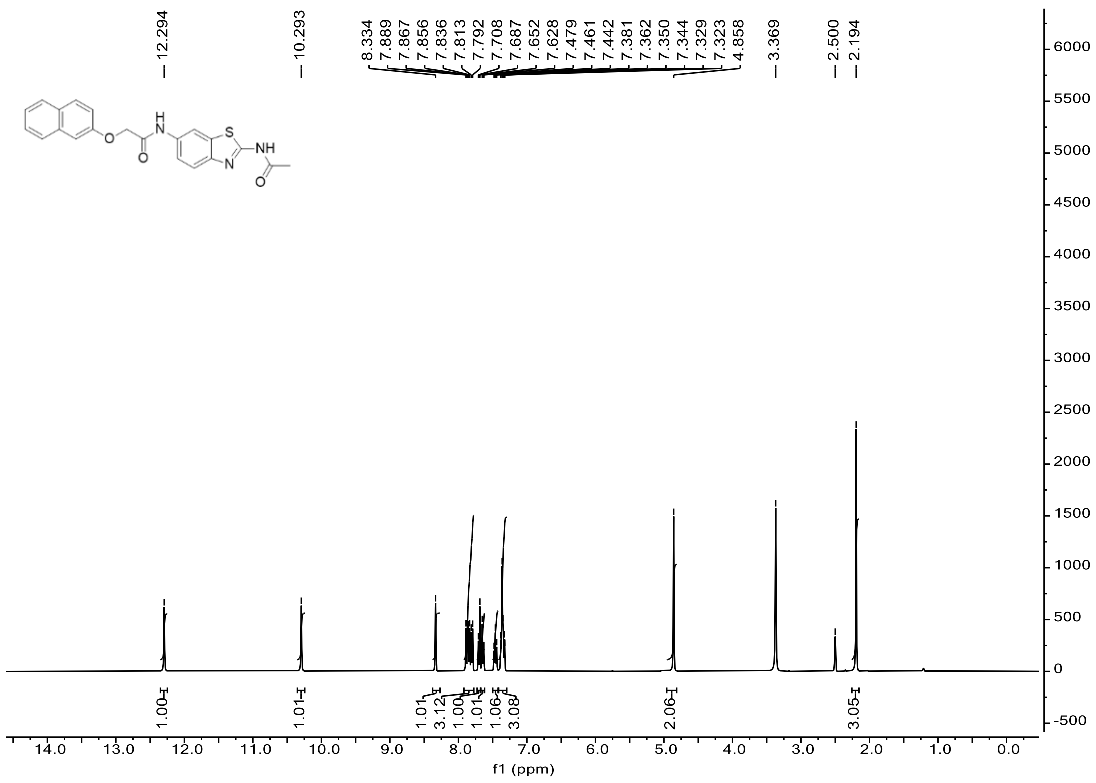

^{13}C NMR of compound **10f** (100 MHz, DMSO-d_6)

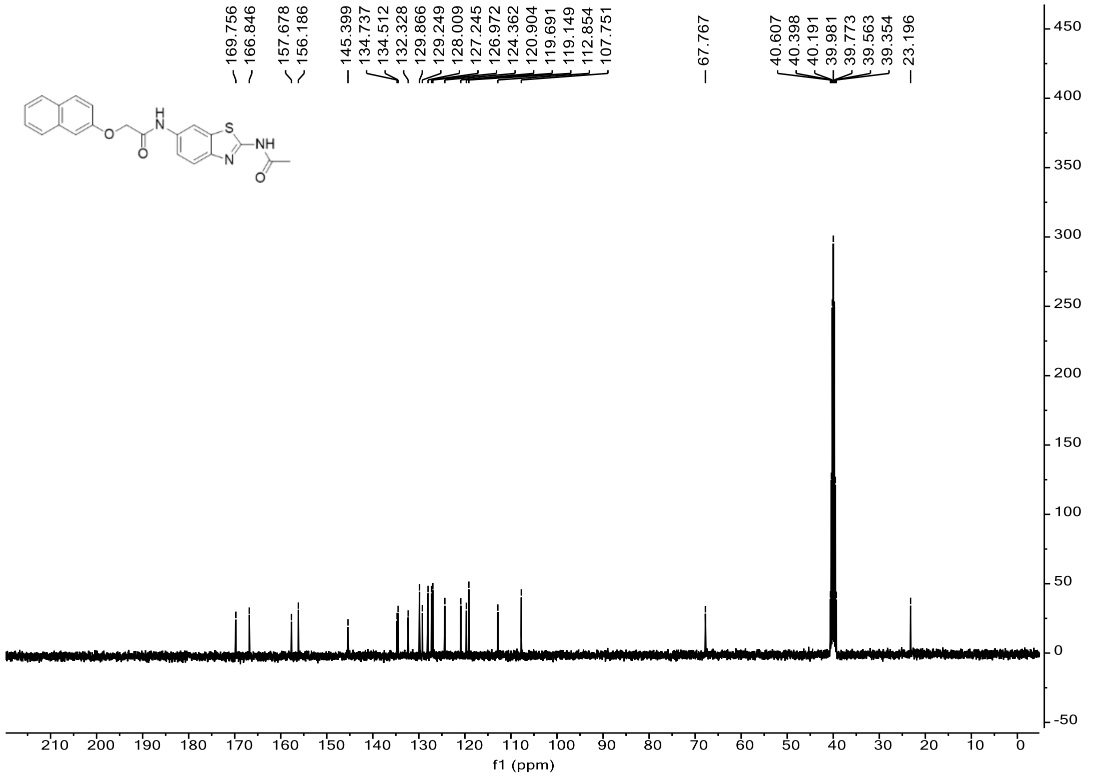

^1H NMR of compound **10g** (400 MHz, DMSO-d_6)

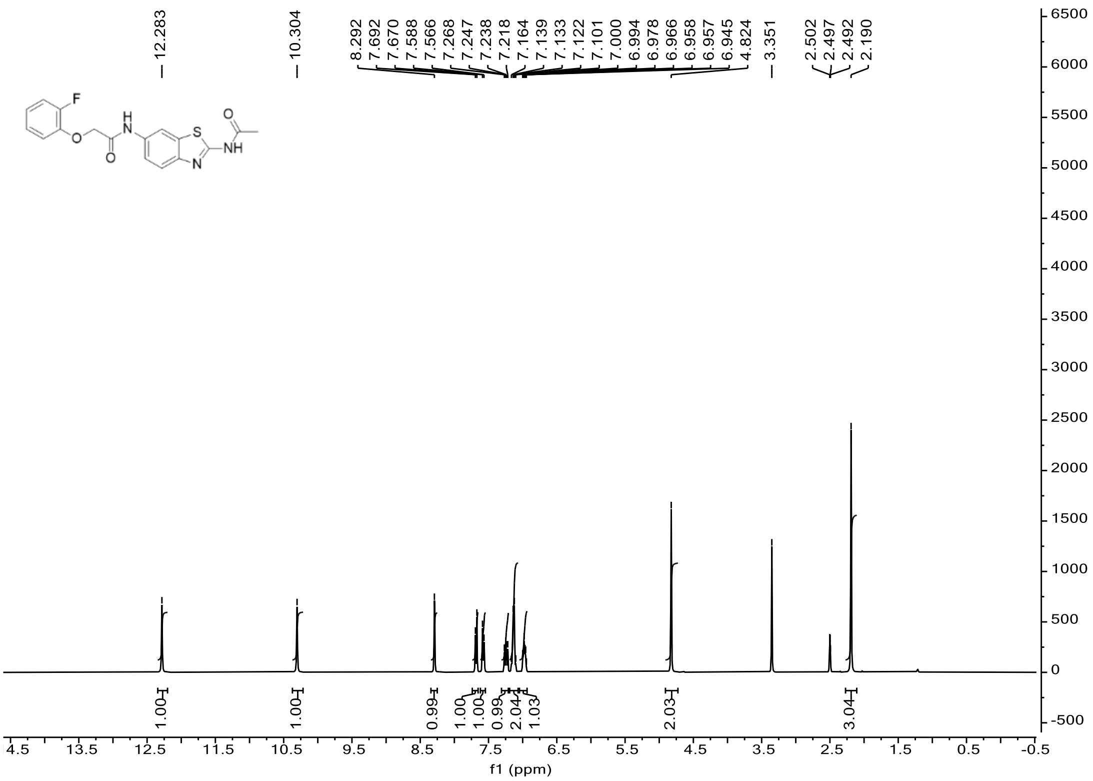

^{13}C NMR of compound **10g** (100 MHz, DMSO-d_6)

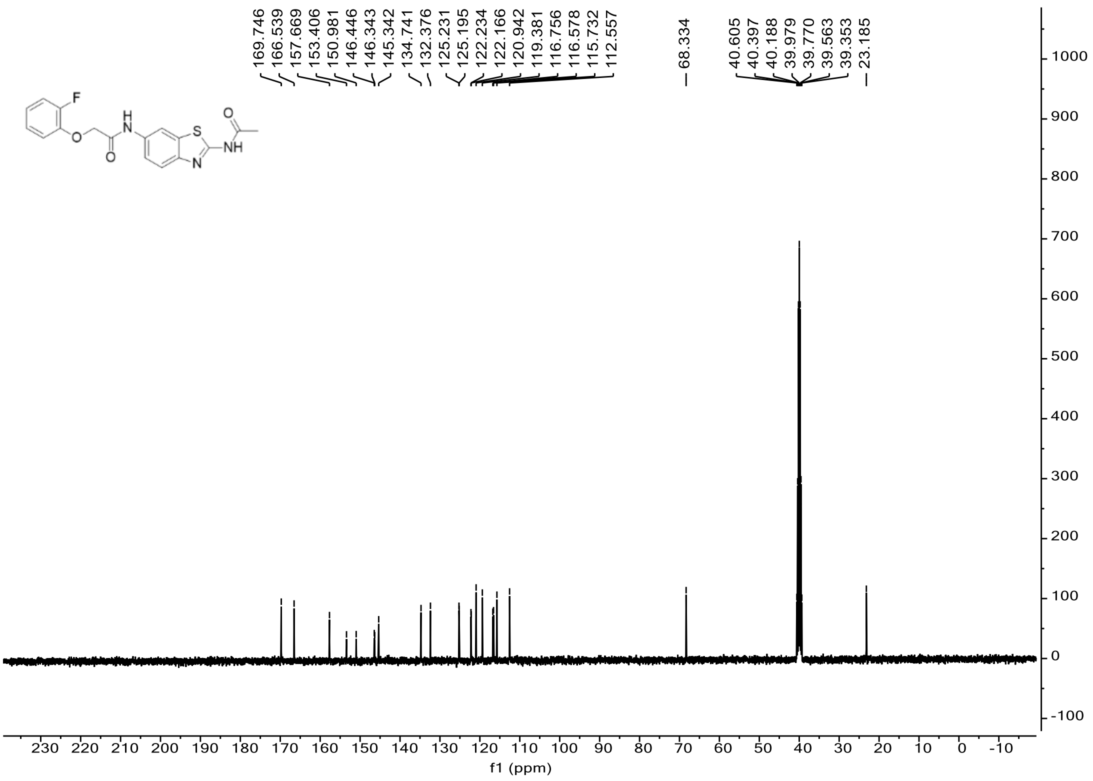

^{19}F NMR of compound **10g** (376 MHz, DMSO-d_6)

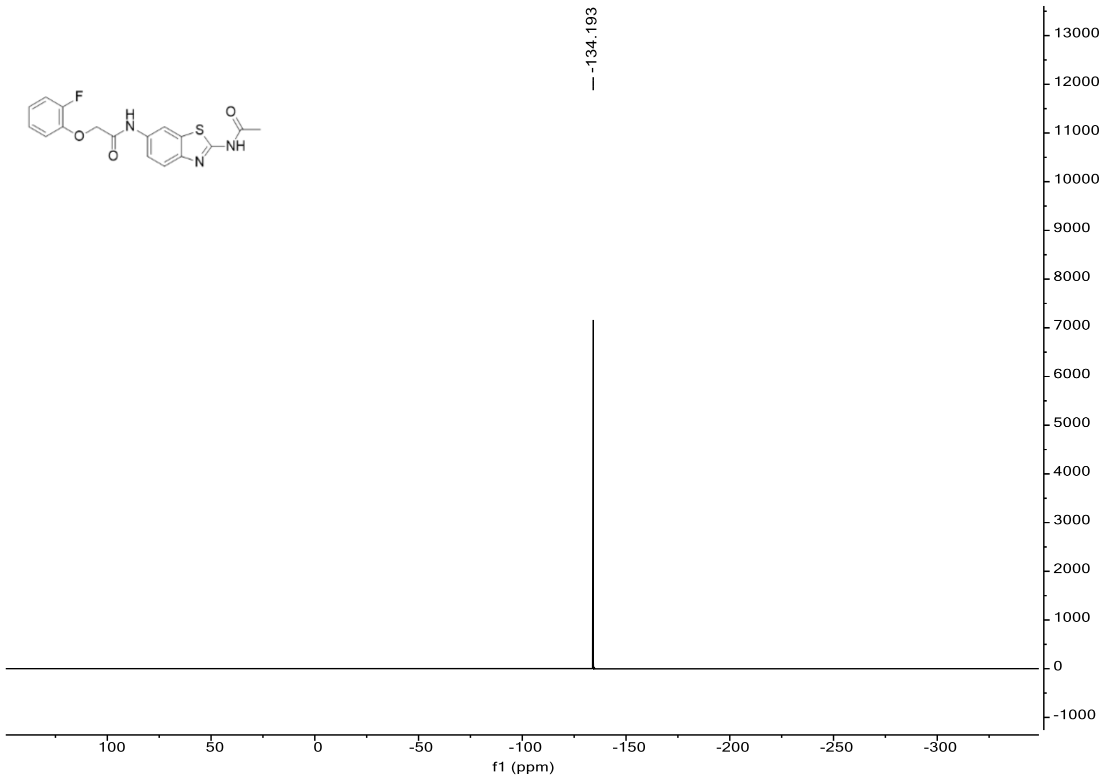

^{1}H NMR of compound **10h** (400 MHz, DMSO-d_6)

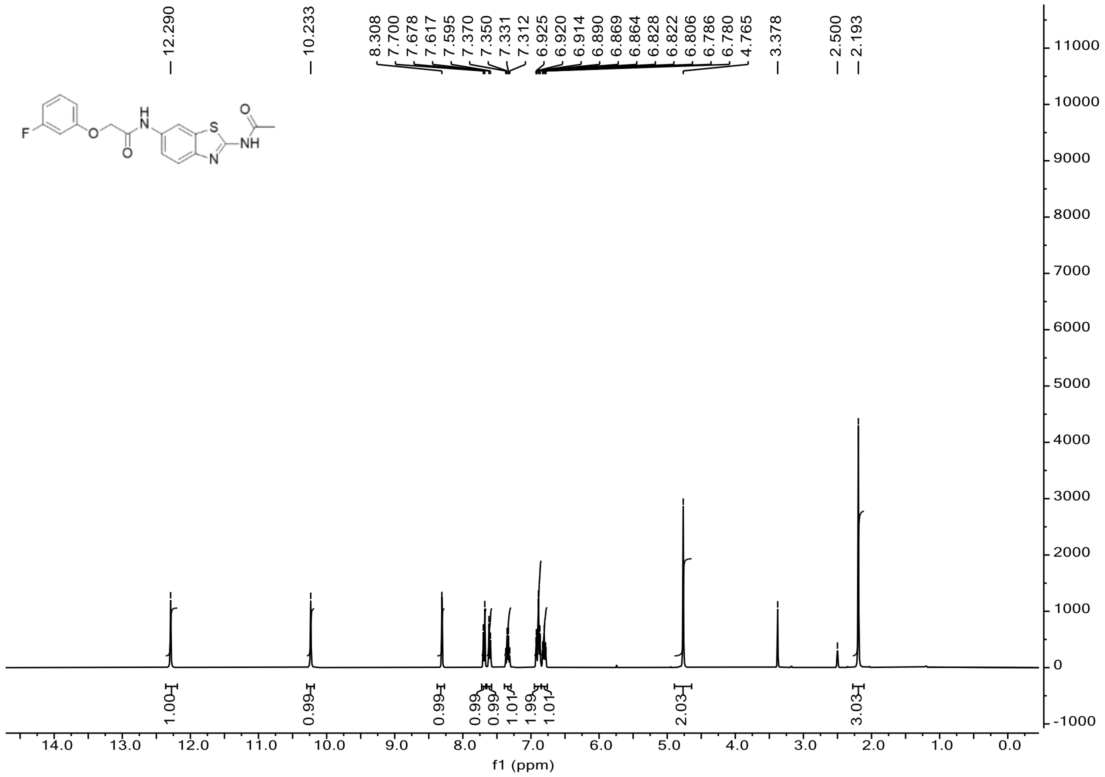

^{13}C NMR of compound **10h** (100 MHz, DMSO-d_6)

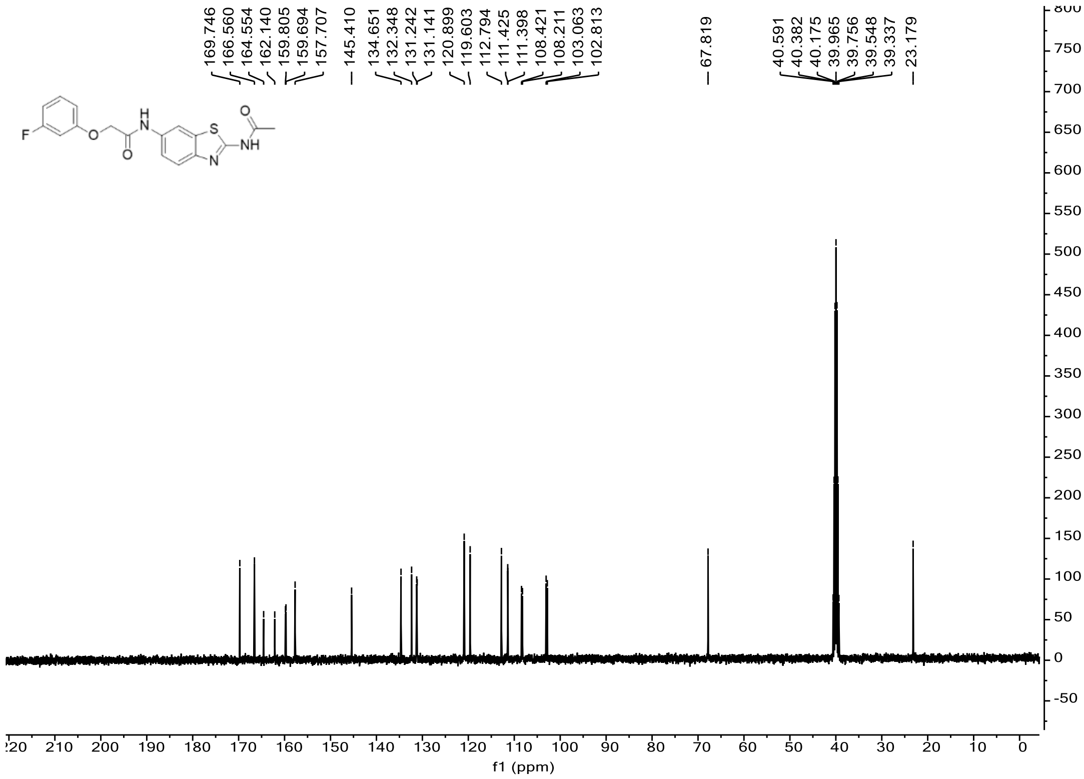

^{19}F NMR of compound **10h** (376 MHz, DMSO-d_6)

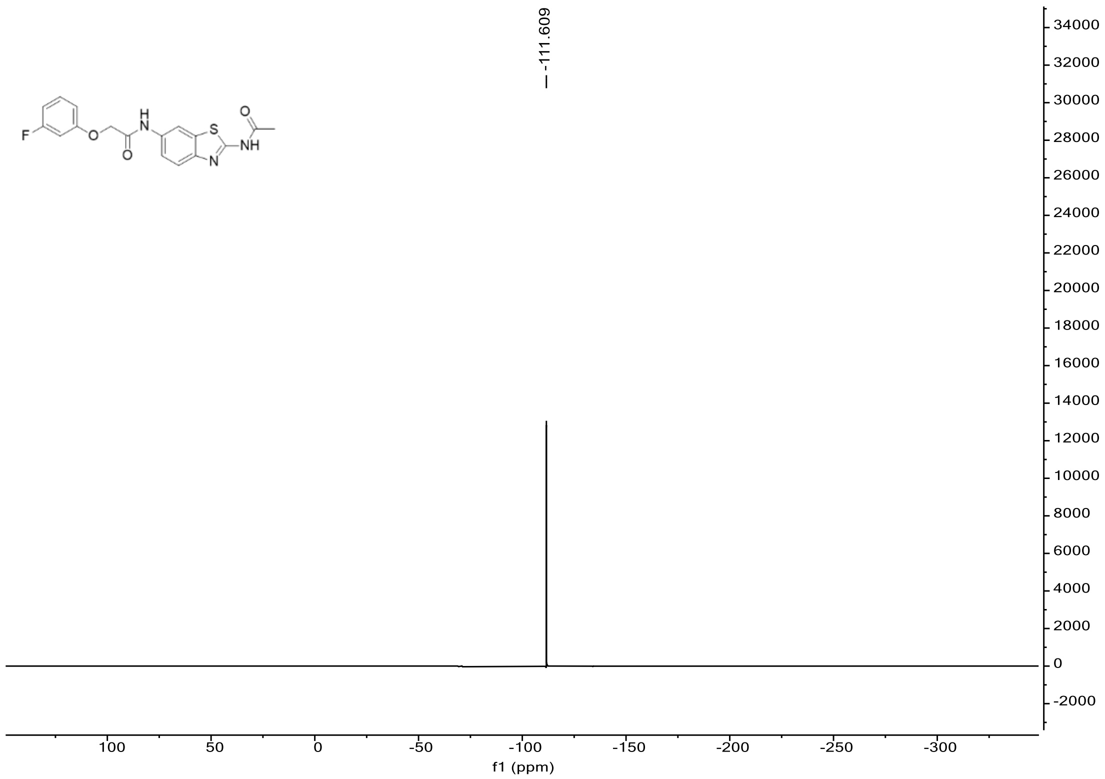

^{1}H NMR of compound **10i** (400 MHz, DMSO-d_6)

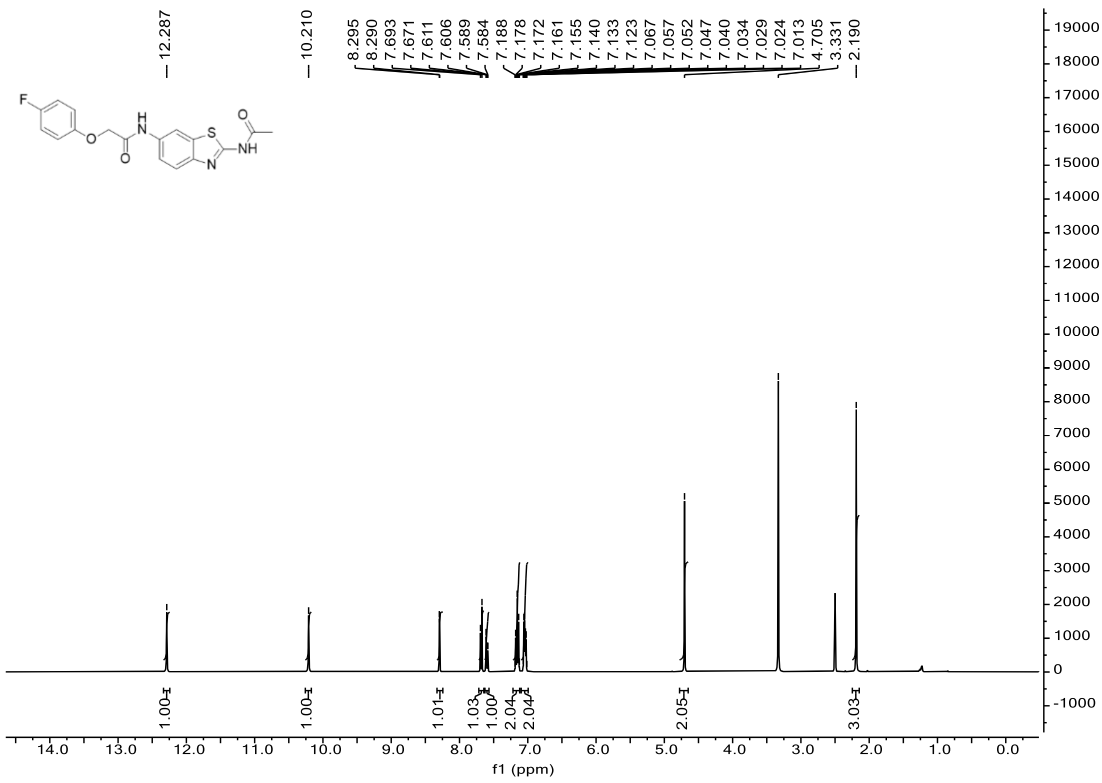

^{13}C NMR of compound **10i** (100 MHz, DMSO-d_6)

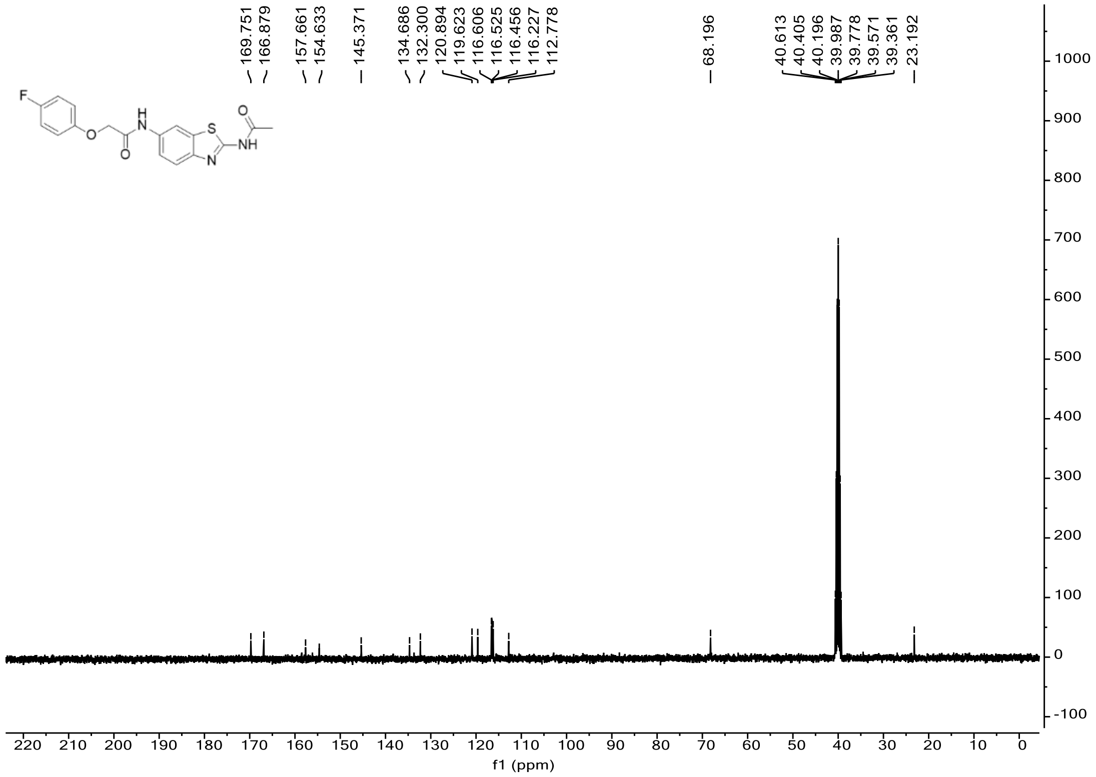

^{19}F NMR of compound **10i** (376 MHz, DMSO-d_6)

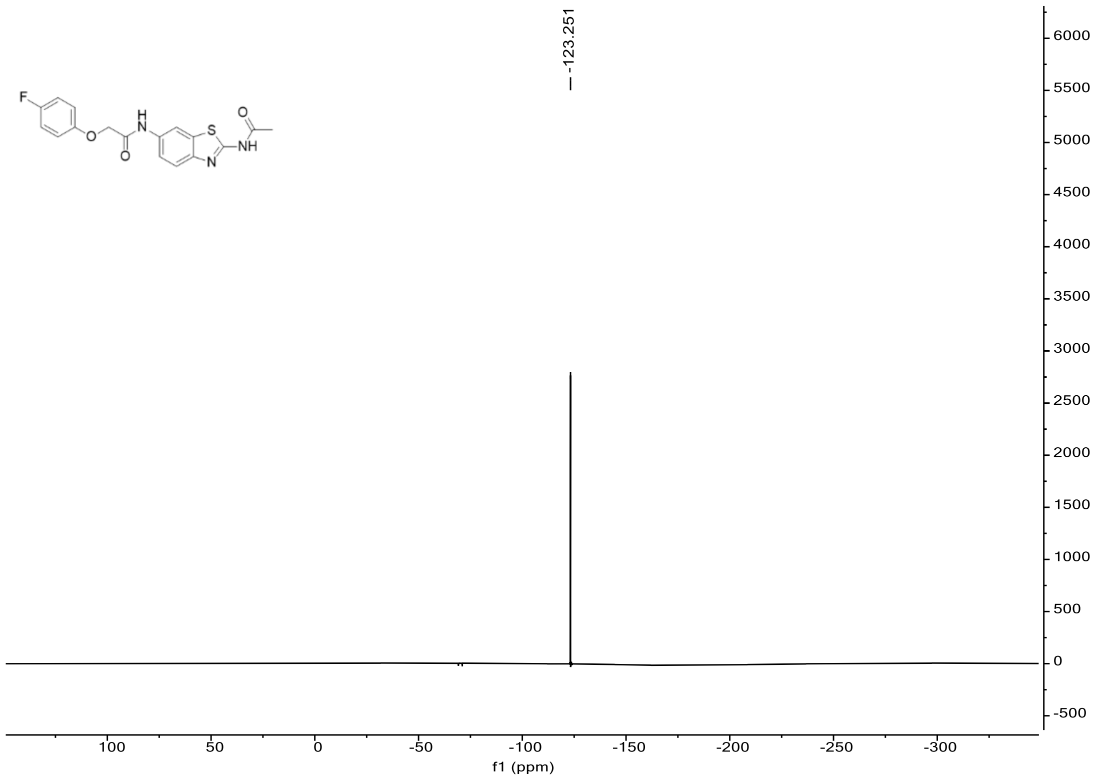

^{1}H NMR of compound **10j** (400 MHz, DMSO-d_6)

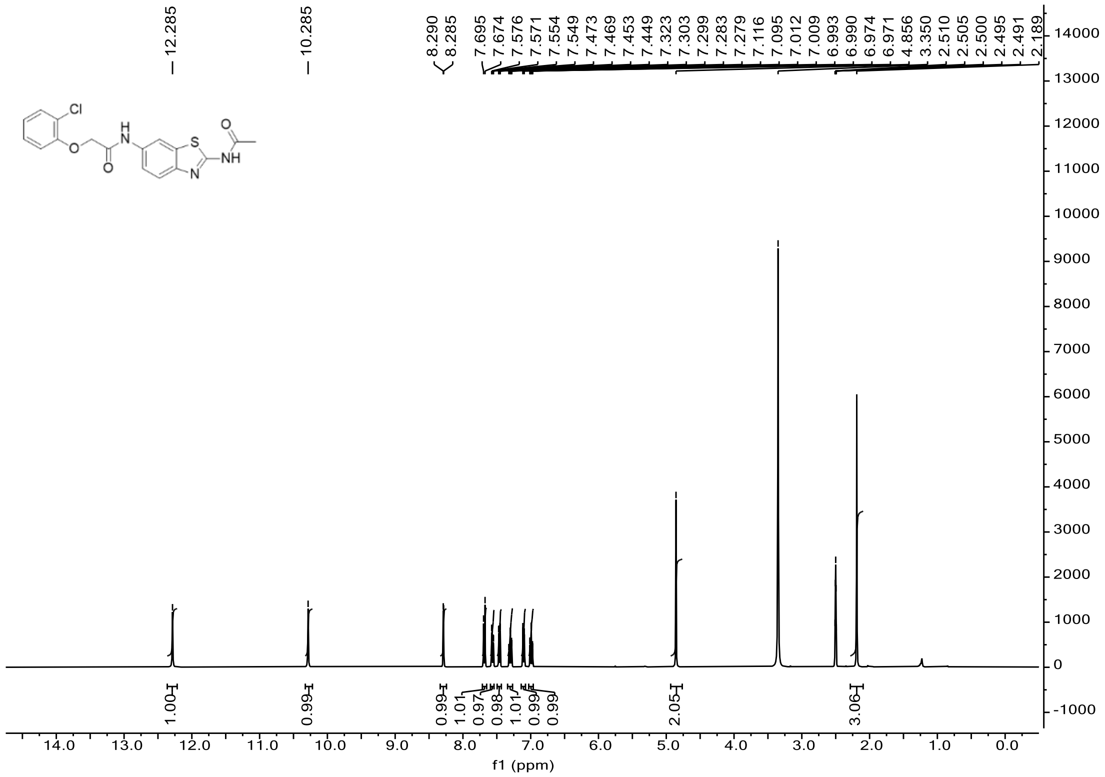

^{13}C NMR of compound **10j** (100 MHz, DMSO-*d*6)

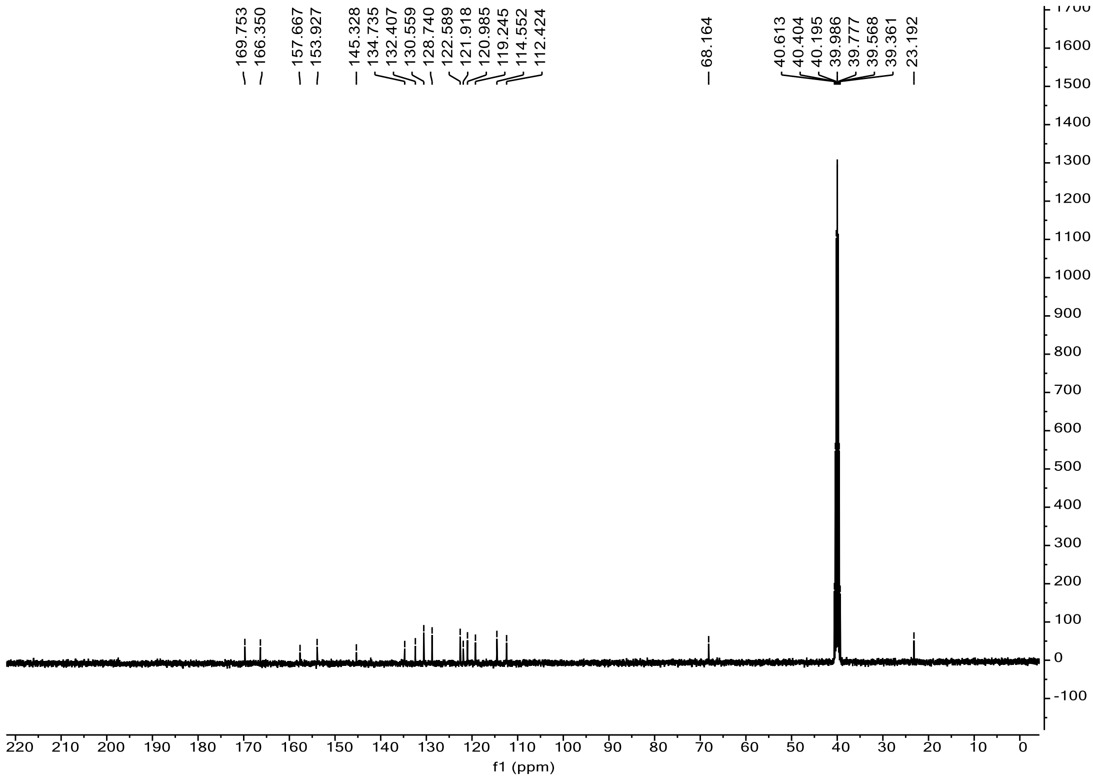

^{1}H NMR of compound **10k** (400 MHz, DMSO-*d*6)

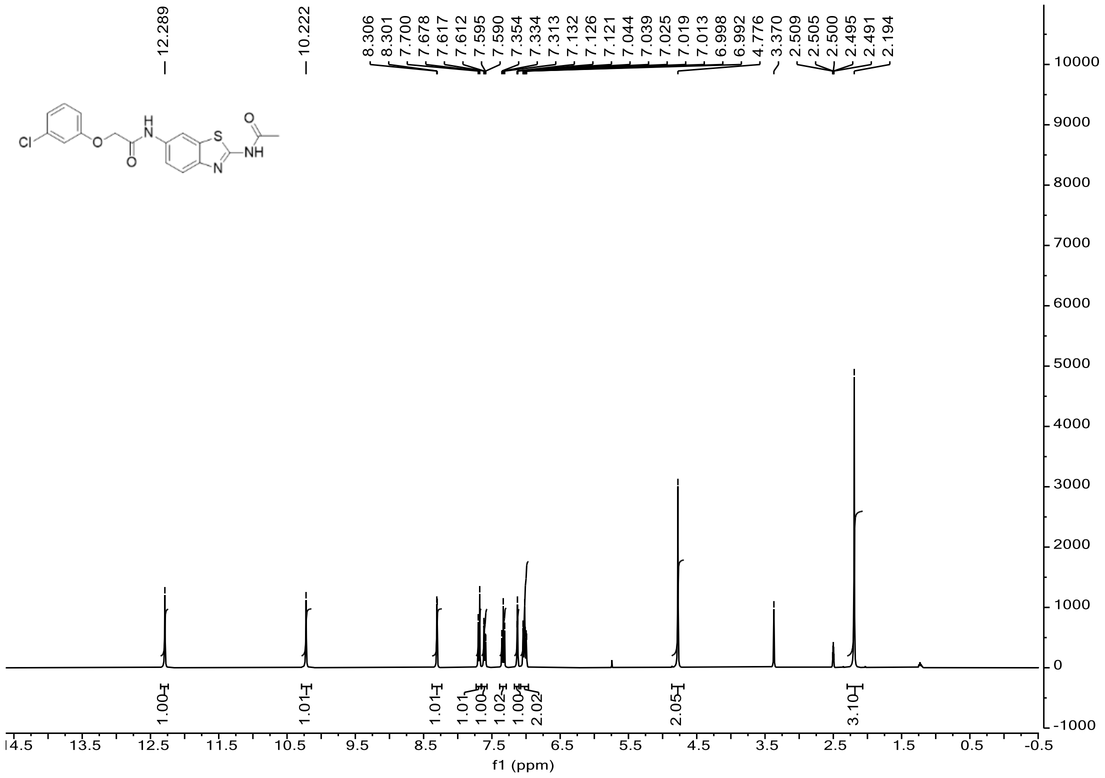

^{13}C NMR of compound **10k** (100 MHz, DMSO-d_6)

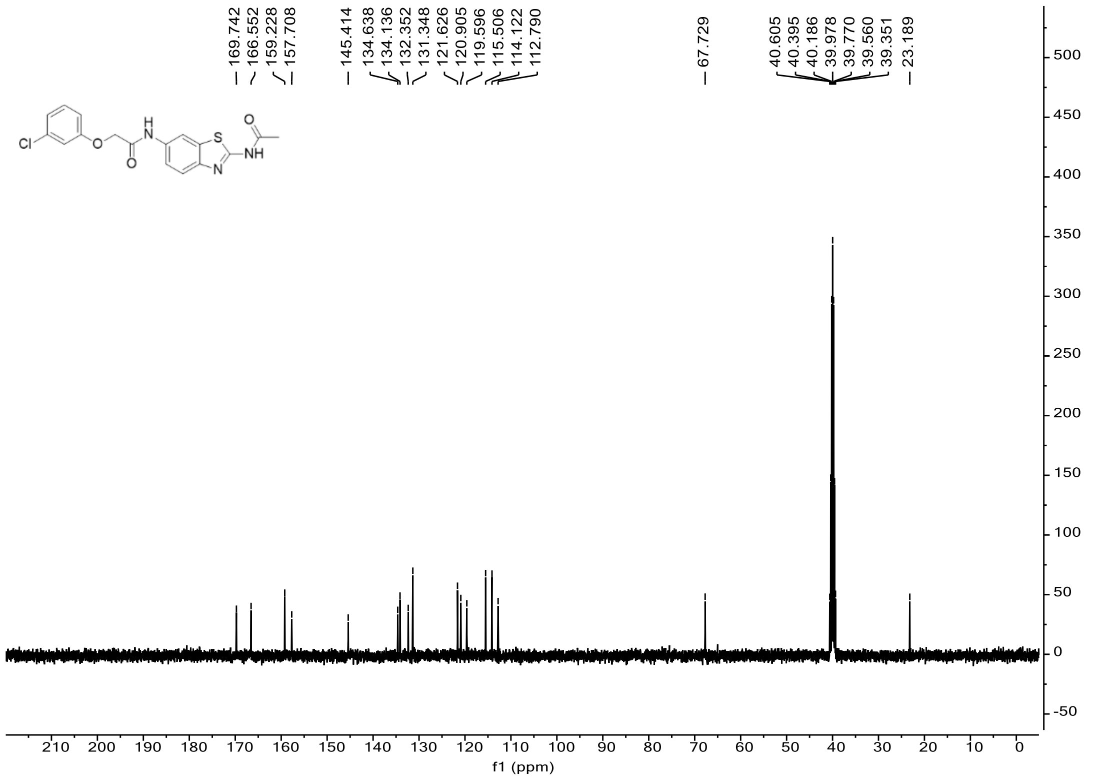

^{1}H NMR of compound **10l** (400 MHz, DMSO-d_6)

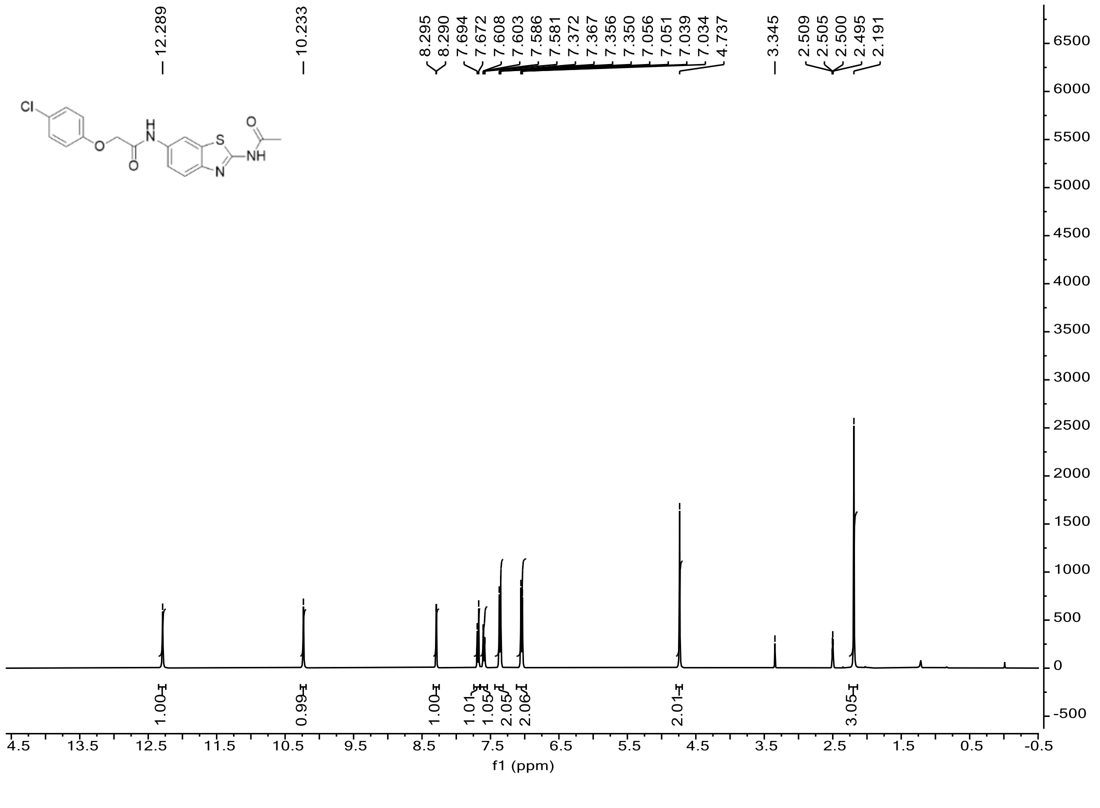

^{13}C NMR of compound **10l** (100 MHz, DMSO-d_6)

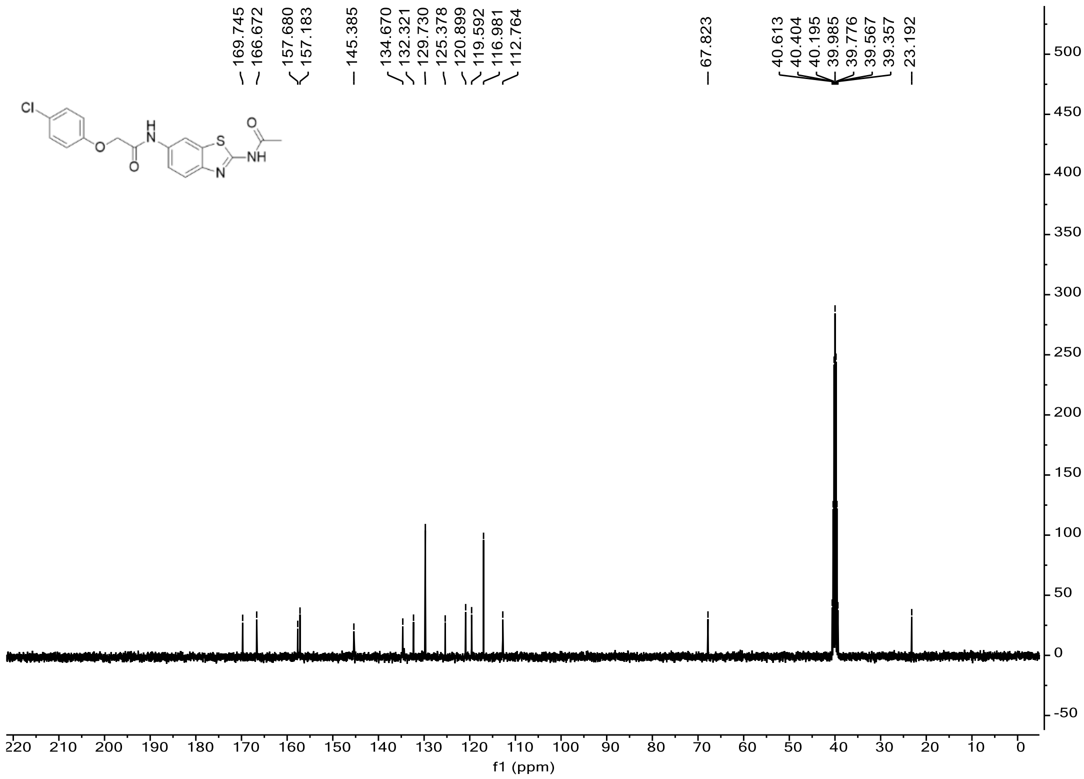

^1H NMR of compound **10m** (400 MHz, DMSO-d_6)

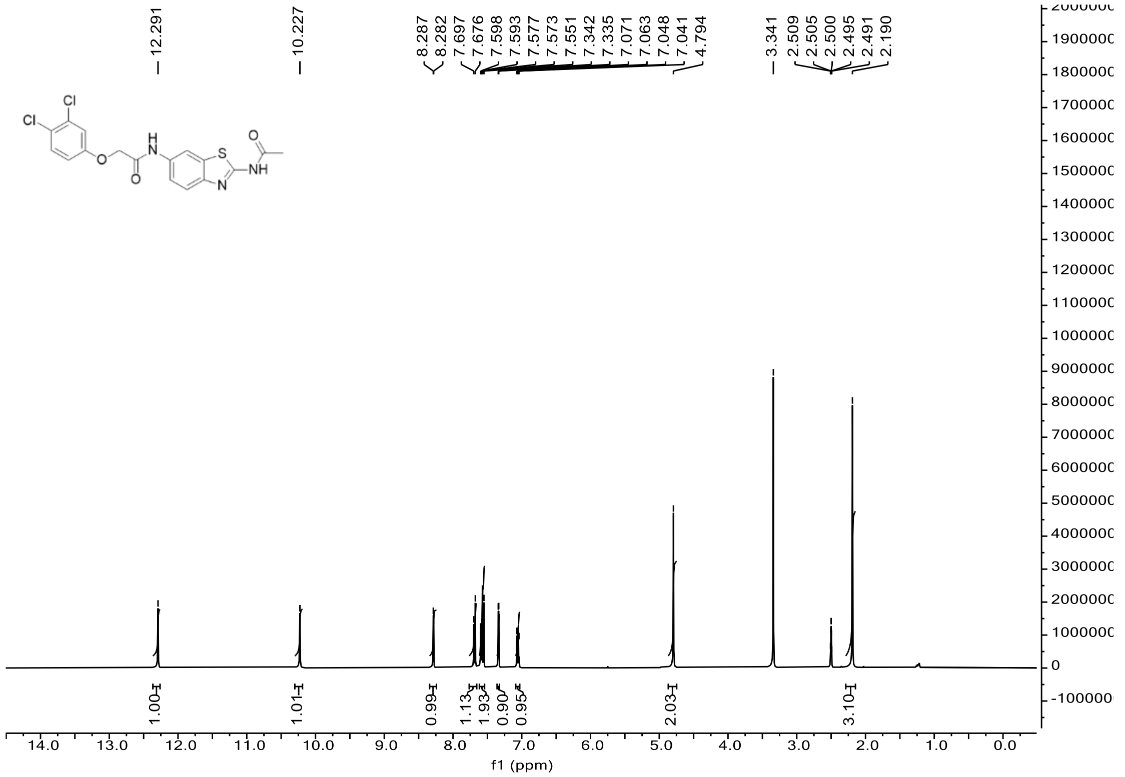

^{13}C NMR of compound **10m** (100 MHz, DMSO-d_6)

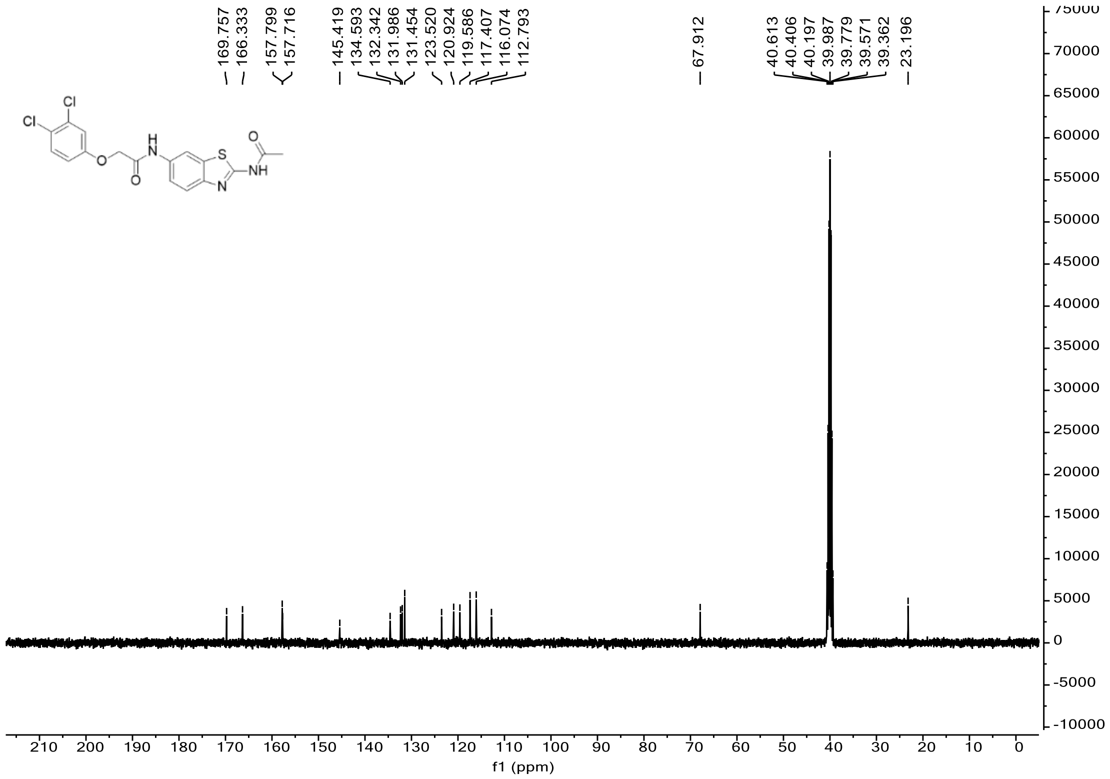

^{1}H NMR of compound **10n** (400 MHz, DMSO-d_6)

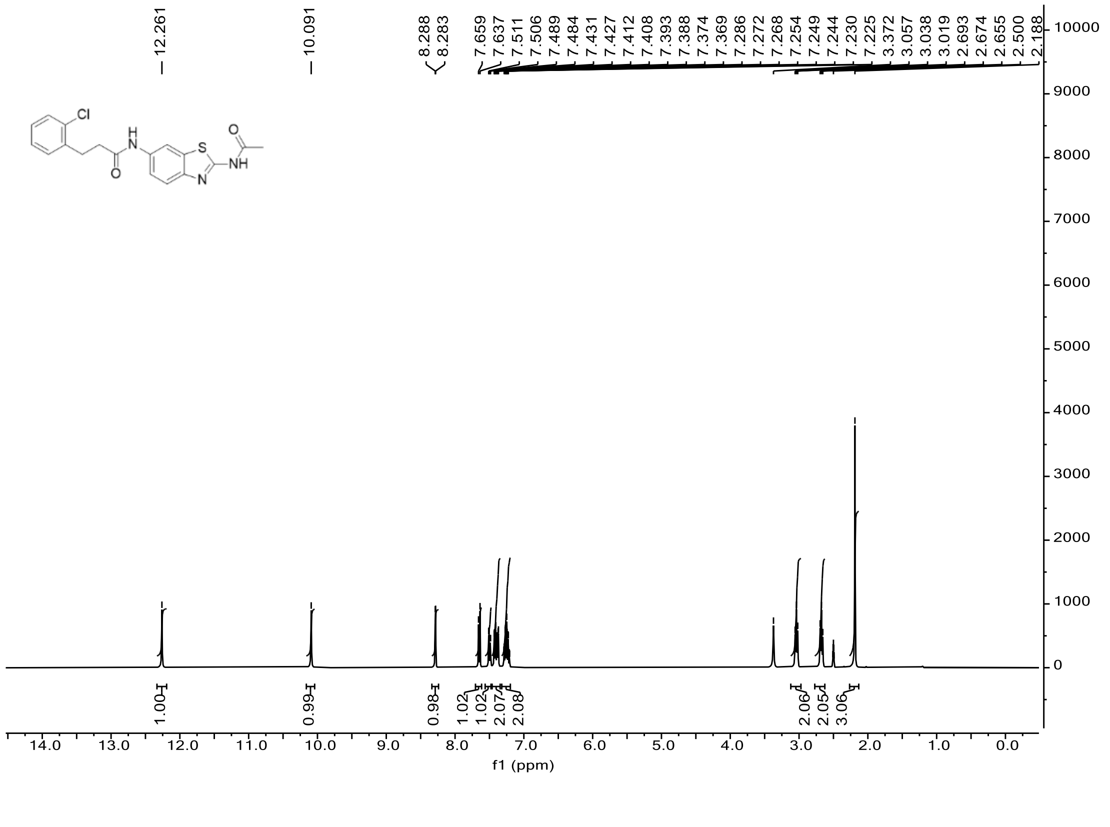

^{13}C NMR of compound **10n** (100 MHz, DMSO-d_6)

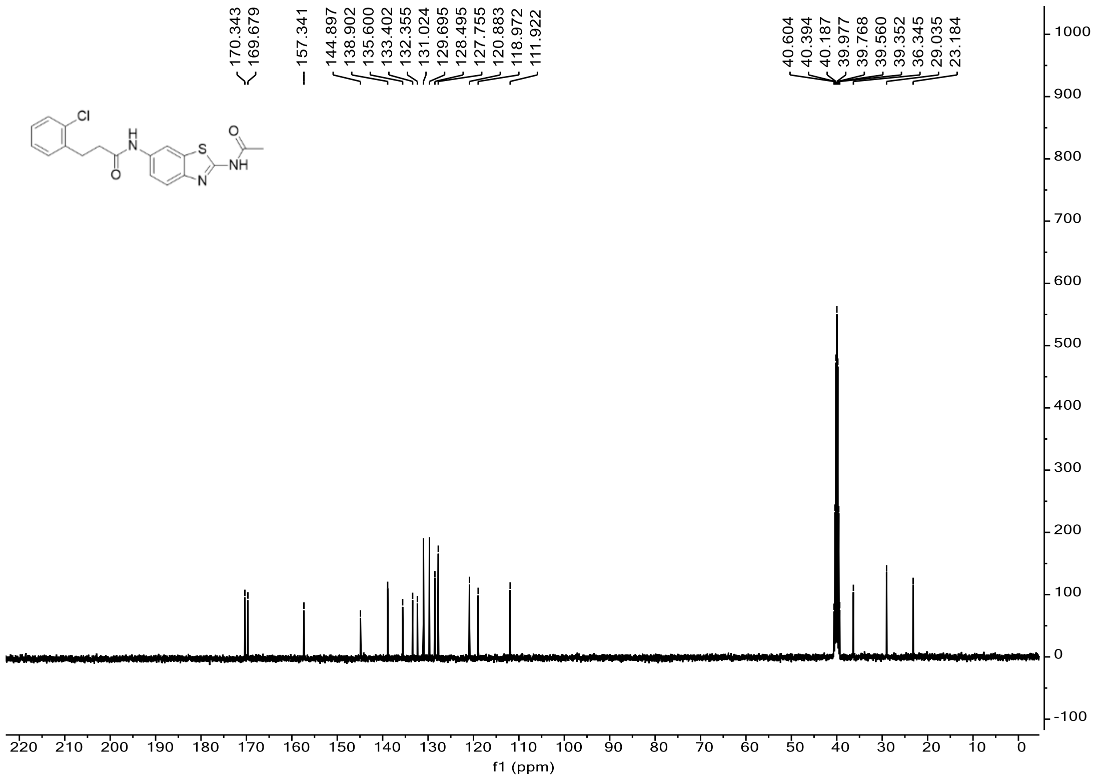

^{1}H NMR of compound **10o** (400 MHz, DMSO-d_6)

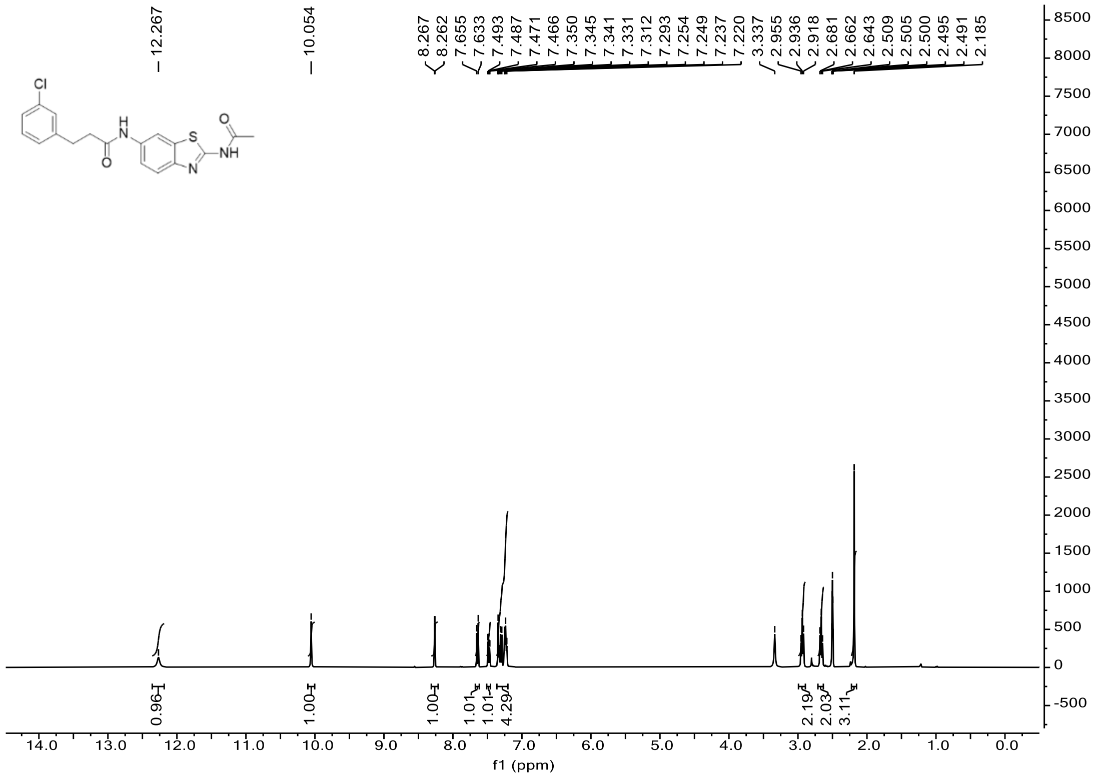

^{13}C NMR of compound **10o** (100 MHz, DMSO-d_6)

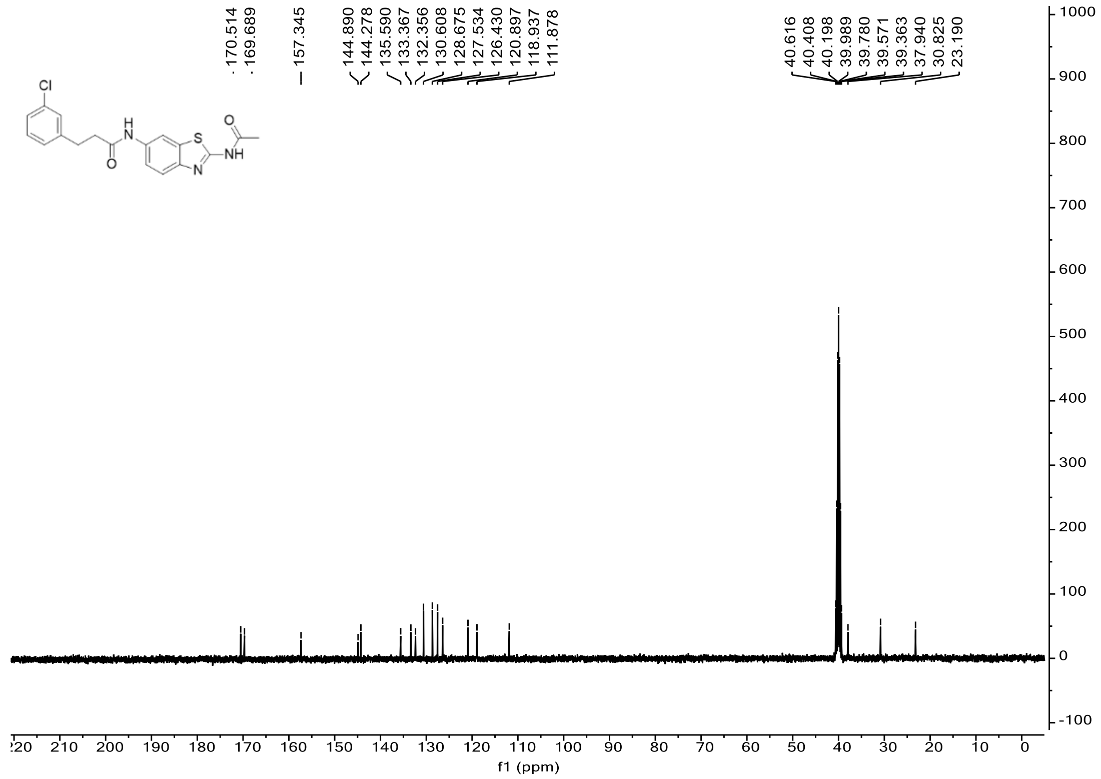

^{1}H NMR of compound **10p** (400 MHz, DMSO-d_6)

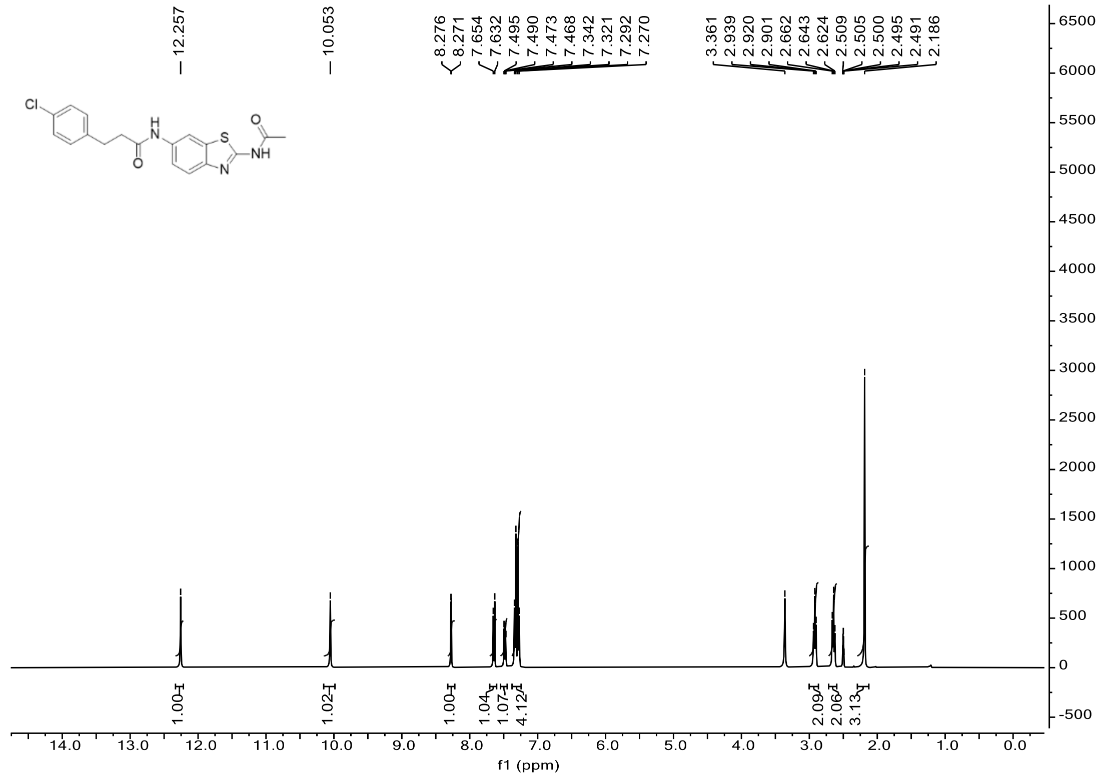

^{13}C NMR of compound **10p** (100 MHz, DMSO-d_6)

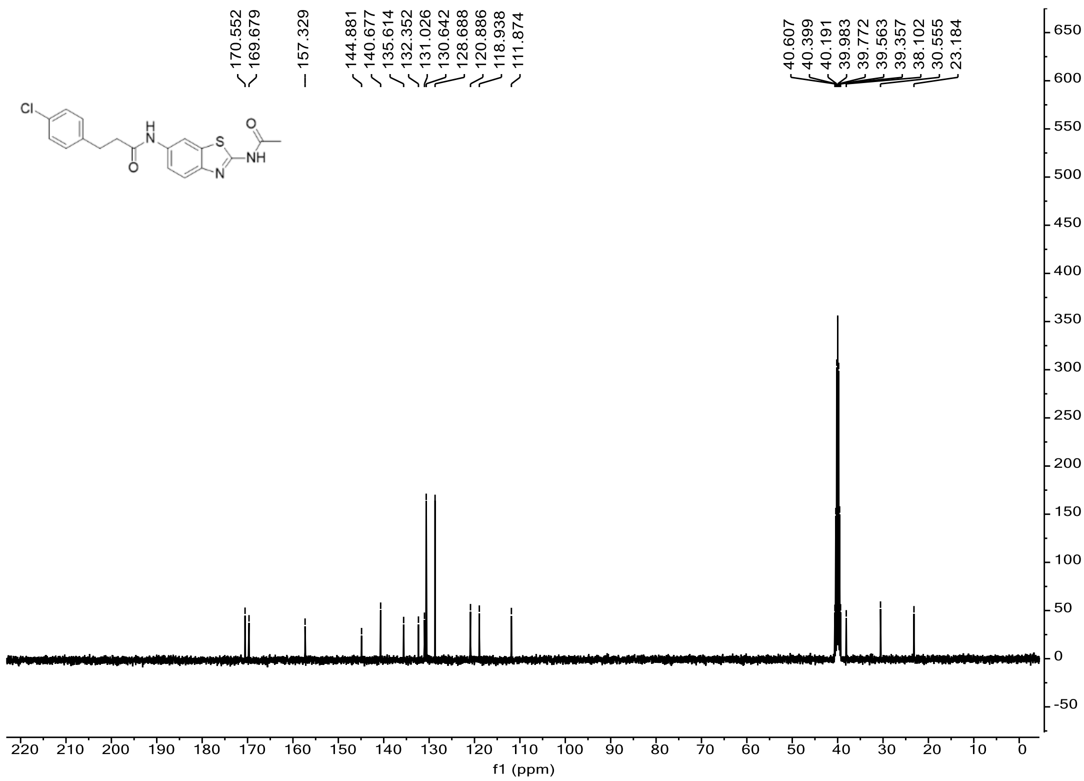

^{1}H NMR of compound **10q** (400 MHz, DMSO-d_6)

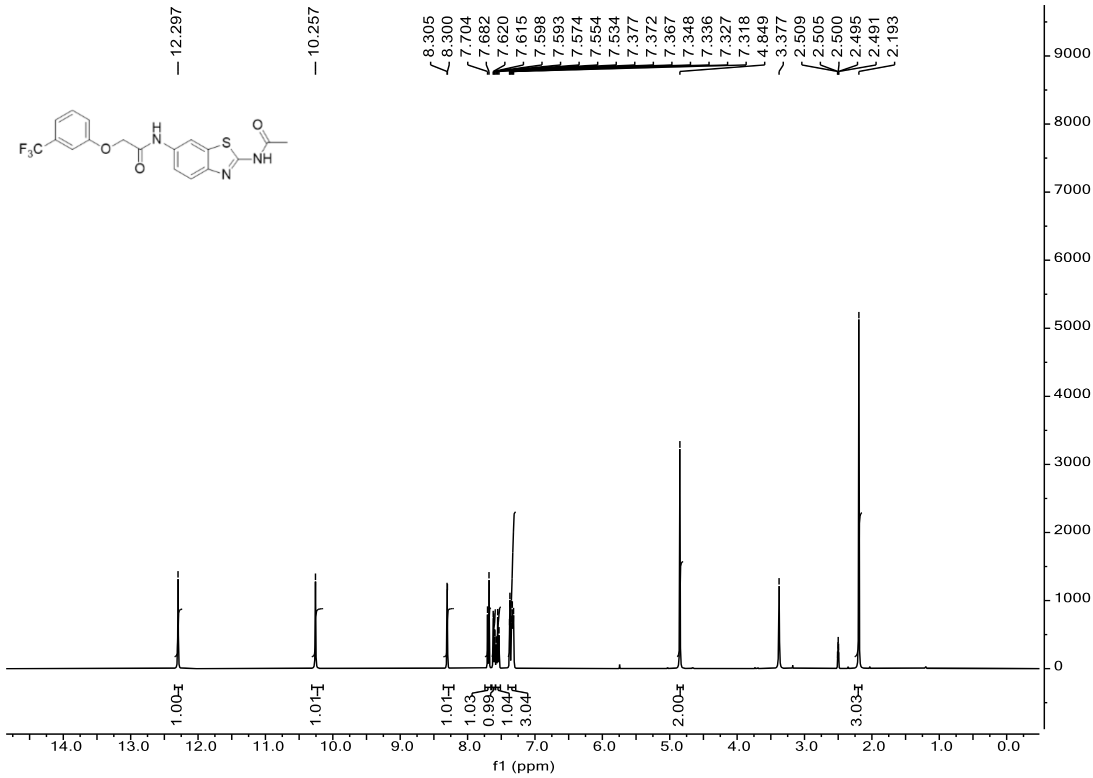

^{13}C NMR of compound **10q** (100 MHz, DMSO-*d*6)

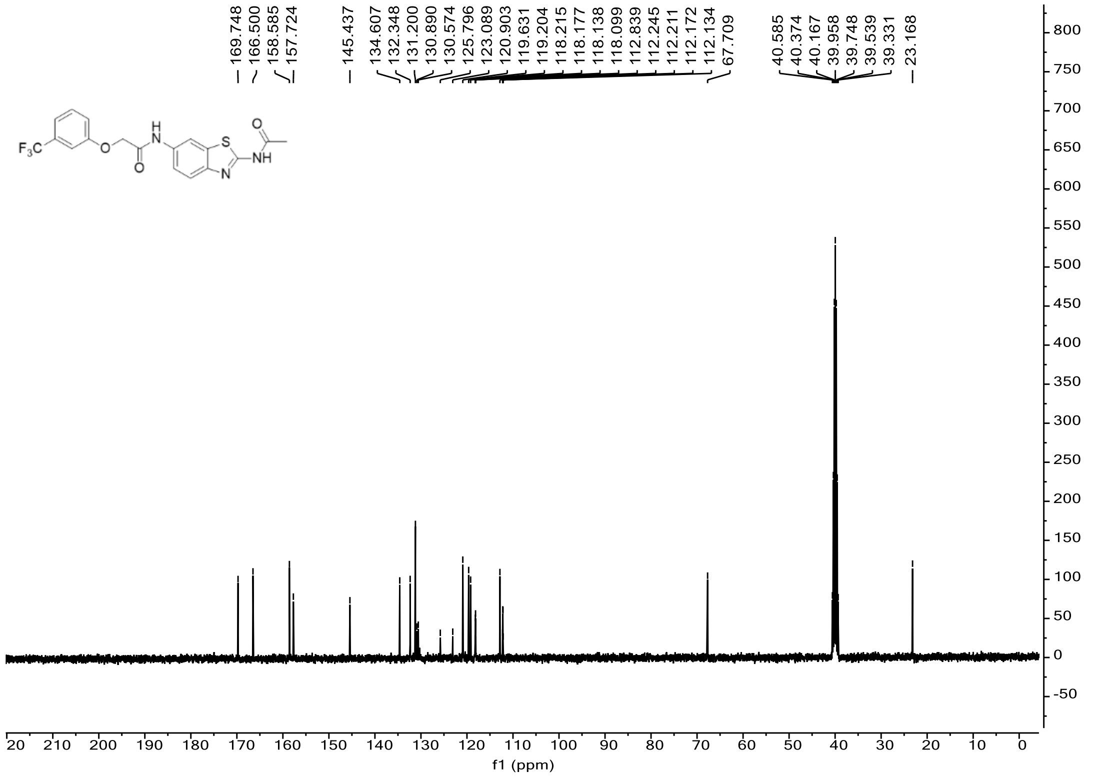

^{19}F NMR of compound **10q** (376 MHz, DMSO-*d*6)

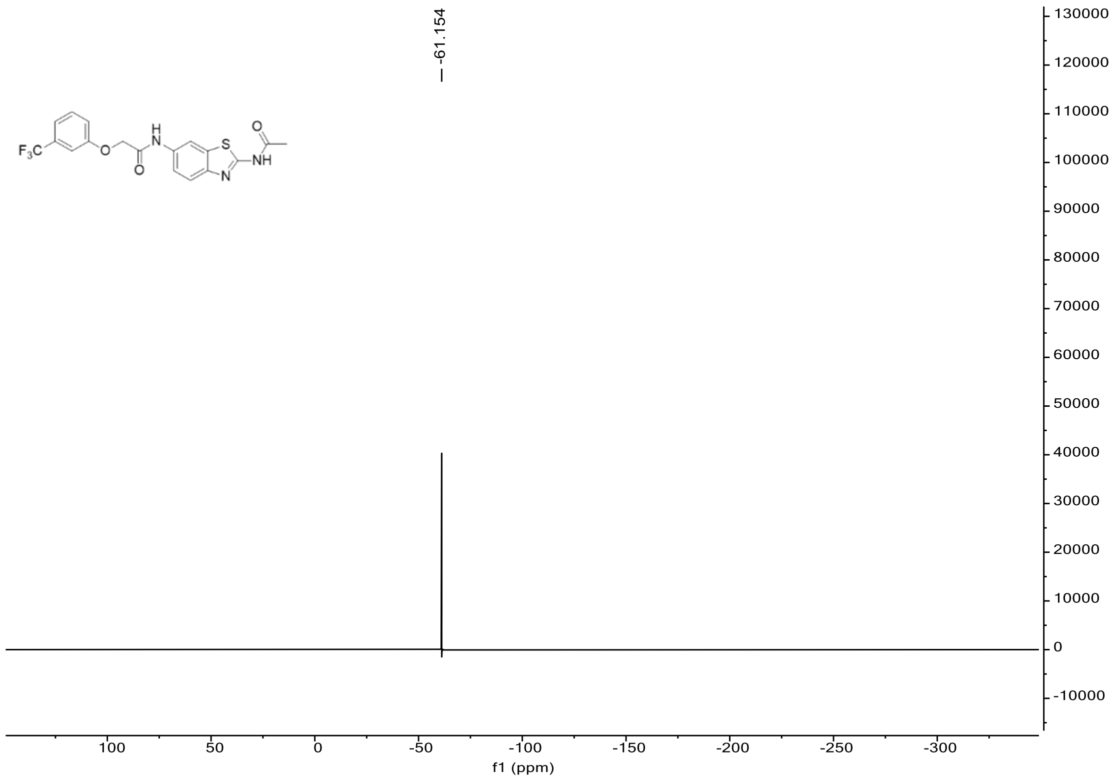

^{1}H NMR of compound **10r** (400 MHz, DMSO-d_6)

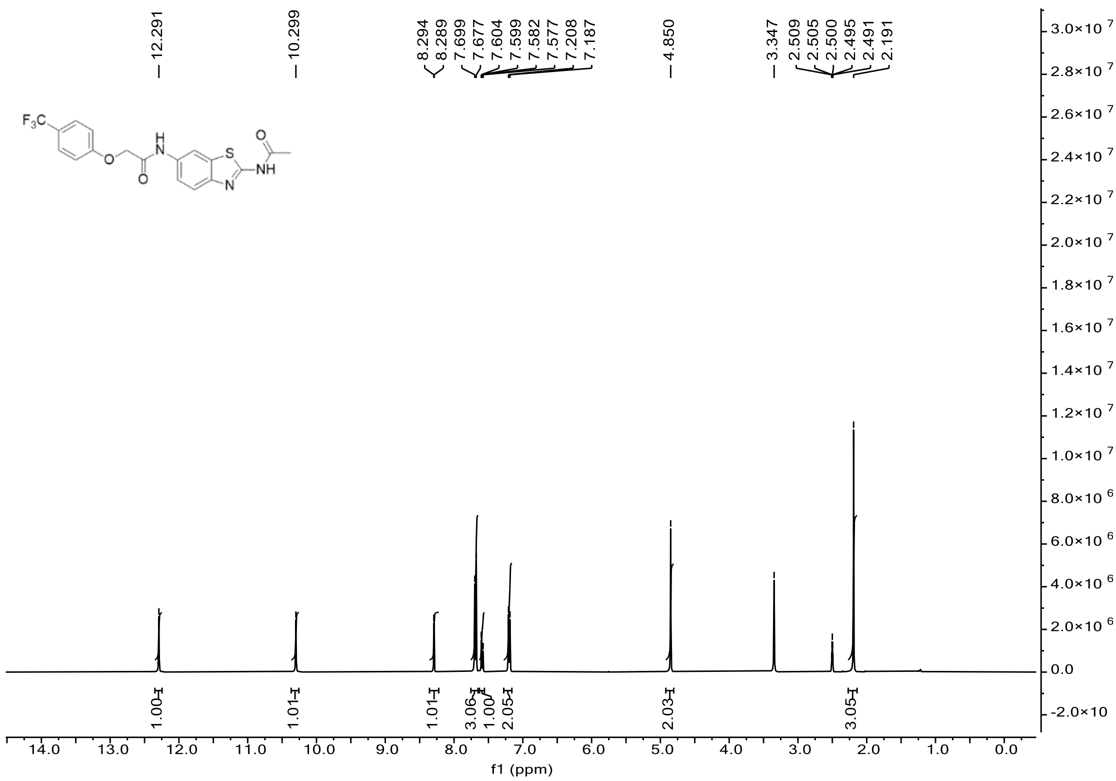

^{13}C NMR of compound **10r** (100 MHz, DMSO-d_6)

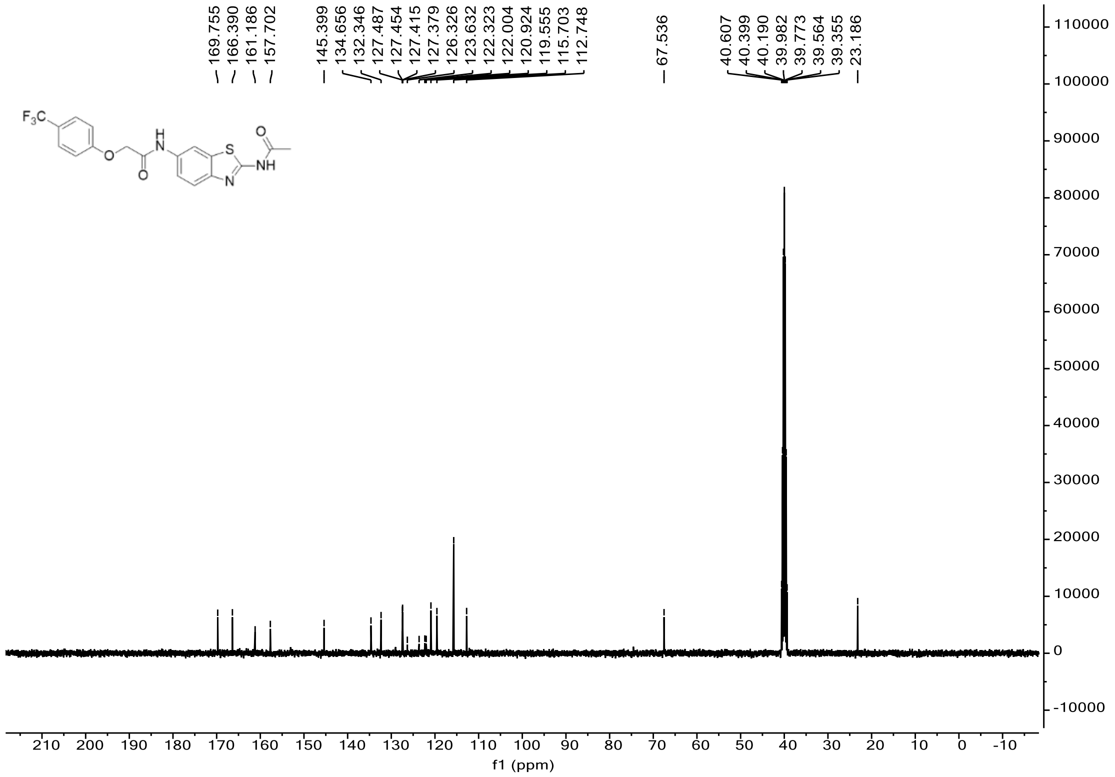

^{19}F NMR of compound **10r** (376 MHz, DMSO-d_6)

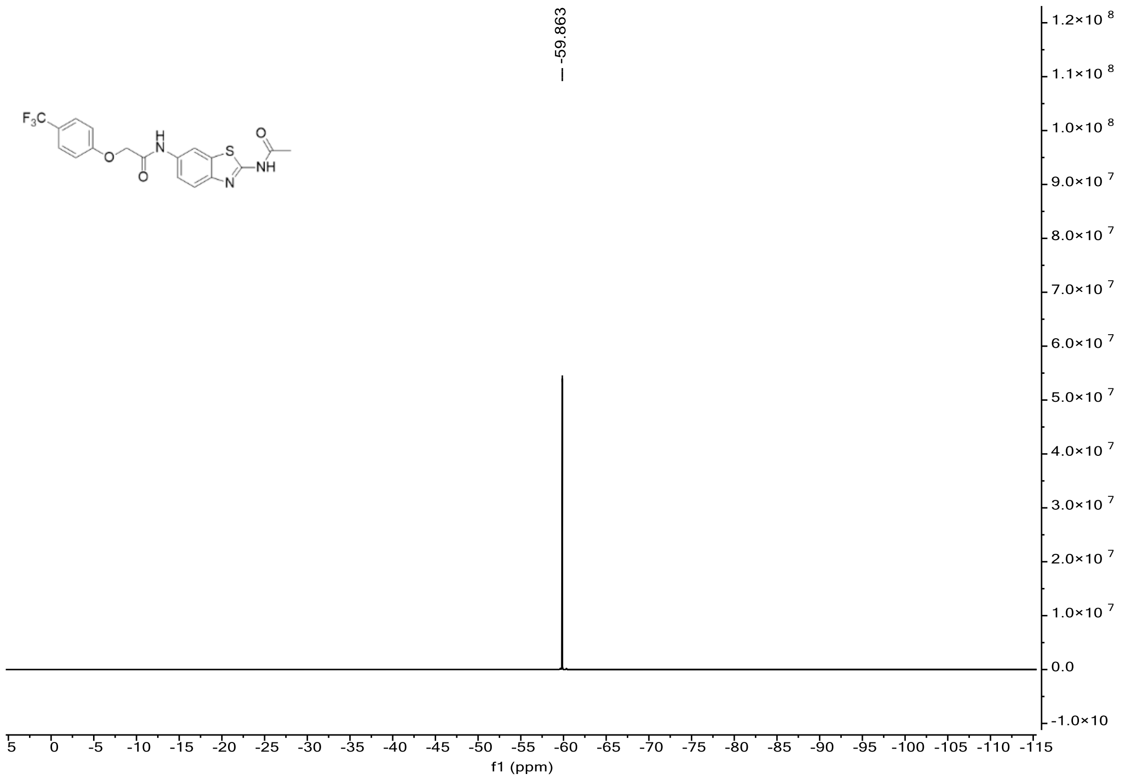

^{1}H NMR of compound **10s** (400 MHz, DMSO-d_6)

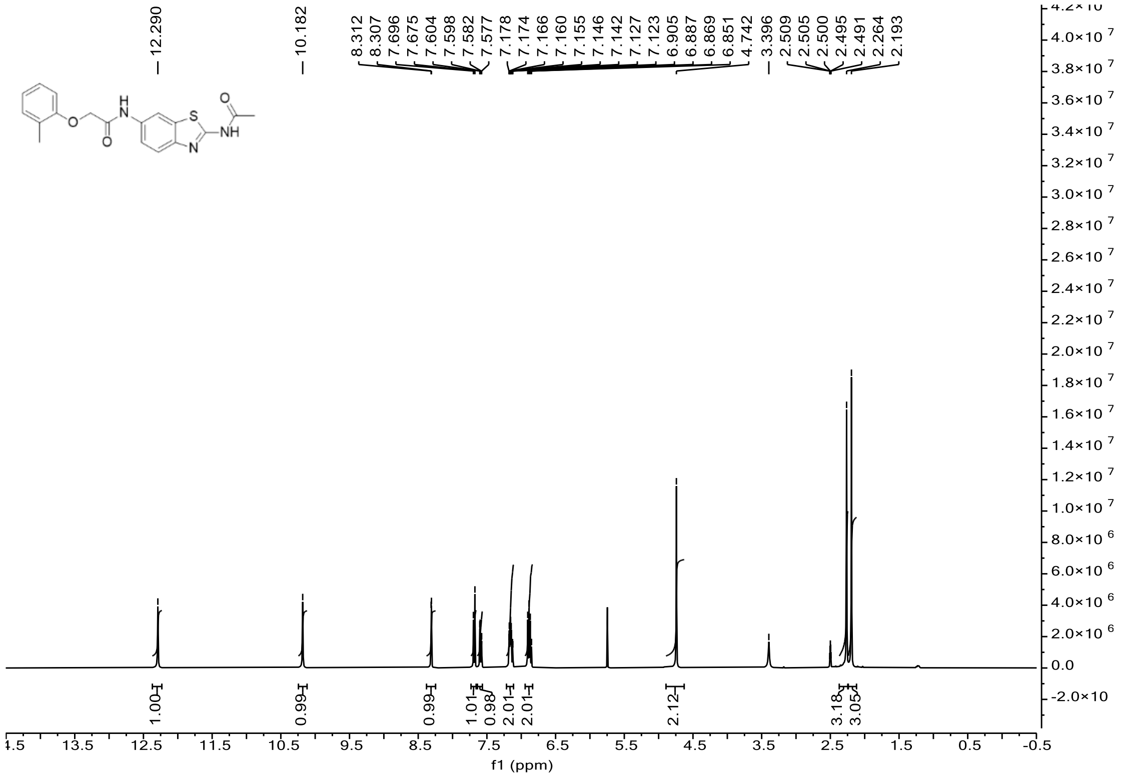

^{13}C NMR of compound **10s** (100 MHz, DMSO-*d*$_6$)

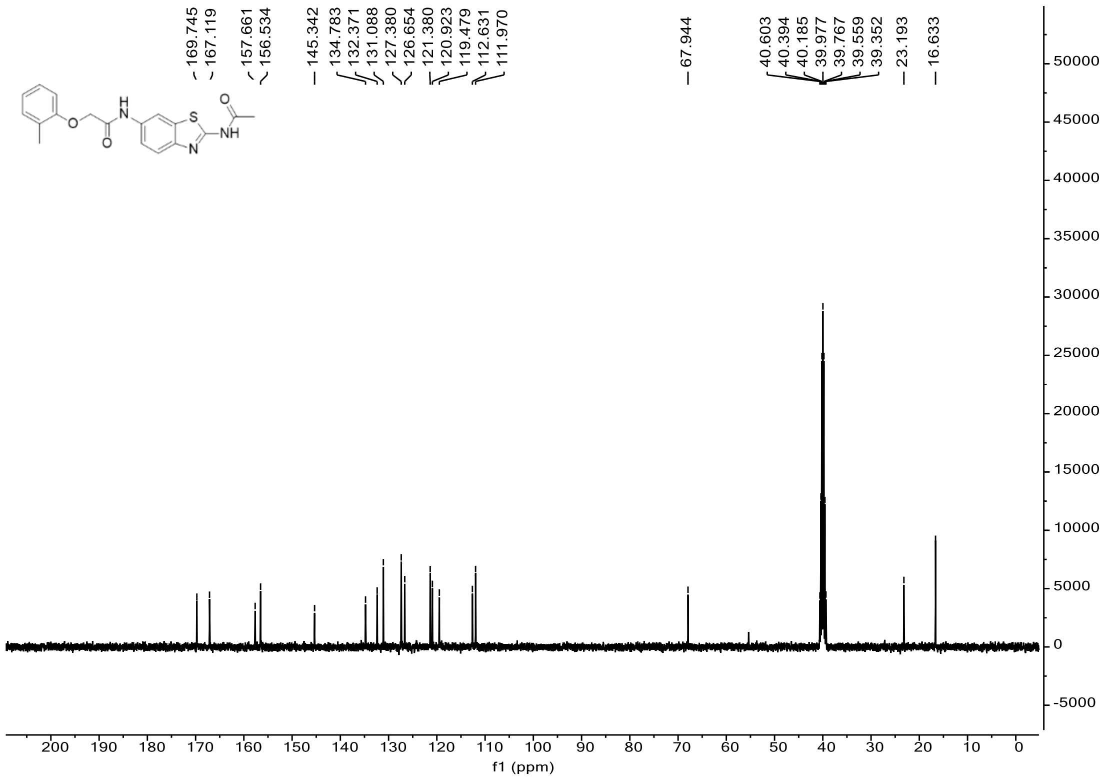

^{1}H NMR of compound **10t** (400 MHz, DMSO-*d*$_6$)

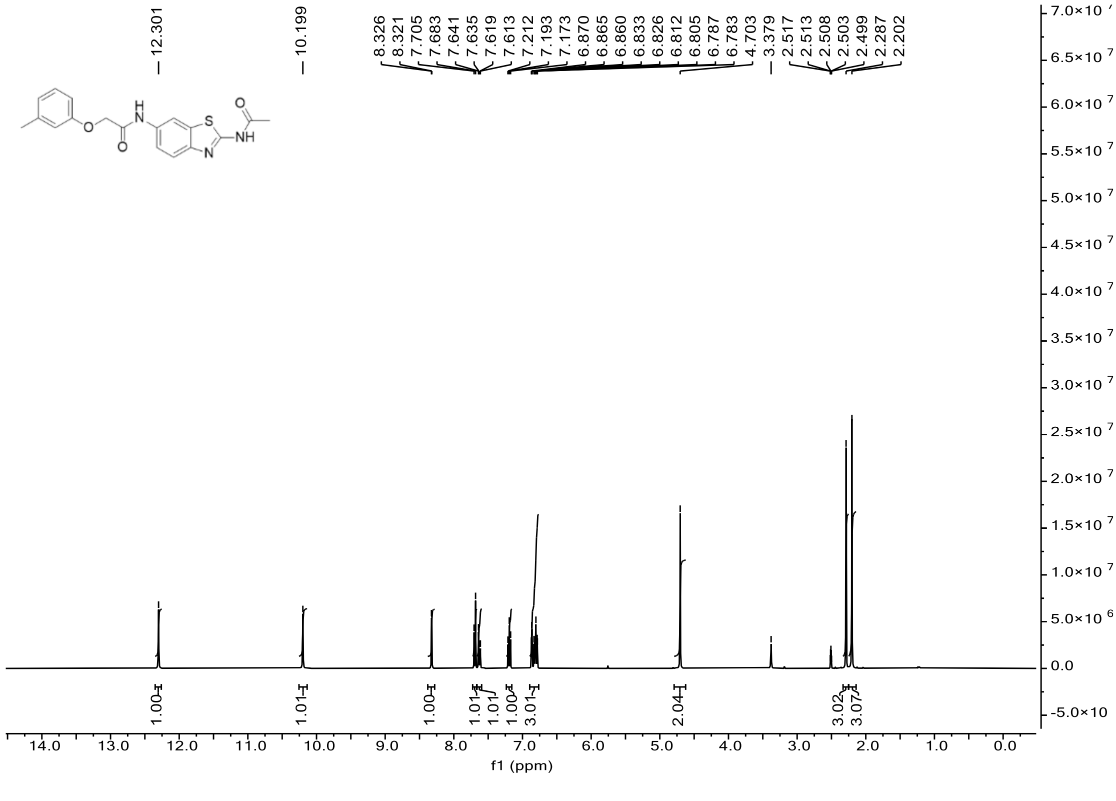

^{13}C NMR of compound **10t** (100 MHz, DMSO-d_6)

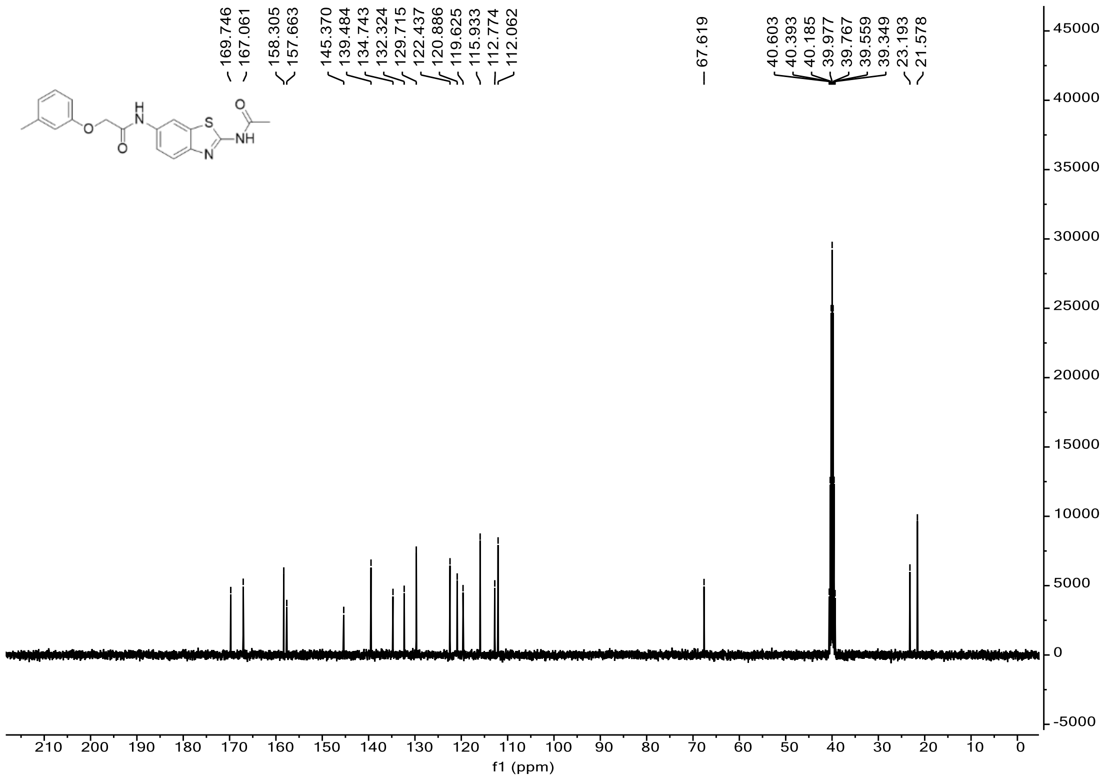

^{1}H NMR of compound **10u** (400 MHz, DMSO-d_6)

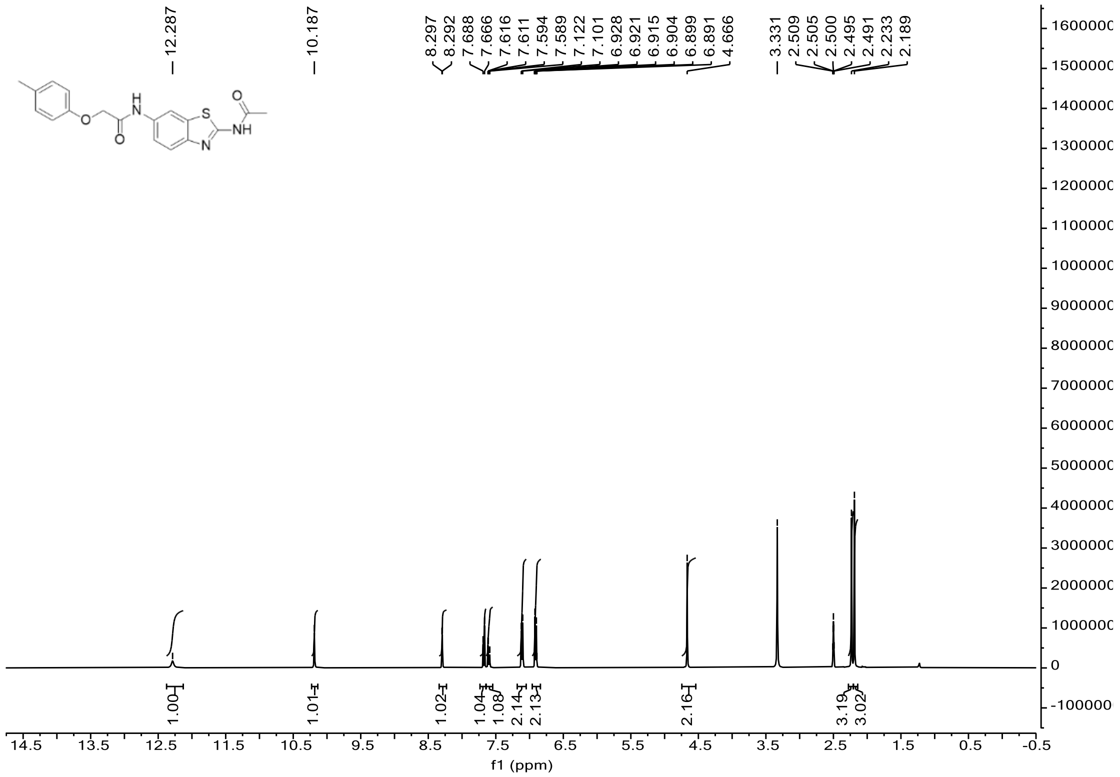

^{13}C NMR of compound **10u** (100 MHz, DMSO-d_6)

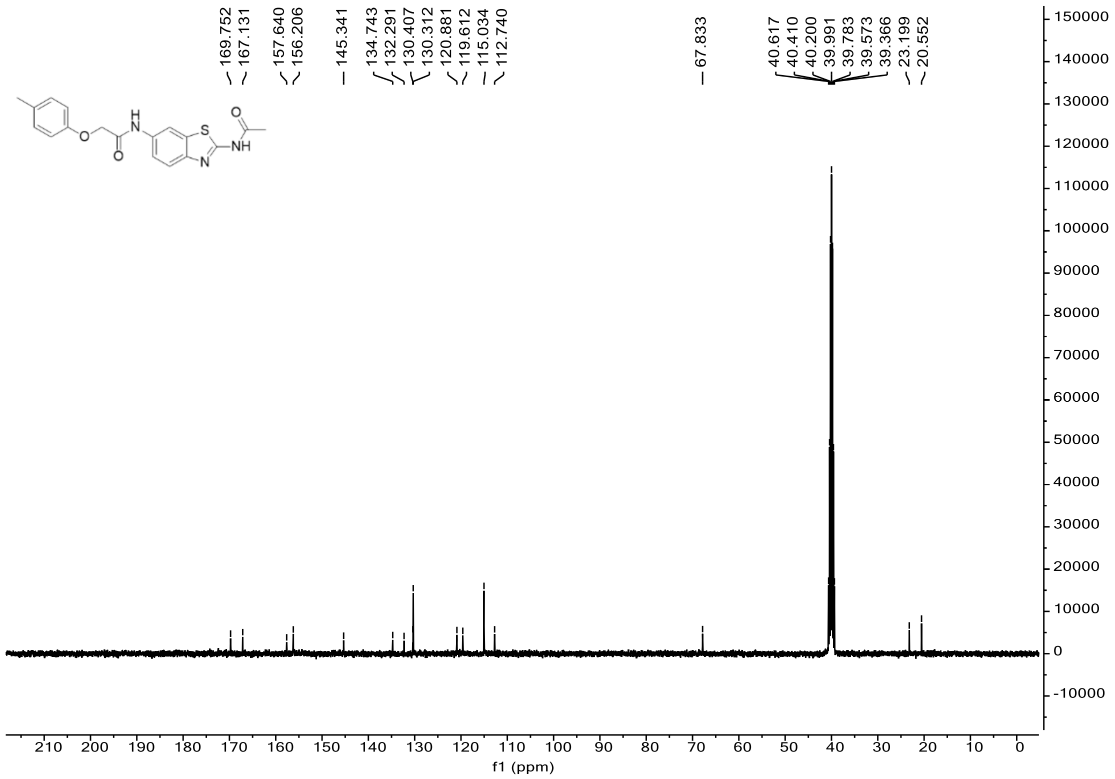

^{1}H NMR of compound **10v** (400 MHz, DMSO-d_6)

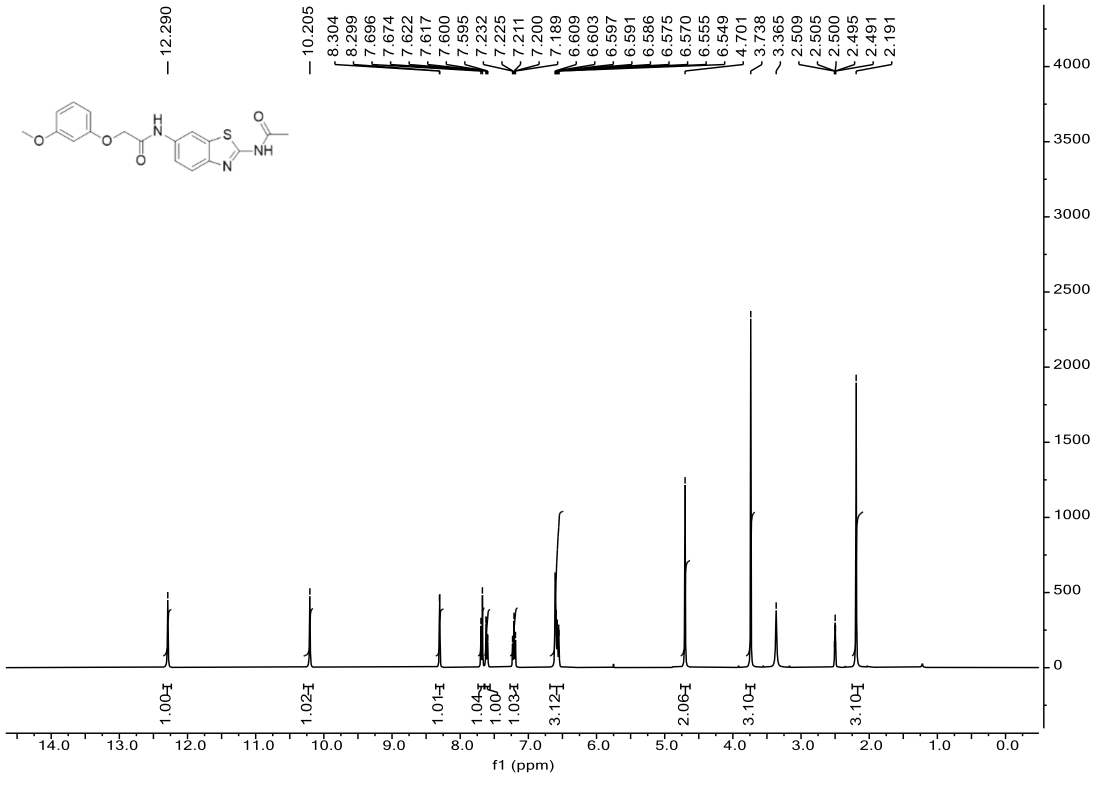

^{13}C NMR of compound **10v** (100 MHz, DMSO-d_6)

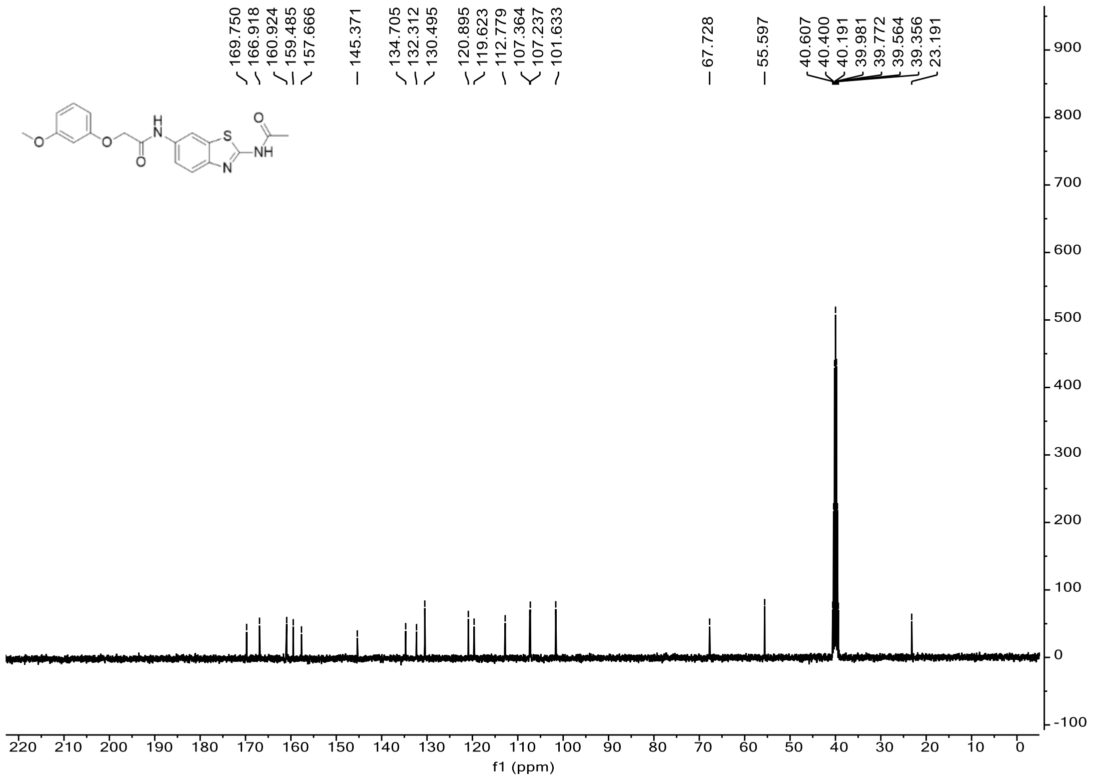

^{1}H NMR of compound **10w** (400 MHz, DMSO-d_6)

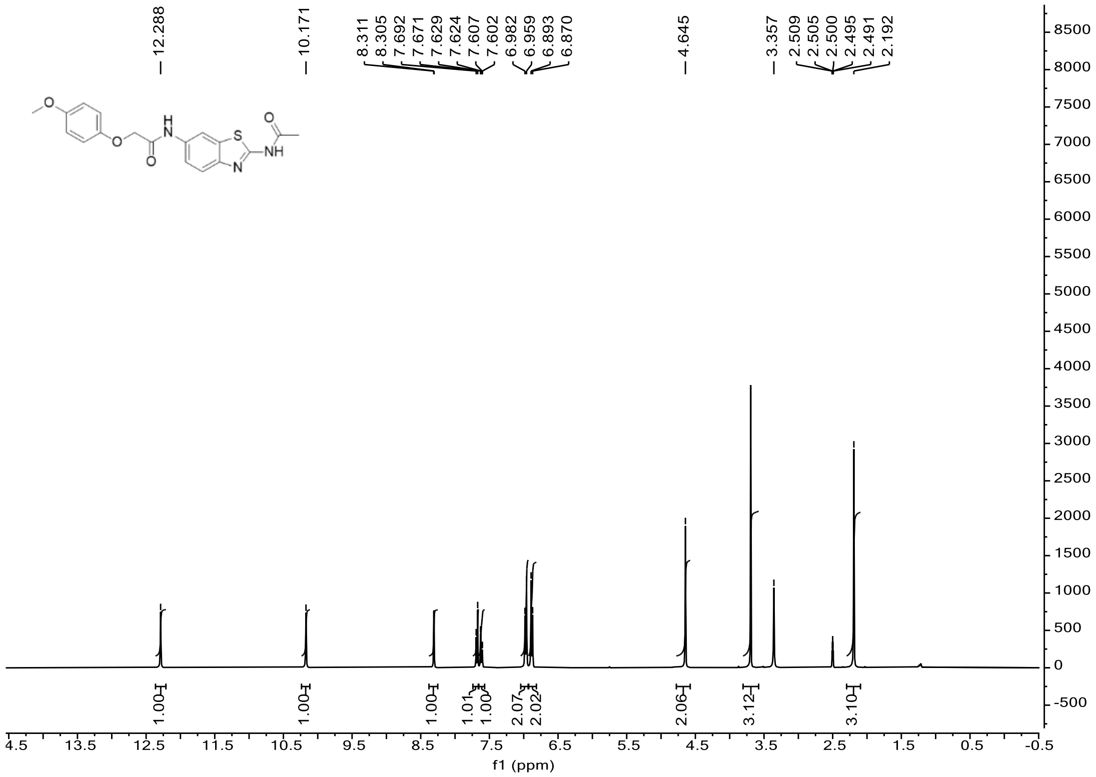

^{13}C NMR of compound **10w** (100 MHz, DMSO-d_6)

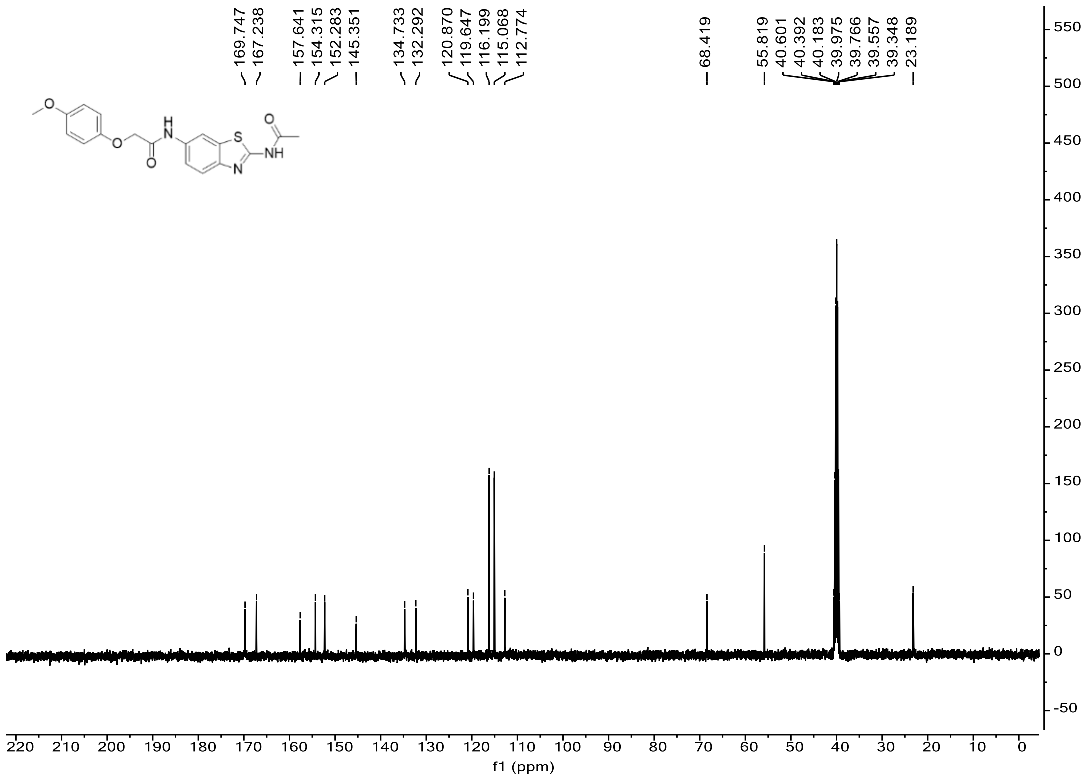

HRMS of compound **10b** (ESI)

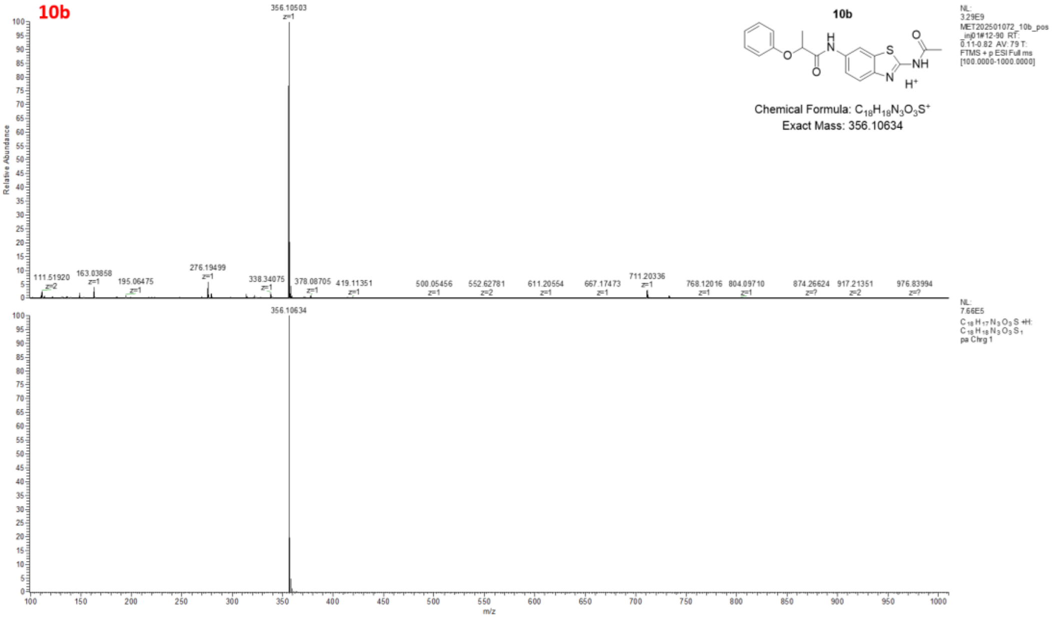

HRMS of compound **10c** (ESI)

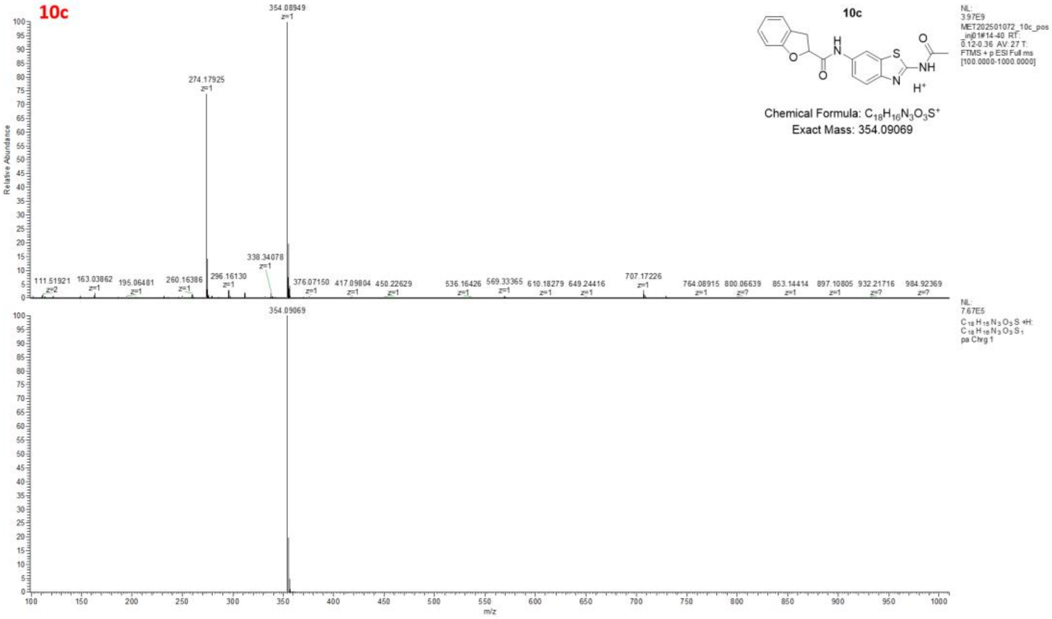

HRMS of compound **10d** (ESI)

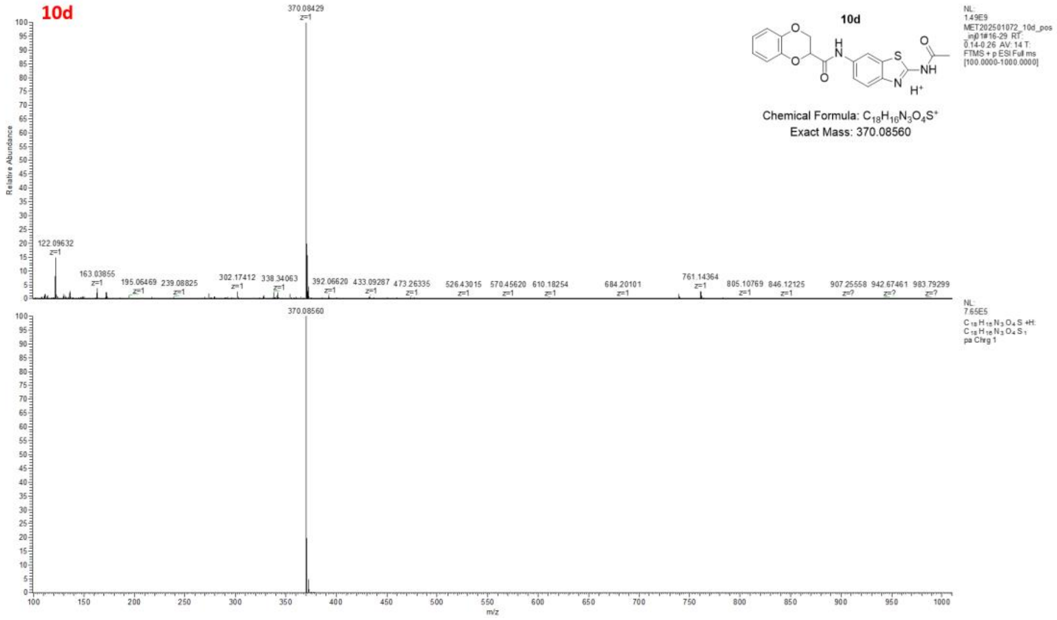

HRMS of compound **10e** (ESI)

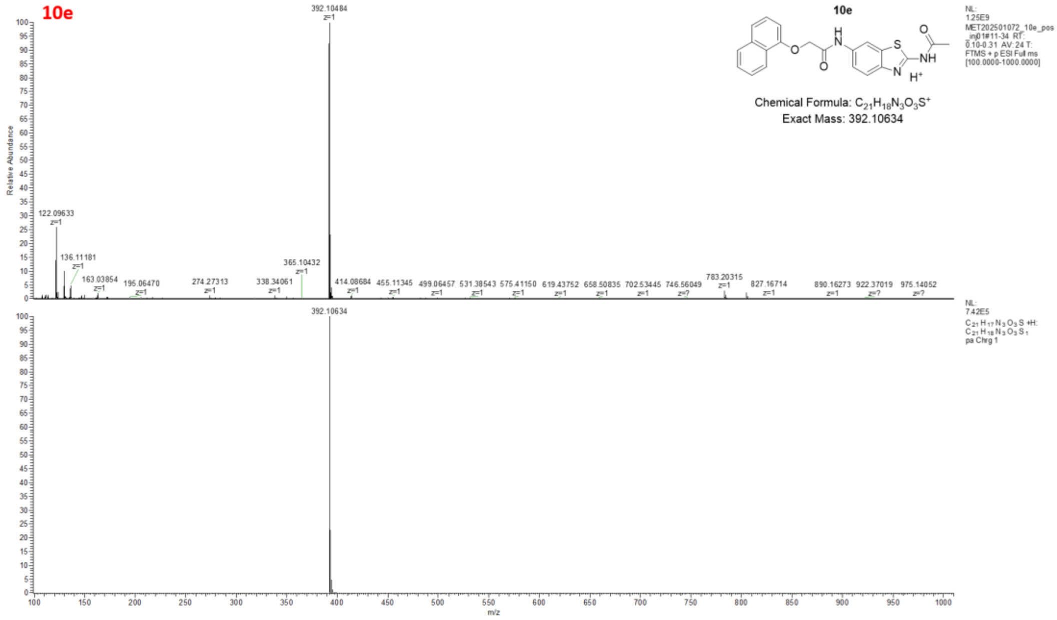

HRMS of compound **10f** (ESI)

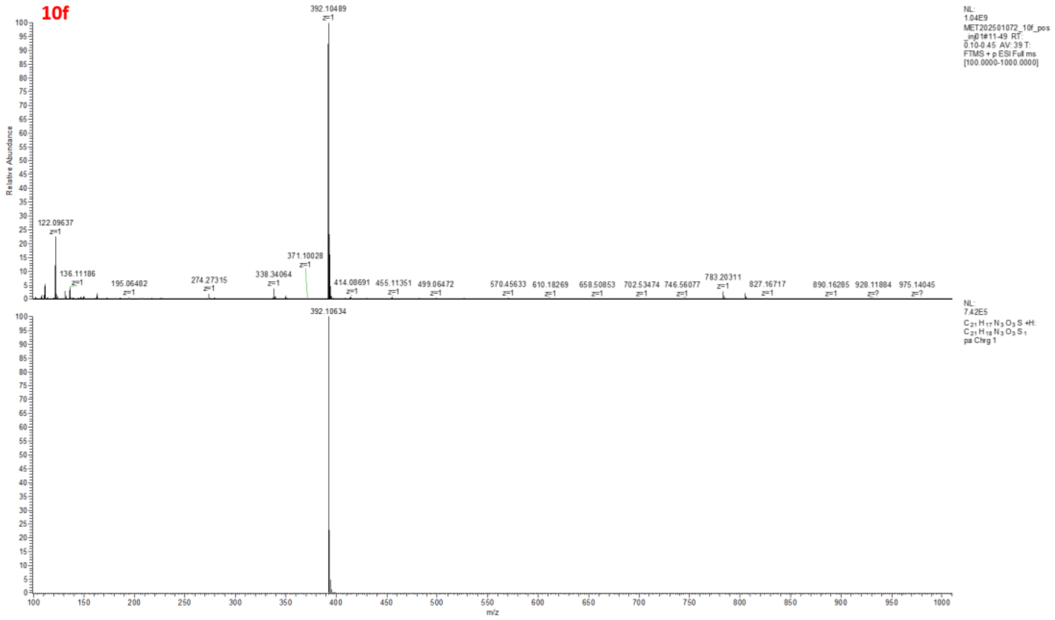

HRMS of compound **10g** (ESI)

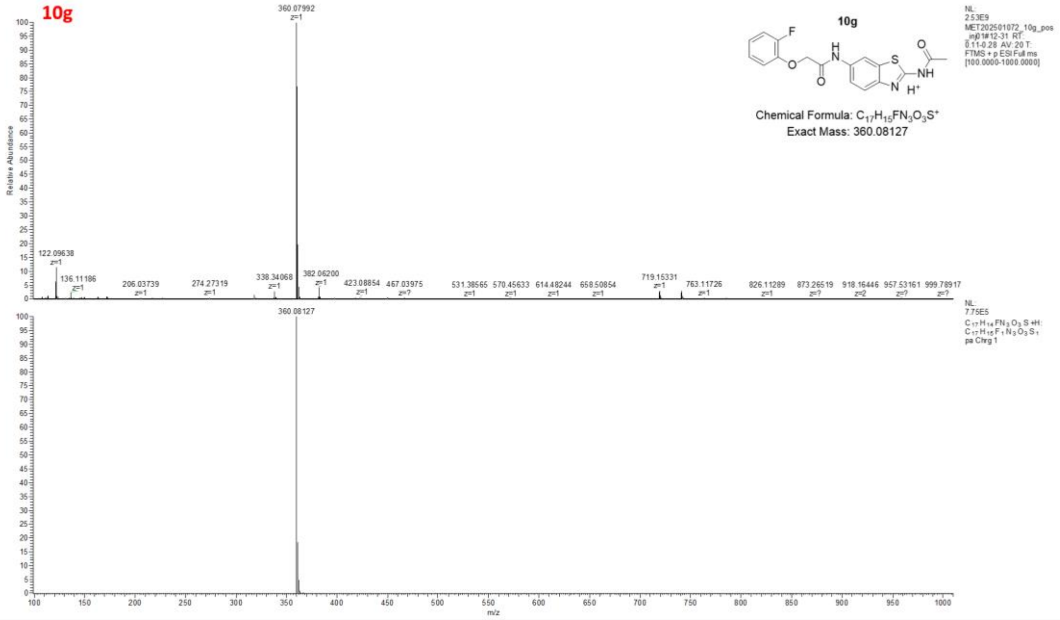

HRMS of compound **10h** (ESI)

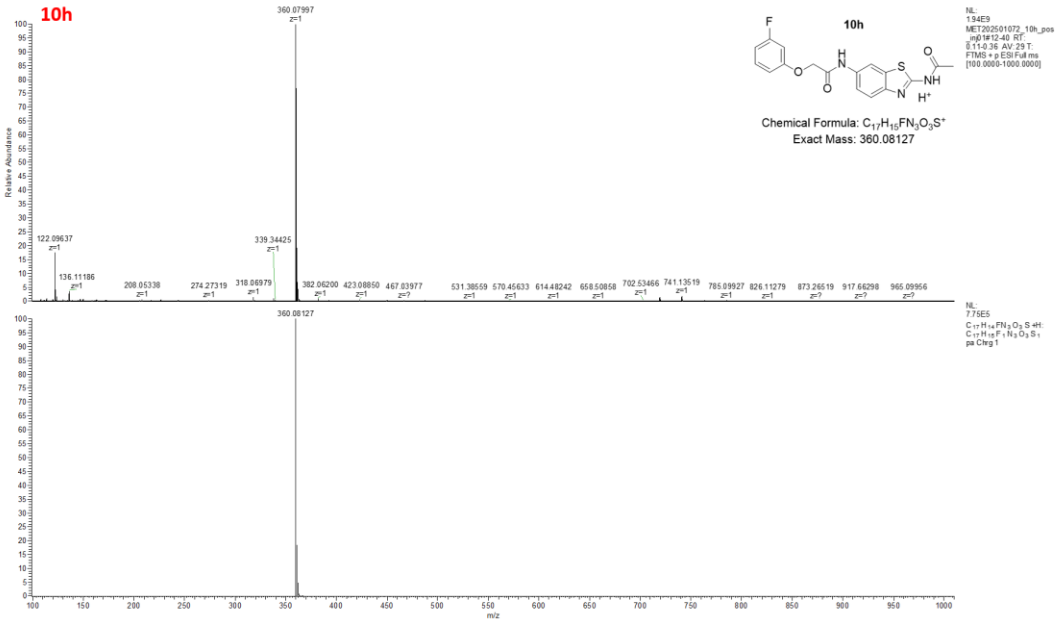

HRMS of compound **10i** (ESI)

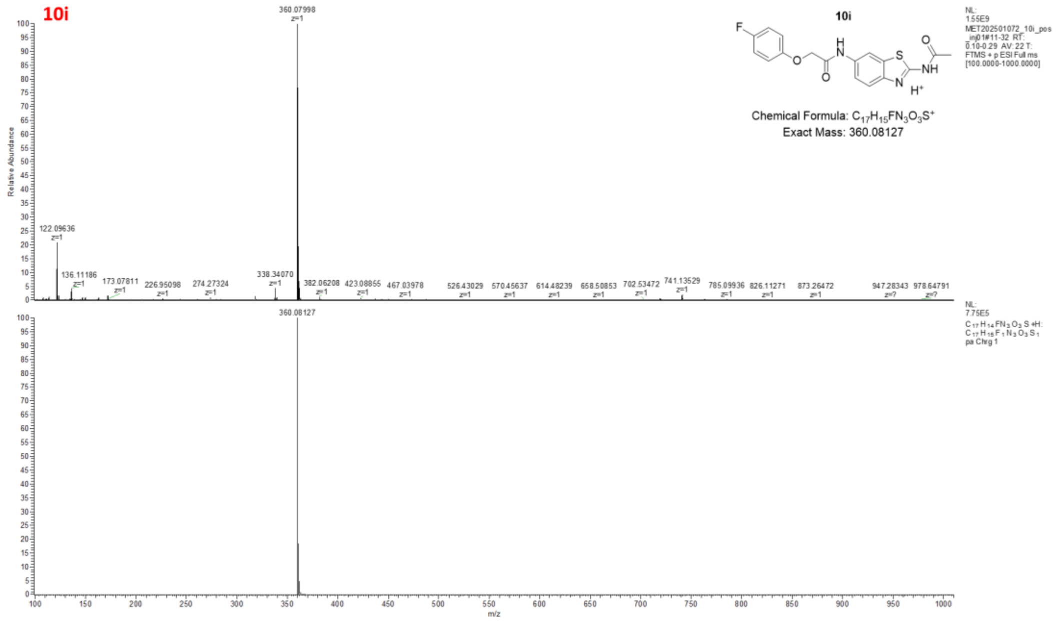

HRMS of compound **10j** (ESI)

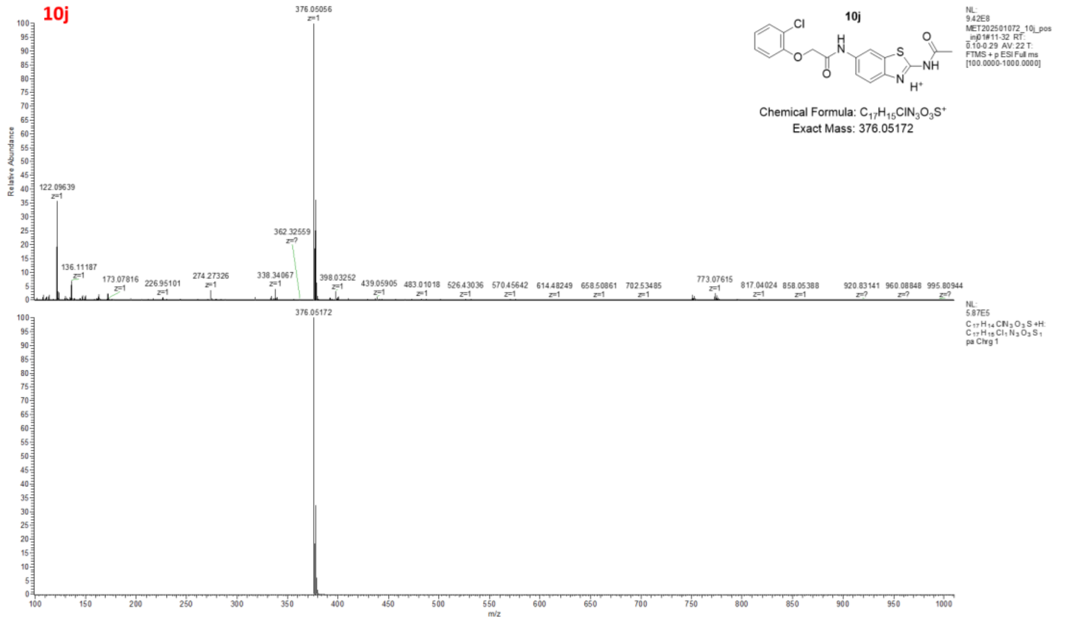

HRMS of compound **10k** (ESI)

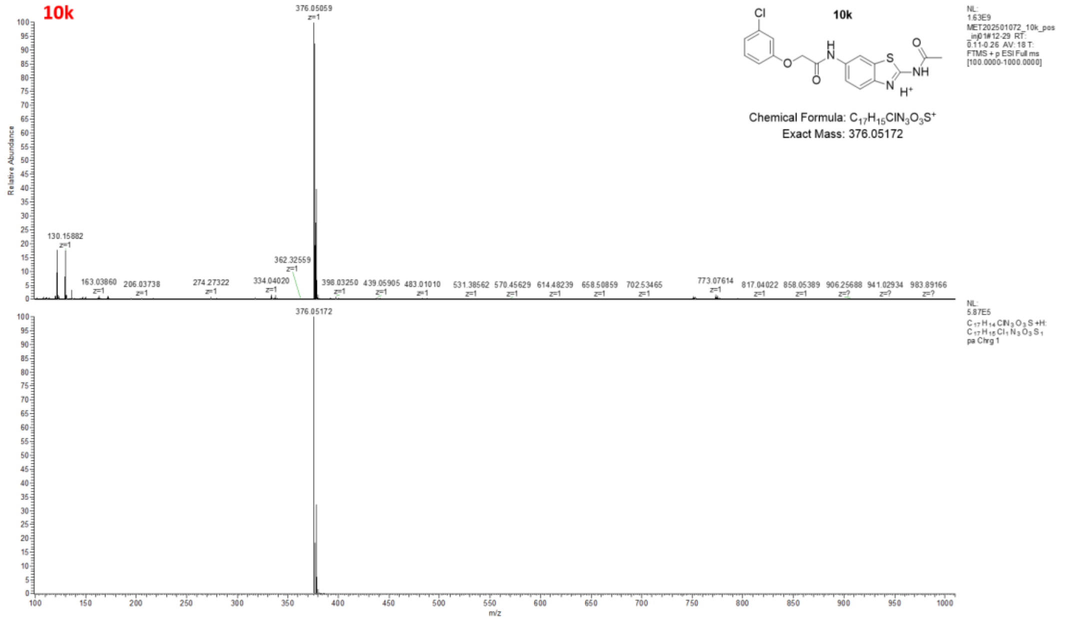

HRMS of compound **10l** (ESI)

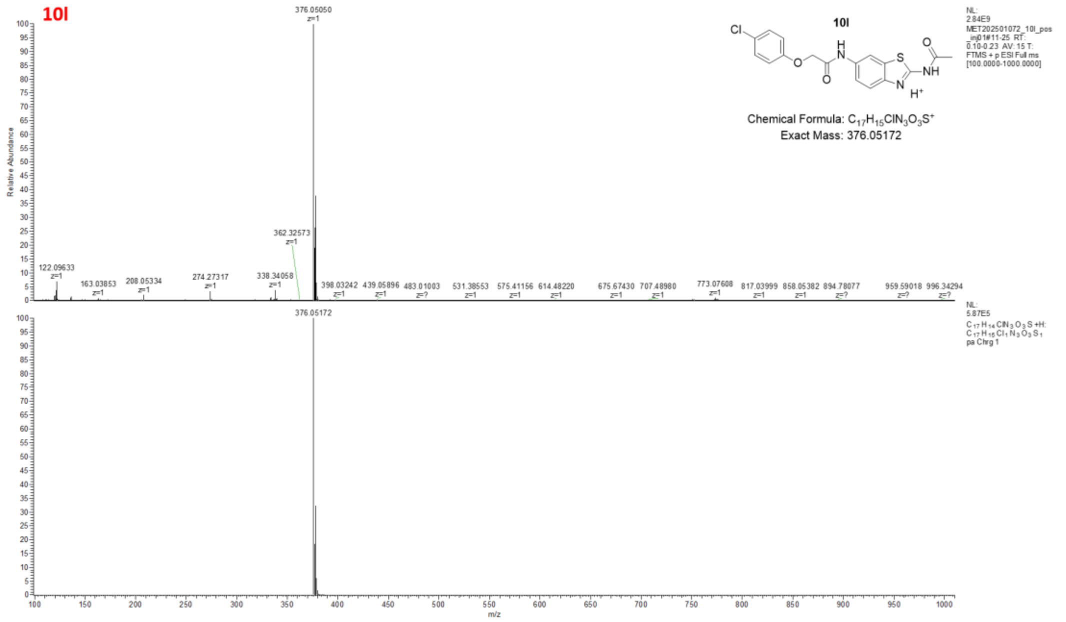

HRMS of compound **10m** (ESI)

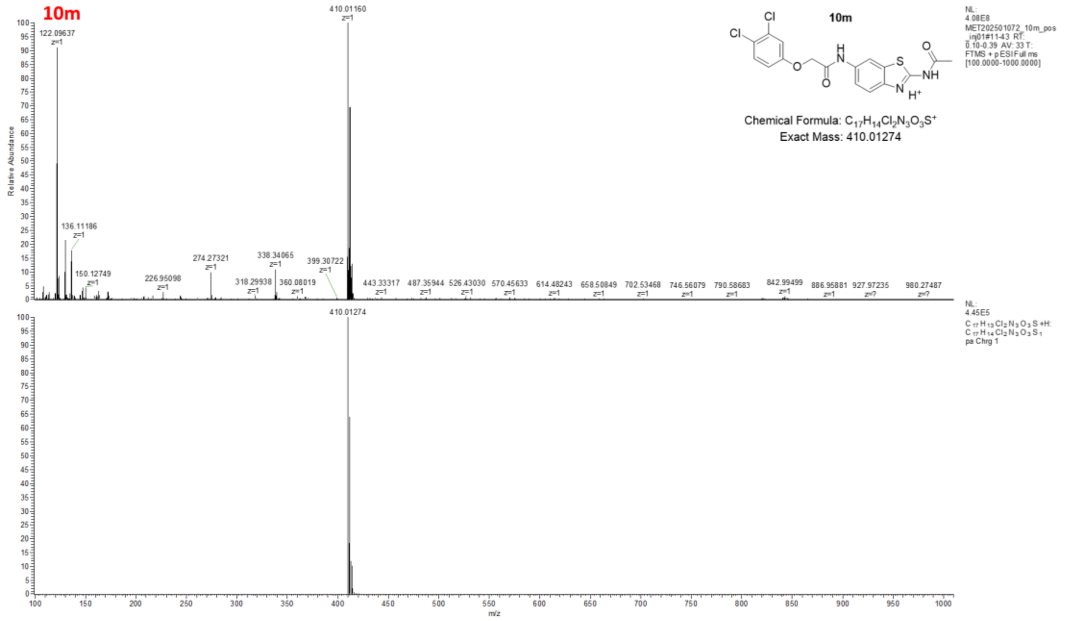

HRMS of compound **10o** (ESI)

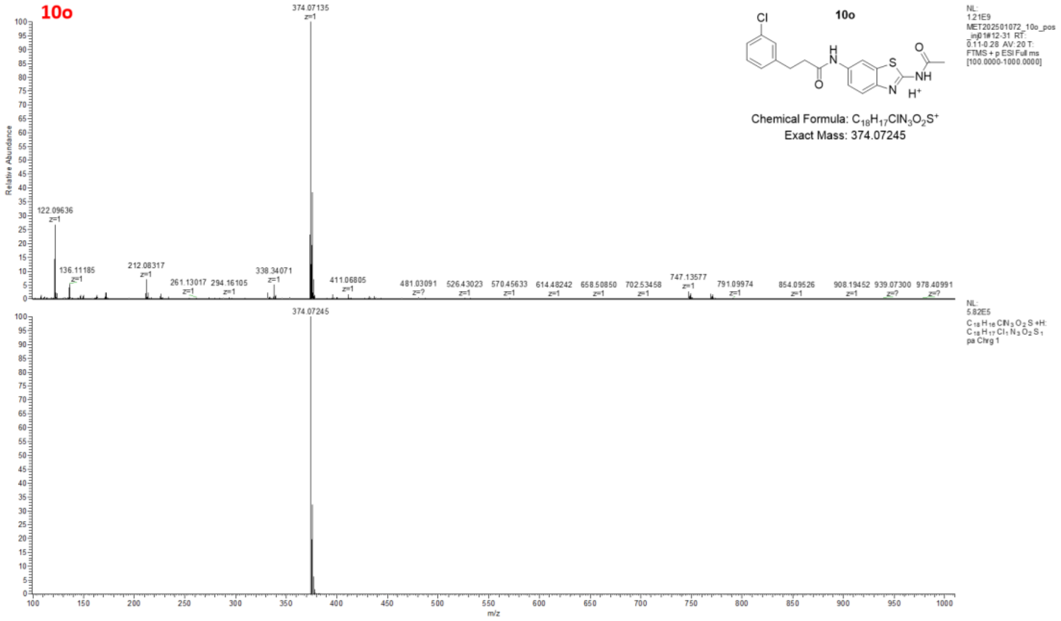

HRMS of compound **10p** (ESI)

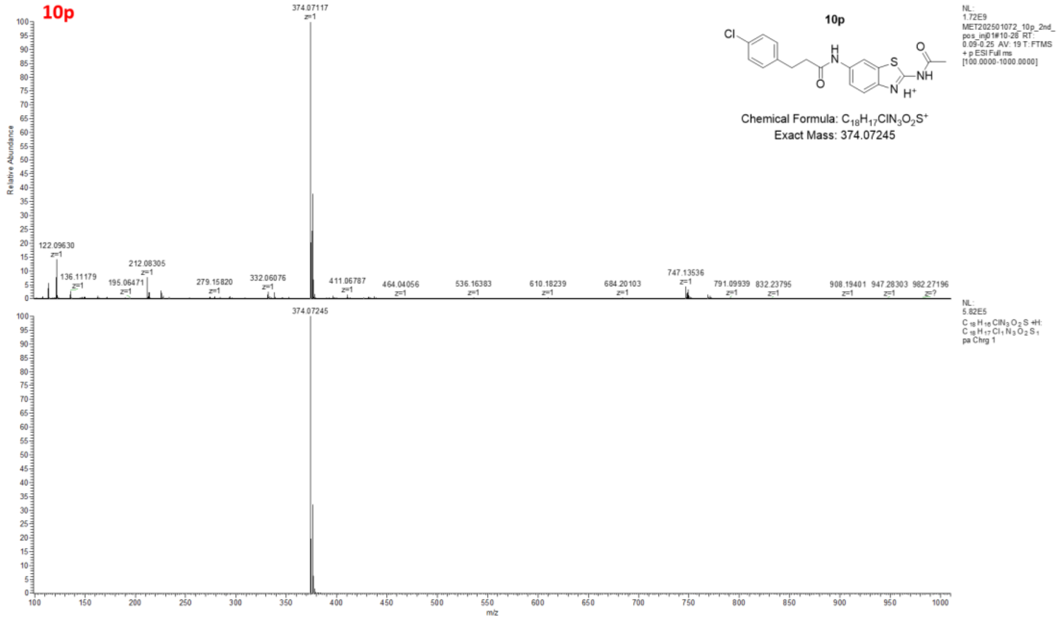

HRMS of compound **10q** (ESI)

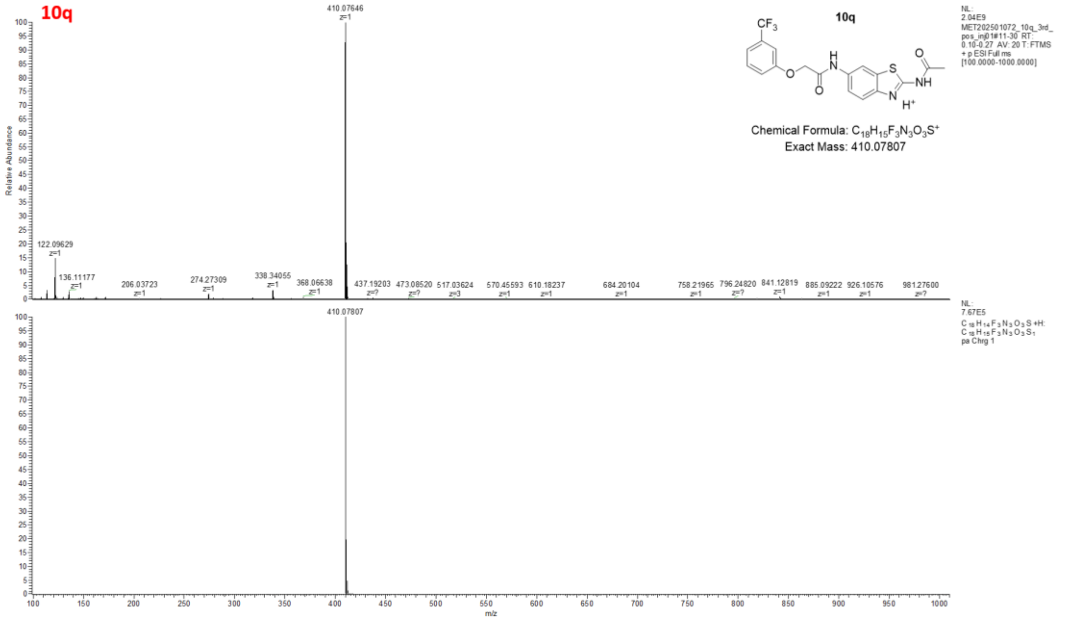

HRMS of compound **10r** (ESI)

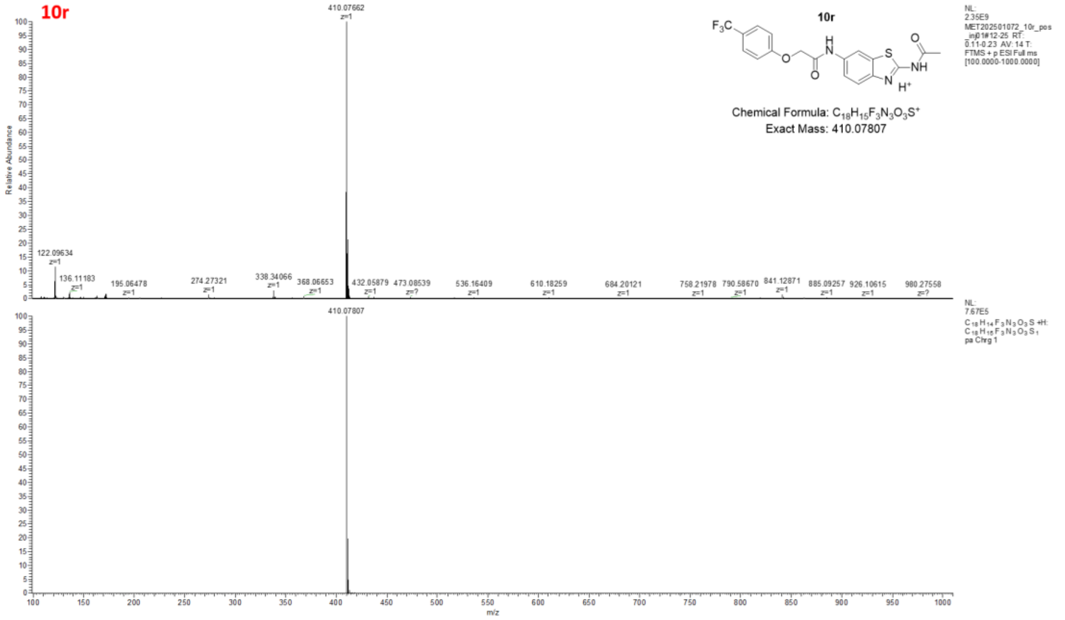

HRMS of compound **10t** (ESI)

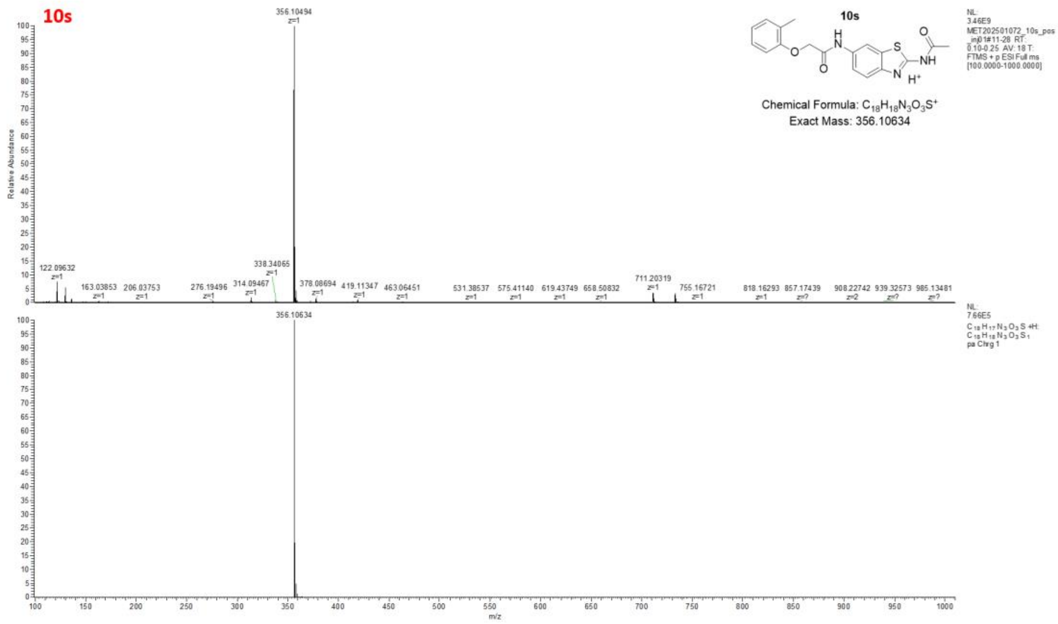

HRMS of compound **10t** (ESI)

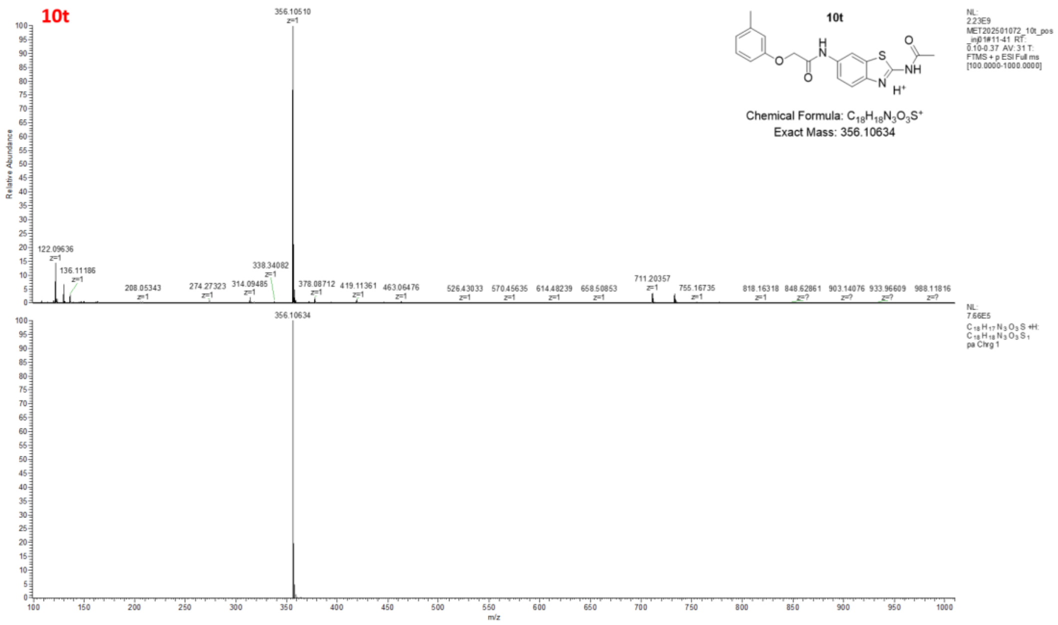

HRMS of compound **10u** (ESI)

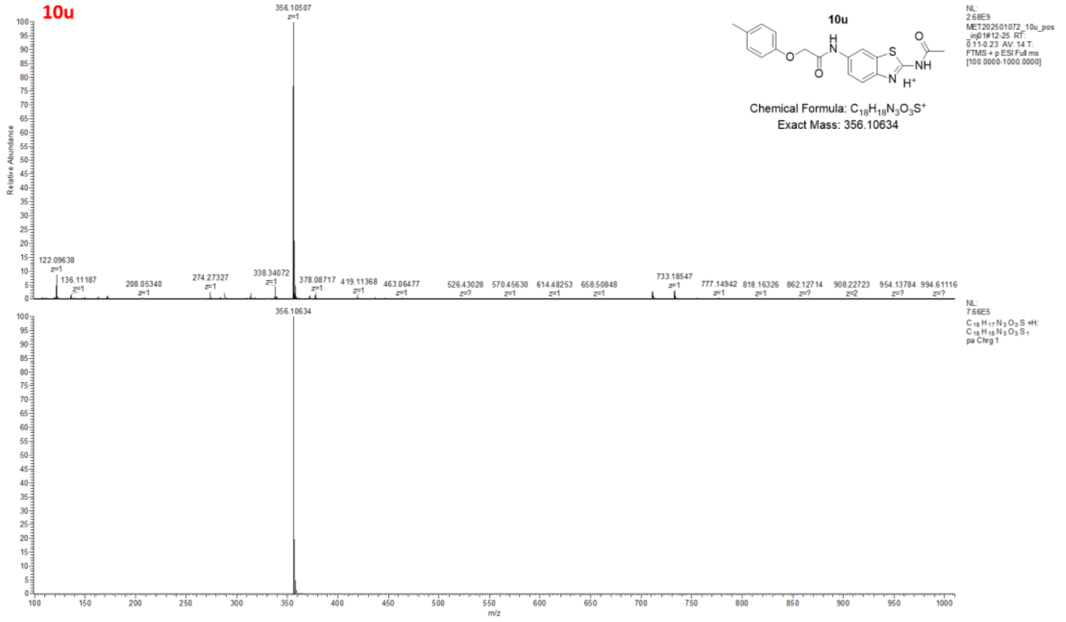

HRMS of compound **10v** (ESI)

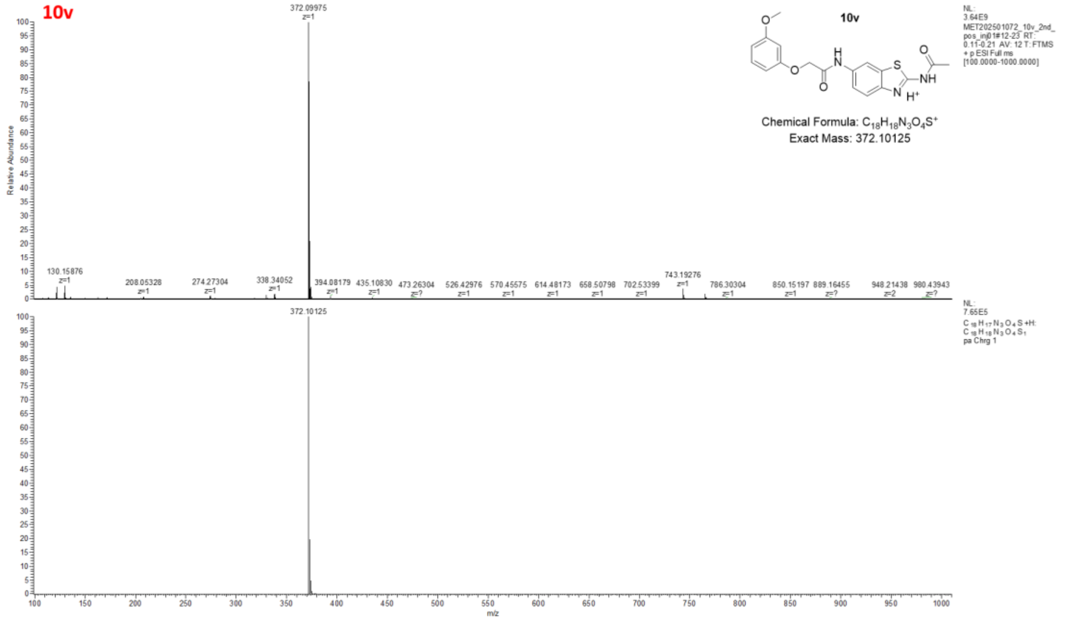

HRMS of compound **10w** (ESI)

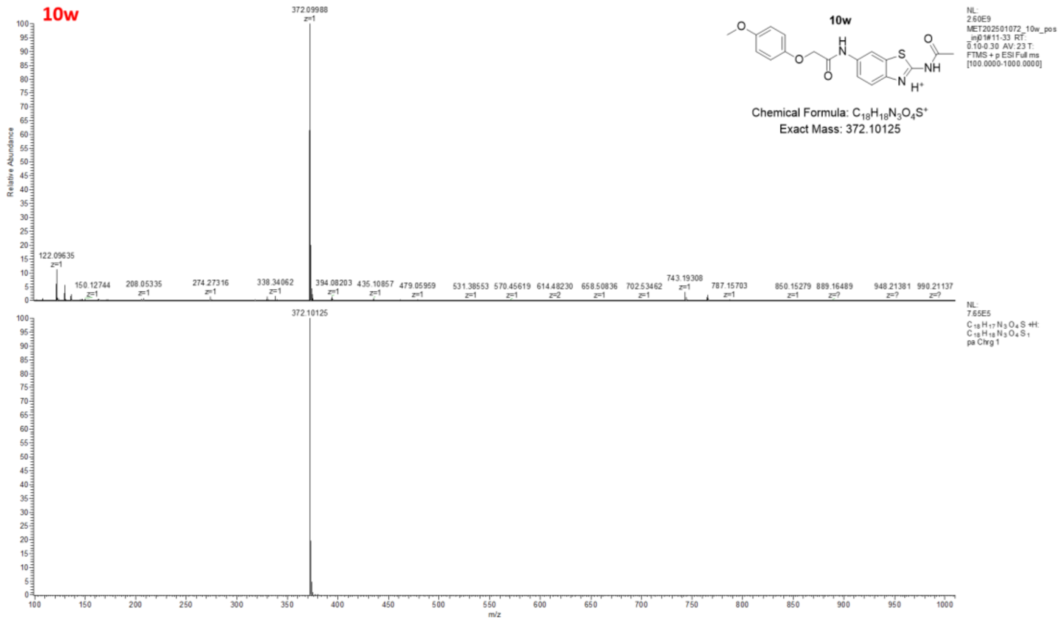

HPLC report annotation

单次进样报告

数据文件Data File: 2024-12-19 03-41-29+08-00-13.dx
序列名称Sequence Name: 1260-2024-12-18 22-26-52+08-00
样品名称Sample Name: ab-p-0
仪器Instrument: 1260
进样日期Date: 2024-12-19 03:42:31+08:00
进样体积Injection Volume: 1.000 µL
位置Location: P1-B5
采集方法Acquisition Method: normal.amx
类型Type: 样品Sample
处理方法Processing Method: *GC_LC 面积百分比_DefaultMethod.pmx
样品含量Sample Content: 1.00
手动修改Manual Modification: 无No

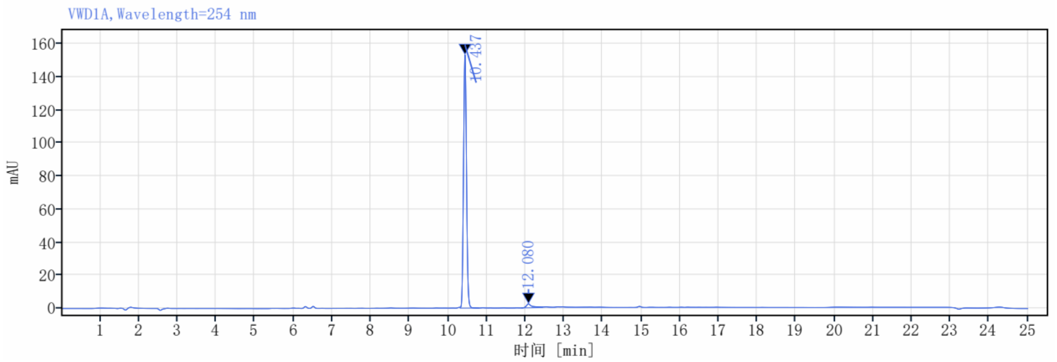

信号Detector: VWD1A,Wavelength=254 nm

保留时间 Retention Time [min]	类型 Type	峰宽 [min] Peak Width	峰面积 Peak Area	峰高 Peak Height	峰面积% Area%	名称
10.437	VV	0.49	828.62	153.20	97.00	
12.080	BV	0.55	25.59	2.38	3.00	
		**总和**	**854.21**			

HPLC purity of compound **10a**

单次进样报告

Agilent

**数据文件:**	2024-12-23 12-16-16+08-00-03.dx		
**序列名称:**	1260-2024-12-23 11-23-42+08-00		
**样品名称:**	ab-p-3		
**仪器:**	1260	**进样日期:**	2024-12-23 12:17:19+08:00
**进样体积:**	5.000 µL	**位置:**	P1-B7
**采集方法:**	normal.amx	**类型:**	样品
**处理方法:**	*Checkout.pmx	**样品含量:**	0.00
**手动修改:**	手动积分		

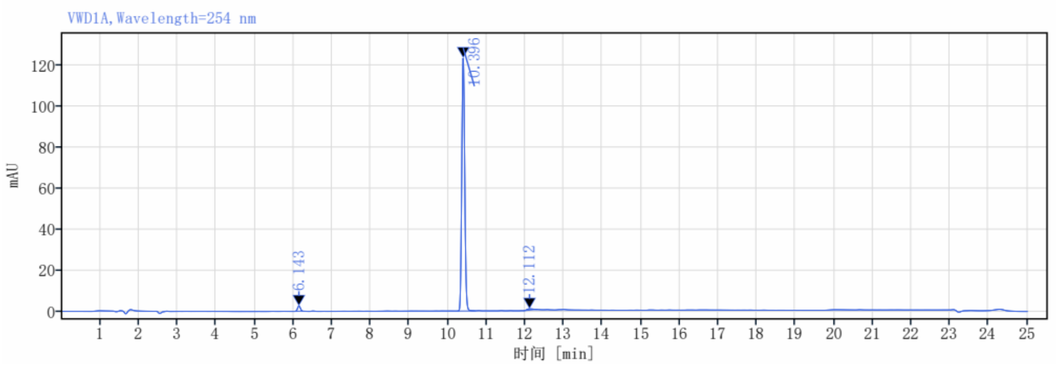

信号: VWD1A, Wavelength=254 nm

保留时间 [min]	类型	峰宽 [min]	峰面积	峰高	峰面积%	名称
6.143	MM m	0.19	12.78	2.53	1.88	
10.396	BV	0.46	661.48	123.66	97.11	
12.112	MM m	0.35	6.90	0.65	1.01	
		**总和**	**681.16**			

HPLC purity of compound **10b**

单次进样报告

Agilent

**数据文件:**	2024-12-04 01-21-05+08-00-08.dx		
**序列名称:**	1260-2024-12-03 22-17-36+08-00		
**样品名称:**	ab-p-1		
**仪器:**	1260	**进样日期:**	2024-12-04 01:22:06+08:00
**进样体积:**	1.000 µL	**位置:**	P1-A4
**采集方法:**	normal.amx	**类型:**	样品
**处理方法:**	*GC_LC<br>面积百分比_DefaultMethod.pmx	**样品含量:**	0.00
**手动修改:**	无		

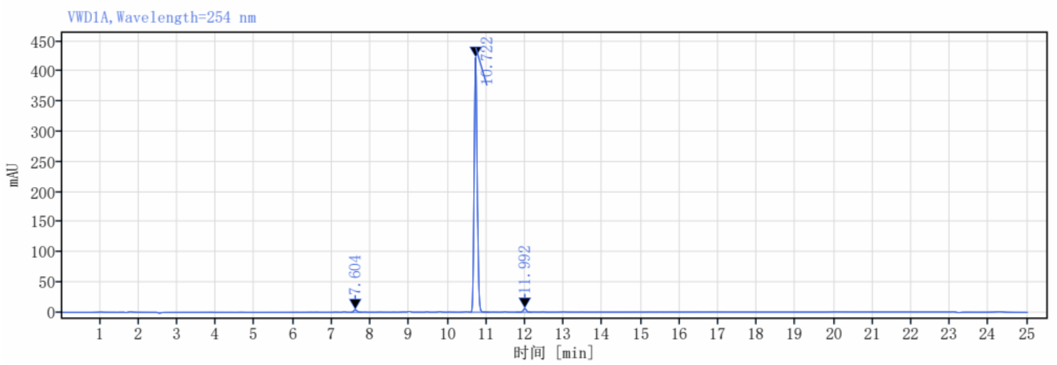

信号: VWD1A,Wavelength=254 nm

**保留时间 [min]**	**类型**	**峰宽 [min]**	**峰面积**	**峰高**	**峰面积%**	**名称**
7.604	BV	0.28	23.91	4.11	1.03	
10.722	VV	0.49	2258.89	422.80	97.47	
11.992	BV	0.40	34.69	5.98	1.50	
		**总和**	**2317.49**			

HPLC purity of compound **10c**

单次进样报告

**数据文件:**	2025-01-06 16-33-16+08-00-59.dx		
**序列名称:**	1260-2025-01-05 20-56-54+08-00		
**样品名称:**	ab-p1		
**仪器:**	1260	**进样日期:**	2025-01-06 16:34:23+08:00
**进样体积:**	1.000 µL	**位置:**	P1-F5
**采集方法:**	normal.amx	**类型:**	样品
**处理方法:**	Checkout.pmx	**样品含量:**	0.00
**手动修改:**	无		

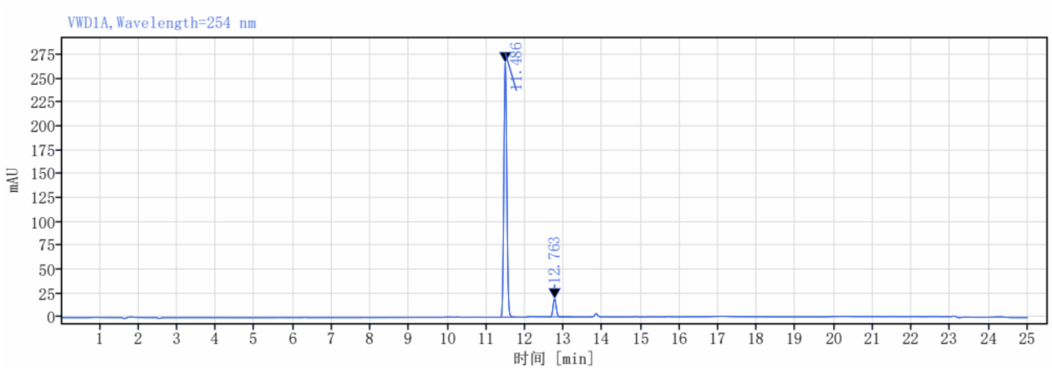

信号: VWD1A, Wavelength=254 nm

保留时间 [min]	类型	峰宽 [min]	峰面积	峰高	峰面积%	名称
11.486	BB	0.58	1423.61	266.08	93.39	
12.763	BB	0.61	100.80	18.50	6.61	
		**总和**	**1524.41**			

HPLC purity of compound **10d**

单次进样报告

**数据文件:**	2024-12-23 13-08-38+08-00-05.dx		
**序列名称:**	1260-2024-12-23 11-23-42+08-00		
**样品名称:**	ab-p-4		
**仪器:**	1260	**进样日期:**	2024-12-23 13:09:40+08:00
**进样体积:**	5.000 µL	**位置:**	P1-B8
**采集方法:**	normal.amx	**类型:**	样品
**处理方法:**	*Checkout.pmx	**样品含量:**	0.00
**手动修改:**	手动积分		

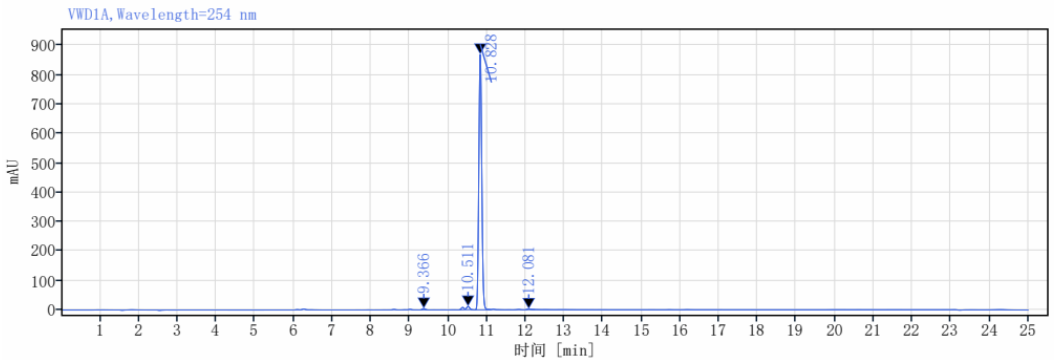

信号: VWD1A, Wavelength=254 nm

保留时间 [min]	类型	峰宽 [min]	峰面积	峰高	峰面积%	名称
9.366	MM m	0.16	21.01	4.37	0.44	
10.511	MM m	0.34	101.05	10.97	2.10	
10.828	BV	0.41	4683.87	870.02	97.21	
12.081	MM m	0.24	12.40	2.16	0.26	
		**总和**	**4818.33**			

HPLC purity of compound **10e**

单次进样报告

Agilent

**数据文件:**	2024-12-23 14-53-21+08-00-09.dx		
**序列名称:**	1260-2024-12-23 11-23-42+08-00		
**样品名称:**	ab-p-6		
**仪器:**	1260	**进样日期:**	2024-12-23 14:54:25+08:00
**进样体积:**	5.000 µL	**位置:**	P1-B10
**采集方法:**	normal.amx	**类型:**	样品
**处理方法:**	*Checkout.pmx	**样品含量:**	0.00
**手动修改:**	手动积分		

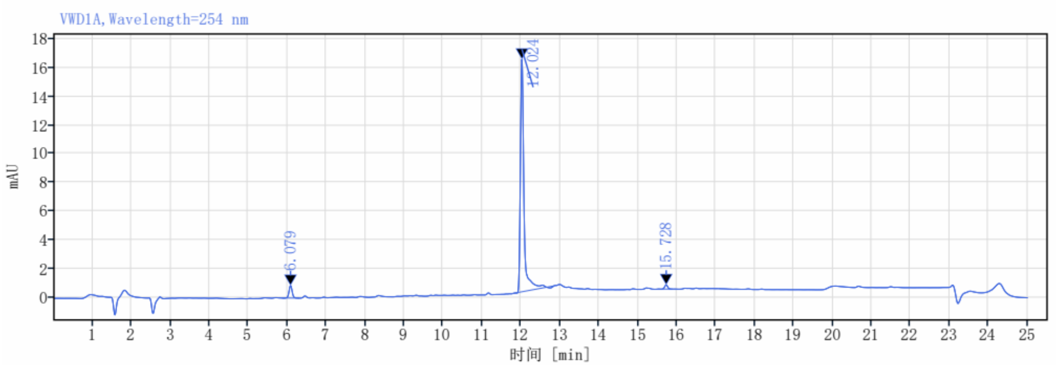

信号: VWD1A, Wavelength=254 nm

保留时间 [min]	类型	峰宽 [min]	峰面积	峰高	峰面积%	名称
6.079	MM m	0.44	4.88	0.86	4.47	
12.024	MM m	1.05	102.78	16.14	94.14	
15.728	MM m	0.36	1.52	0.29	1.39	
		**总和**	**109.18**			

HPLC purity of compound **10f**

单次进样报告

**数据文件:**	2024-12-23 14-01-00+08-00-07.dx		
**序列名称:**	1260-2024-12-23 11-23-42+08-00		
**样品名称:**	ab-p-5		
**仪器:**	1260	**进样日期:**	2024-12-23 14:02:00+08:00
**进样体积:**	5.000 µL	**位置:**	P1-B9
**采集方法:**	normal.amx	**类型:**	样品
**处理方法:**	*Checkout.pmx	**样品含量:**	0.00
**手动修改:**	手动积分		

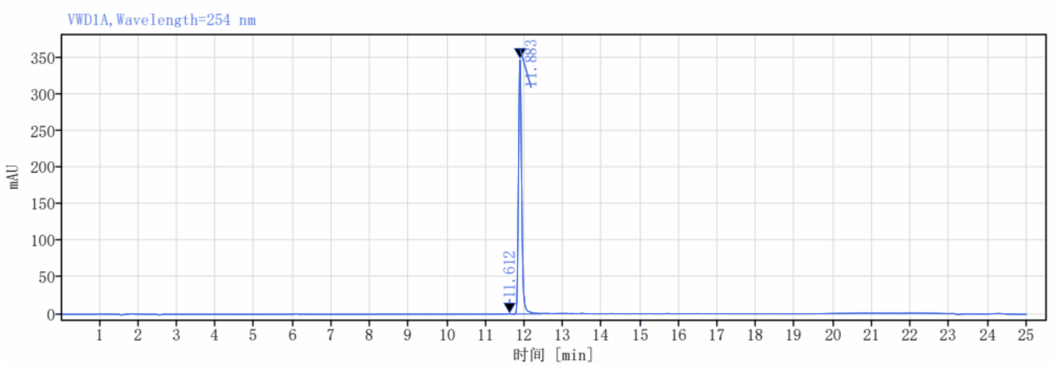

信号: VWD1A, Wavelength=254 nm

保留时间 [min]	类型	峰宽 [min]	峰面积	峰高	峰面积%	名称
11.612	MM m	0.21	2.13	0.39	0.11	
11.883	BV	0.69	2017.26	347.62	99.89	
		**总和**	**2019.39**			

HPLC purity of compound **10g**

单次进样报告

**数据文件:**	2024-12-27 23-57-17+08-00-05.dx		
**序列名称:**	1260-2024-12-27 22-12-18+08-00		
**样品名称:**	ab-p-9		
**仪器:**	1260	**进样日期:**	2024-12-27 23:58:20+08:00
**进样体积:**	5.000 µL	**位置:**	P1-C2
**采集方法:**	normal.amx	**类型:**	样品
**处理方法:**	*Checkout.pmx	**样品含量:**	0.00
**手动修改:**	手动积分		

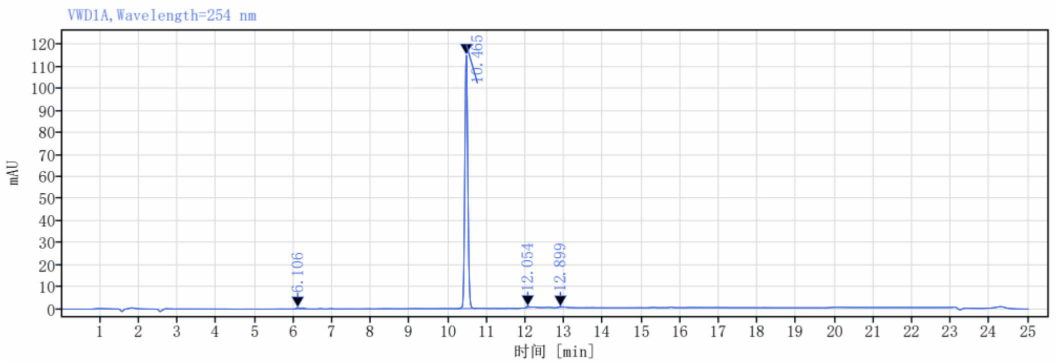

信号: VWD1A,Wavelength=254 nm

保留时间 [min]	类型	峰宽 [min]	峰面积	峰高	峰面积%	名称
6.106	MM m	0.28	4.84	0.54	0.77	
10.465	BB	0.48	617.93	115.29	98.42	
12.054	MM m	0.17	3.01	0.59	0.48	
12.899	MM m	0.21	2.08	0.35	0.33	
		**总和**	**627.86**			

HPLC purity of compound **10h**

单次进样报告

Agilent

**数据文件:**	2024-12-28 00-49-41+08-00-07.dx		
**序列名称:**	1260-2024-12-27 22-12-18+08-00		
**样品名称:**	ab-p-10		
**仪器:**	1260	**进样日期:**	2024-12-28 00:50:45+08:00
**进样体积:**	5.000 µL	**位置:**	P1-C3
**采集方法:**	normal.amx	**类型:**	样品
**处理方法:**	*Checkout.pmx	**样品含量:**	0.00
**手动修改:**	手动积分		

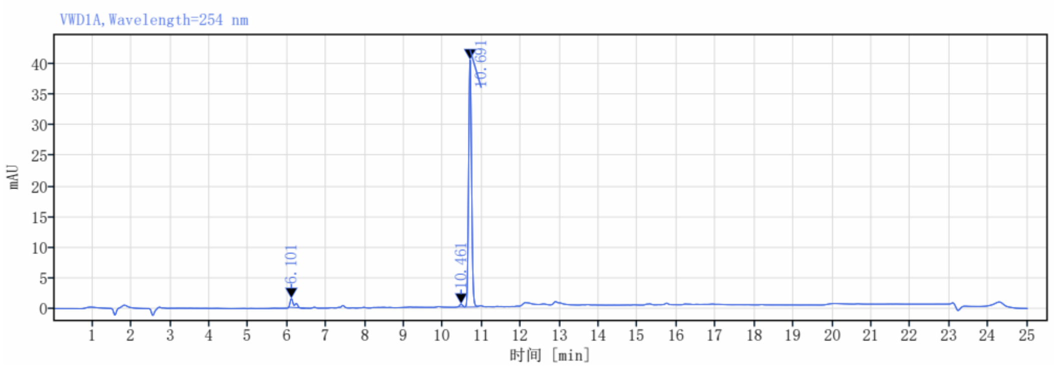

信号: VWD1A,Wavelength=254 nm

保留时间 [min]	类型	峰宽 [min]	峰面积	峰高	峰面积%	名称
6.101	MM m	0.42	10.41	1.41	4.52	
10.461	MM m	0.18	2.41	0.45	1.04	
10.691	VV	0.34	217.75	40.52	94.44	
		**总和**	**230.57**			

HPLC purity of compound **10i**

单次进样报告

**数据文件:**	2025-01-04 09-09-13+08-00-36.dx		
**序列名称:**	1260-2025-01-03 21-17-27+08-00		
**样品名称:**	ab-p-17		
**仪器:**	1260	**进样日期:**	2025-01-04 09:10:20+08:00
**进样体积:**	5.000 µL	**位置:**	P1-F10
**采集方法:**	normal.amx	**类型:**	样品
**处理方法:**	*Checkout.pmx	**样品含量:**	0.00
**手动修改:**	手动积分		

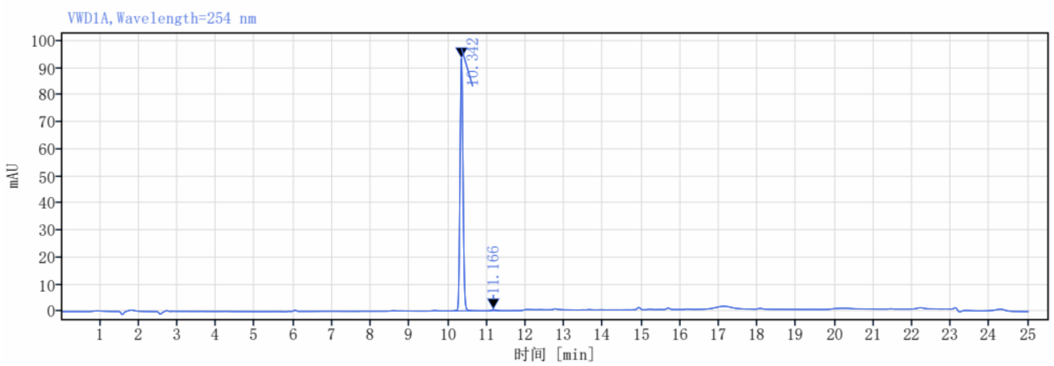

信号: VWD1A,Wavelength=254 nm

保留时间 [min]	类型	峰宽 [min]	峰面积	峰高	峰面积%	名称
10.342	BB	0.54	503.14	93.26	99.41	
11.166	MM m	0.30	2.98	0.46	0.59	
		**总和**	**506.12**			

HPLC purity of compound **10j**

单次进样报告

**数据文件:**	2025-01-06 18-18-55+08-00-63.dx		
**序列名称:**	1260-2025-01-05 20-56-54+08-00		
**样品名称:**	ab-p-15		
**仪器:**	1260	**进样日期:**	2025-01-06 18:20:04+08:00
**进样体积:**	5.000 µL	**位置:**	P1-F8
**采集方法:**	normal.amx	**类型:**	样品
**处理方法:**	Checkout.pmx	**样品含量:**	0.00
**手动修改:**	无		

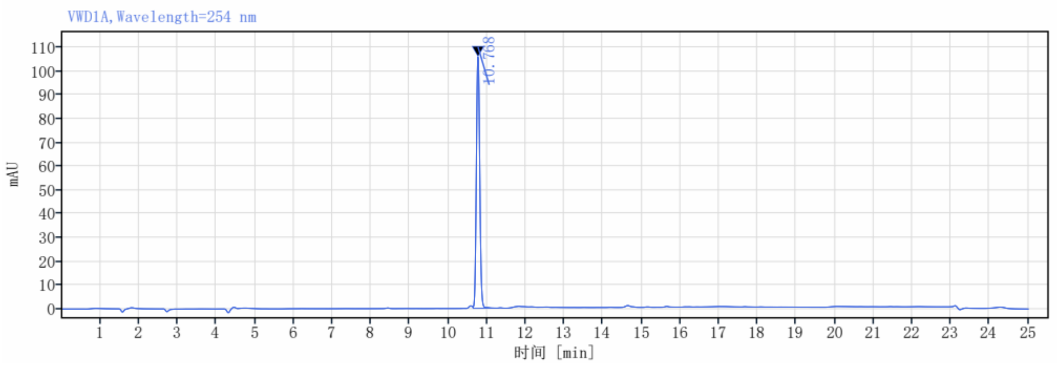

信号: VWD1A,Wavelength=254 nm

保留时间 [min]	类型	峰宽 [min]	峰面积	峰高	峰面积%	名称
10.768	VB	0.60	611.27	105.84	100.00	
		**总和**	**611.27**			

HPLC purity of compound **10k**

单次进样报告

**数据文件:**	2025-01-04 08-16-45+08-00-34.dx		
**序列名称:**	1260-2025-01-03 21-17-27+08-00		
**样品名称:**	ab-p-16		
**仪器:**	1260	**进样日期:**	2025-01-04 08:17:53+08:00
**进样体积:**	5.000 µL	**位置:**	P1-F9
**采集方法:**	normal.amx	**类型:**	样品
**处理方法:**	*Checkout.pmx	**样品含量:**	0.00
**手动修改:**	手动积分		

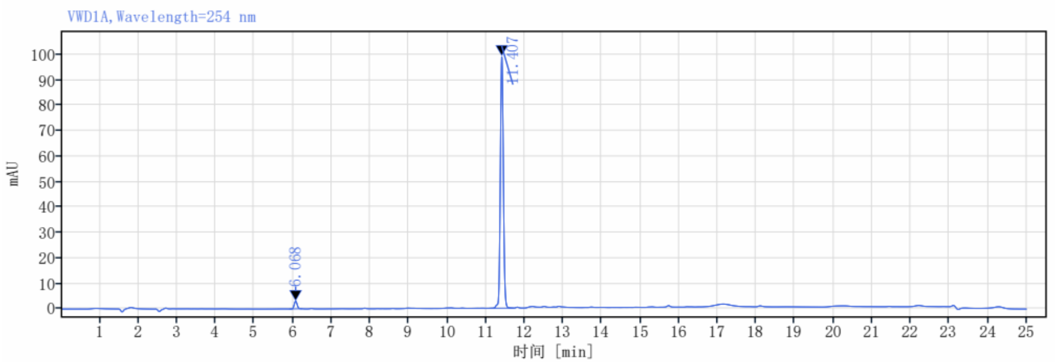

信号: VWD1A,Wavelength=254 nm

保留时间 [min]	类型	峰宽 [min]	峰面积	峰高	峰面积%	名称
6.068	MM m	0.33	16.37	3.05	2.93	
11.407	BV	0.65	541.86	99.09	97.07	
		**总和**	**558.22**			

HPLC purity of compound **10l**

单次进样报告

**数据文件:**	2024-12-23 15-45-44+08-00-11.dx		
**序列名称:**	1260-2024-12-23 11-23-42+08-00		
**样品名称:**	ab-p-8		
**仪器:**	1260	**进样日期:**	2024-12-23 15:46:50+08:00
**进样体积:**	5.000 µL	**位置:**	P1-B11
**采集方法:**	normal.amx	**类型:**	样品
**处理方法:**	*Checkout.pmx	**样品含量:**	0.00
**手动修改:**	手动积分		

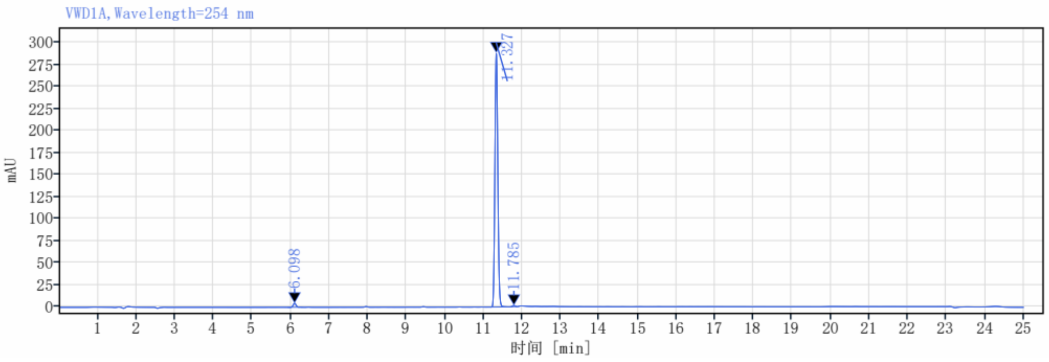

信号: VWD1A,Wavelength=254 nm

保留时间 [min]	类型	峰宽 [min]	峰面积	峰高	峰面积%	名称
6.098	MM m	0.38	24.14	4.48	1.56	
11.327	BB	0.48	1518.72	288.08	98.07	
11.785	MM m	0.18	5.71	1.32	0.37	
		**总和**	**1548.57**			

HPLC purity of compound **10m**

单次进样报告

**数据文件:**	2025-01-06 16-59-31+08-00-60.dx		
**序列名称:**	1260-2025-01-05 20-56-54+08-00		
**样品名称:**	ab-p-11		
**仪器:**	1260	**进样日期:**	2025-01-06 17:01:19+08:00
**进样体积:**	1.000 μL	**位置:**	P1-F6
**采集方法:**	normal.amx	**类型:**	样品
**处理方法:**	Checkout.pmx	**样品含量:**	0.00
**手动修改:**	无		

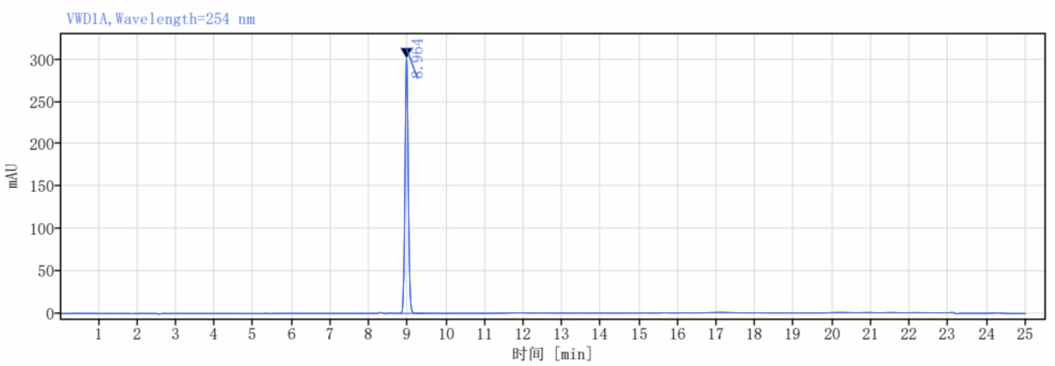

信号: VWD1A,Wavelength=254 nm

保留时间 [min]	类型	峰宽 [min]	峰面积	峰高	峰面积%	名称
8.964	BB	0.65	1767.65	300.82	100.00	
		**总和**	**1767.65**			

HPLC purity of compound **10n**

单次进样报告

**数据文件:**	2025-01-05 19-40-12+08-00-13.dx		
**序列名称:**	1260-2025-01-05 14-25-47+08-00		
**样品名称:**	ab-p-22		
**仪器:**	1260	**进样日期:**	2025-01-05 19:41:15+08:00
**进样体积:**	5.000 µL	**位置:**	P1-A7
**采集方法:**	normal.amx	**类型:**	样品
**处理方法:**	*Checkout.pmx	**样品含量:**	0.00
**手动修改:**	手动积分		

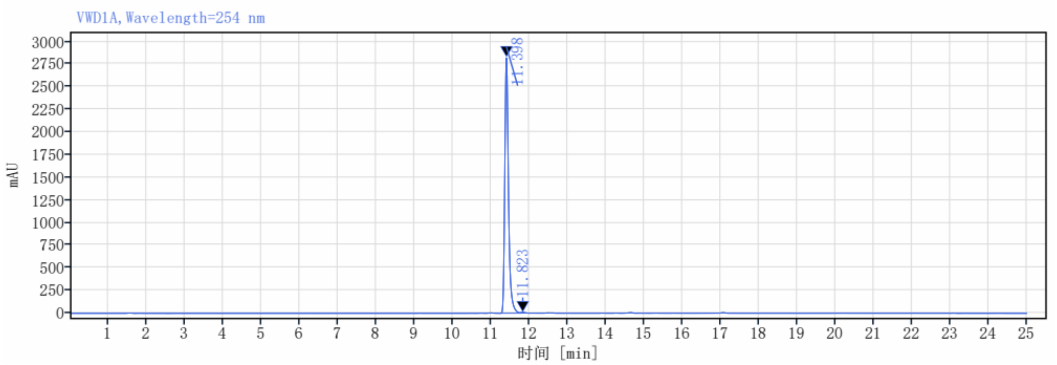

信号: VWD1A,Wavelength=254 nm

保留时间 [min]	类型	峰宽 [min]	峰面积	峰高	峰面积%	名称
11.398	BV	0.52	18861.72	2828.59	99.65	
11.823	MM m	0.19	66.47	10.53	0.35	
		**总和**	**18928.19**			

HPLC purity of compound **10o**

单次进样报告

**数据文件:**	2024-12-05 17-06-40+08-00-07.dx		
**序列名称:**	1260-2024-12-05 14-29-28+08-00		
**样品名称:**	ab-p-2		
**仪器:**	1260	**进样日期:**	2024-12-05 17:07:43+08:00
**进样体积:**	5.000 µL	**位置:**	P1-A4
**采集方法:**	normal.amx	**类型:**	样品
**处理方法:**	*Checkout.pmx	**样品含量:**	0.00
**手动修改:**	手动积分		

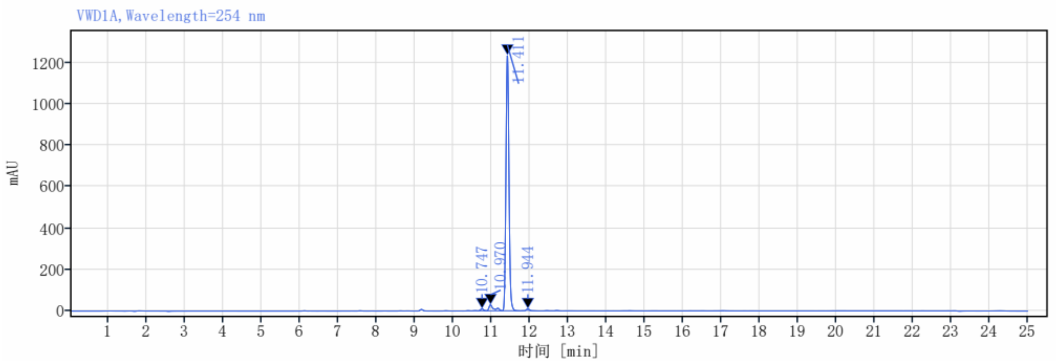

信号: VWD1A,Wavelength=254 nm

保留时间 [min]	类型	峰宽 [min]	峰面积	峰高	峰面积%	名称
10.747	MM m	0.19	47.97	9.25	0.68	
10.970	VM m	0.41	262.19	28.94	3.69	
11.411	BB	0.45	6766.44	1232.95	95.23	
11.944	MM m	0.13	28.81	6.88	0.41	
		**总和**	**7105.42**			

HPLC purity of compound **10p**

单次进样报告

Agilent

**数据文件:**	2025-01-05 18-47-49+08-00-11.dx		
**序列名称:**	1260-2025-01-05 14-25-47+08-00		
**样品名称:**	ab-p-21		
**仪器:**	1260	**进样日期:**	2025-01-05 18:48:53+08:00
**进样体积:**	5.000 µL	**位置:**	P1-A6
**采集方法:**	normal.amx	**类型:**	样品
**处理方法:**	*Checkout.pmx	**样品含量:**	0.00
**手动修改:**	手动积分		

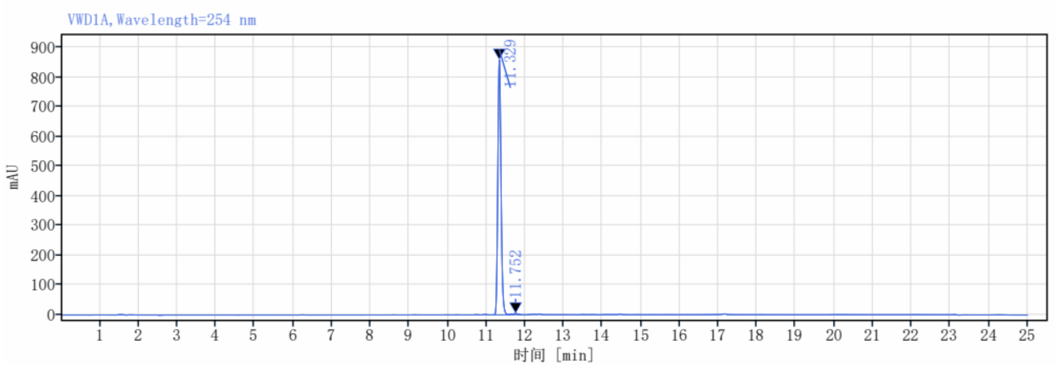

信号: VWD1A, Wavelength=254 nm

保留时间 [min]	类型	峰宽 [min]	峰面积	峰高	峰面积%	名称
11.329	BV	0.43	4967.99	859.71	99.10	
11.752	MM m	0.33	44.98	4.45	0.90	
		**总和**	**5012.97**			

HPLC purity of compound **10q**

单次进样报告

**数据文件:**	2025-01-05 17-03-04+08-00-07.dx		
**序列名称:**	1260-2025-01-05 14-25-47+08-00		
**样品名称:**	ab-p-19		
**仪器:**	1260	**进样日期:**	2025-01-05 17:04:07+08:00
**进样体积:**	5.000 µL	**位置:**	P1-A4
**采集方法:**	normal.amx	**类型:**	样品
**处理方法:**	*Checkout.pmx	**样品含量:**	0.00
**手动修改:**	手动积分		

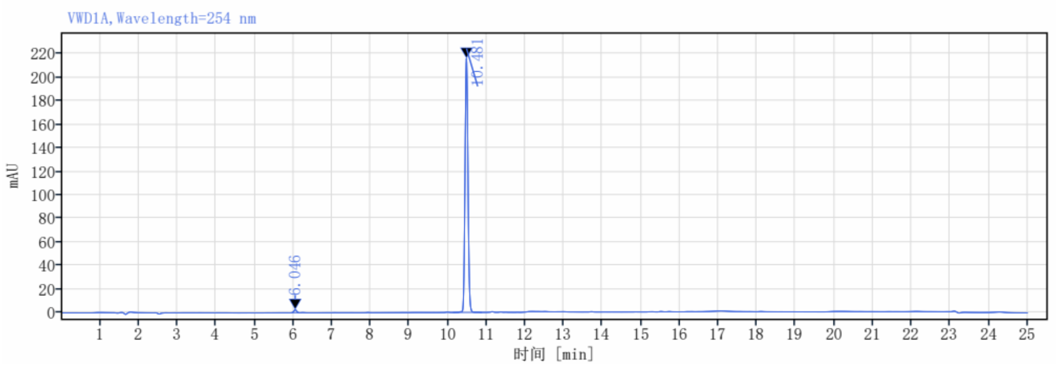

信号: VWD1A,Wavelength=254 nm

保留时间 [min]	类型	峰宽 [min]	峰面积	峰高	峰面积%	名称
6.046	MM m	0.17	12.15	2.44	1.04	
10.481	BB	0.75	1156.36	215.77	98.96	
		**总和**	**1168.52**			

HPLC purity of compound **10r**

单次进样报告

Agilent

**数据文件:**	2025-01-04 06-31-47+08-00-30.dx		
**序列名称:**	1260-2025-01-03 21-17-27+08-00		
**样品名称:**	ab-p-14		
**仪器:**	1260	**进样日期:**	2025-01-04 06:32:55+08:00
**进样体积:**	5.000 µL	**位置:**	P1-F7
**采集方法:**	normal.amx	**类型:**	样品
**处理方法:**	*Checkout.pmx	**样品含量:**	0.00
**手动修改:**	手动积分		

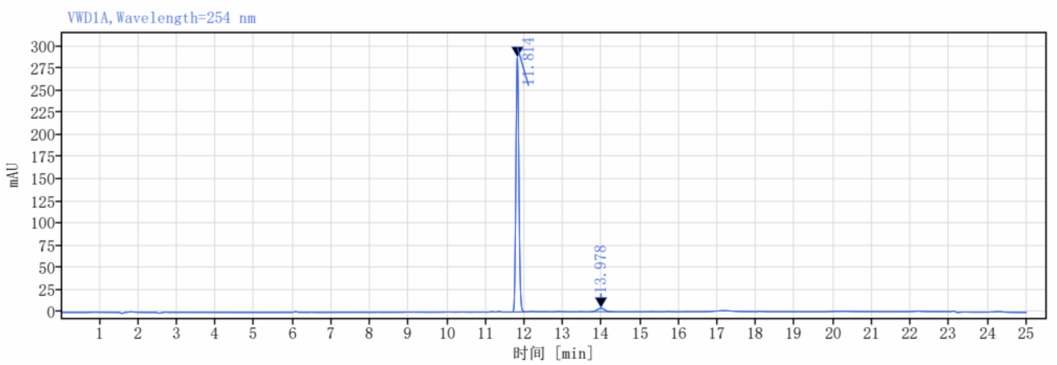

信号: VWD1A,Wavelength=254 nm

保留时间 [min]	类型	峰宽 [min]	峰面积	峰高	峰面积%	名称
11.814	BV	0.36	1533.28	286.91	96.70	
13.978	MM m	0.56	52.25	3.93	3.30	
		**总和**	**1585.53**			

HPLC purity of compound **10s**

单次进样报告

**数据文件:**	2025-01-04 05-39-17+08-00-28.dx		
**序列名称:**	1260-2025-01-03 21-17-27+08-00		
**样品名称:**	ab-p-13		
**仪器:**	1260	**进样日期:**	2025-01-04 05:40:27+08:00
**进样体积:**	5.000 μL	**位置:**	P1-F6
**采集方法:**	normal.amx	**类型:**	样品
**处理方法:**	*Checkout.pmx	**样品含量:**	0.00
**手动修改:**	手动积分		

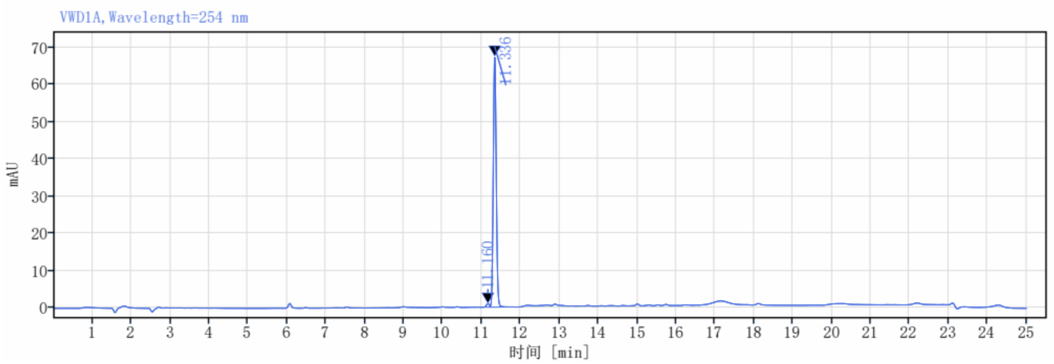

信号: VWD1A,Wavelength=254 nm

保留时间 [min]	类型	峰宽 [min]	峰面积	峰高	峰面积%	名称
11.160	MM m	0.15	4.57	0.92	1.24	
11.336	VB	0.49	362.91	67.31	98.76	
		**总和**	**367.48**			

HPLC purity of compound **10t**

单次进样报告

**数据文件:**	2025-01-06 17-26-26+08-00-61.dx		
**序列名称:**	1260-2025-01-05 20-56-54+08-00		
**样品名称:**	ab-p-12		
**仪器:**	1260	**进样日期:**	2025-01-06 17:27:35+08:00
**进样体积:**	1.000 µL	**位置:**	P1-F7
**采集方法:**	normal.amx	**类型:**	样品
**处理方法:**	Checkout.pmx	**样品含量:**	0.00
**手动修改:**	无		

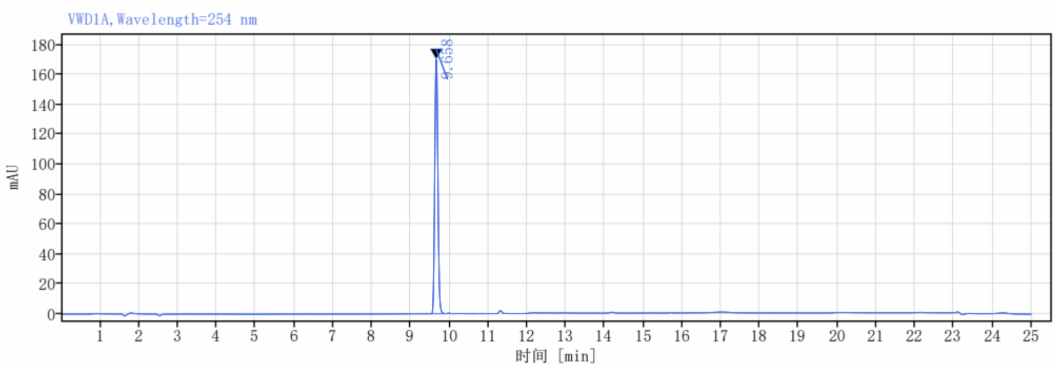

信号: VWD1A,Wavelength=254 nm

保留时间 [min]	类型	峰宽 [min]	峰面积	峰高	峰面积%	名称
9.658	BV	0.37	902.03	169.84	100.00	
		**总和**	**902.03**			

HPLC purity of compound **10u**

单次进样报告

Agilent

**数据文件:**	2024-12-27 23-04-51+08-00-03.dx		
**序列名称:**	1260-2024-12-27 22-12-18+08-00		
**样品名称:**	ab-p-7		
**仪器:**	1260	**进样日期:**	2024-12-27 23:05:56+08:00
**进样体积:**	5.000 µL	**位置:**	P1-C1
**采集方法:**	normal.amx	**类型:**	样品
**处理方法:**	*Checkout.pmx	**样品含量:**	0.00
**手动修改:**	无		

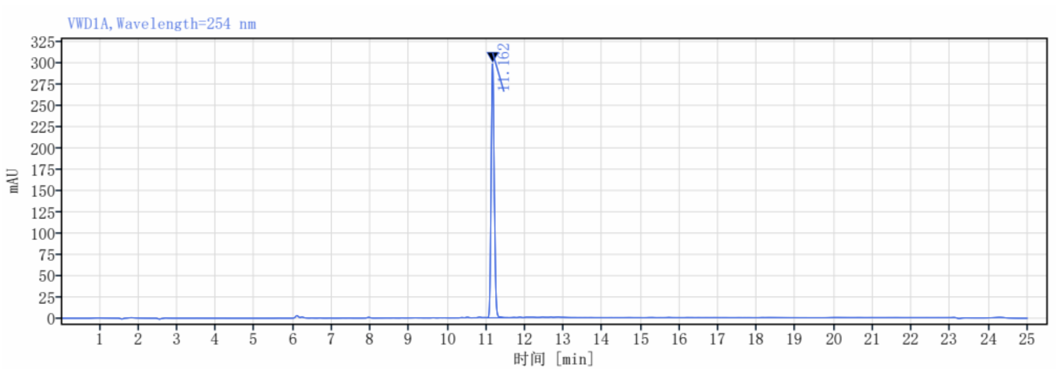

信号: VWD1A,Wavelength=254 nm

保留时间 [min]	类型	峰宽 [min]	峰面积	峰高	峰面积%	名称
11.162	VB	0.54	1621.97	299.69	100.00	
		**总和**	**1621.97**			

HPLC purity of compound **10v**

单次进样报告

**数据文件:**	2025-01-06 18-45-11+08-00-64.dx		
**序列名称:**	1260-2025-01-05 20-56-54+08-00		
**样品名称:**	ab-p-20		
**仪器:**	1260	**进样日期:**	2025-01-06 18:46:19+08:00
**进样体积:**	5.000 µL	**位置:**	P1-F9
**采集方法:**	normal.amx	**类型:**	样品
**处理方法:**	Checkout.pmx	**样品含量:**	0.00
**手动修改:**	无		

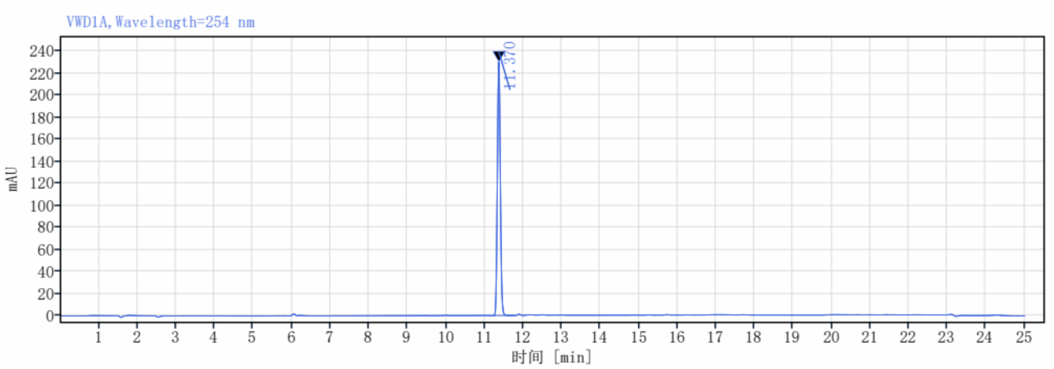

信号: VWD1A, Wavelength=254 nm

保留时间 [min]	类型	峰宽 [min]	峰面积	峰高	峰面积%	名称
11.370	BB	0.55	1238.37	229.63	100.00	
		**总和**	**1238.37**			

HPLC purity of compound **10w**

单次进样报告

**数据文件:**	2025-01-05 16-10-41+08-00-05.dx		
**序列名称:**	1260-2025-01-05 14-25-47+08-00		
**样品名称:**	ab-p-18		
**仪器:**	1260	**进样日期:**	2025-01-05 16:11:44+08:00
**进样体积:**	5.000 µL	**位置:**	P1-A3
**采集方法:**	normal.amx	**类型:**	样品
**处理方法:**	*Checkout.pmx	**样品含量:**	0.00
**手动修改:**	手动积分		

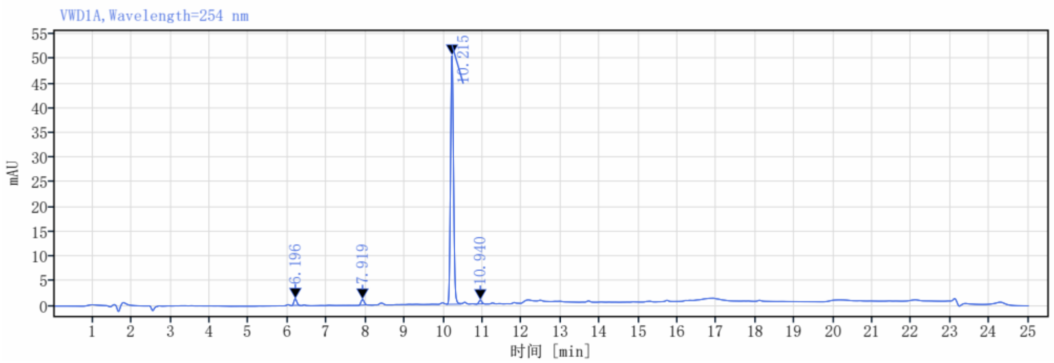

信号: VWD1A, Wavelength=254 nm

保留时间 [min]	类型	峰宽 [min]	峰面积	峰高	峰面积%	名称
6.196	MM m	0.18	6.76	1.30	2.37	
7.919	MM m	0.19	5.93	1.12	2.08	
10.215	VV	0.37	268.84	50.34	94.20	
10.940	MM m	0.19	3.88	0.74	1.36	
		**总和**	**285.41**			
